# Endothelial c-Maf prevents MASLD-like liver fibrosis by regulating chromatin accessibility to suppress pathogenic microvascular cell subsets

**DOI:** 10.1016/j.jhepr.2025.101475

**Published:** 2025-06-06

**Authors:** Manuel Winkler, Theresa Staniczek, Maximilian Suhayda, Sina Wietje Kürschner-Zacharias, Johannes Hoffmann, Julio Cordero, Linda Kraske, Hannah Maude, Dorka Nagy, Rita Manco, Carsten Sticht, Michelle Neßling, Karsten Richter, Gergana Dobreva, Anna Maria Randi, Inês Cebola, Kai Schledzewski, Philipp-Sebastian Reiners-Koch, Sergij Goerdt, Christian David Schmid

**Affiliations:** 1 Department of Dermatology, Venereology and Allergology, University Medical Center and Medical Faculty Mannheim, Heidelberg University, and Center of Excellence in Dermatology, Mannheim, Germany; 2 Department of Anatomy and Developmental Biology, European Center for Angioscience, Medical Faculty Mannheim, Heidelberg University, Mannheim, Germany; 3 Section of Genetics and Genomics, Department of Metabolism, Digestion and Reproduction, Imperial College London, London, United Kingdom; 4 National Heart and Lung Institute, Imperial College London, London, United Kingdom; 5 Laboratory of Hepato-Gastroenterology, Institut de Recherche Expérimentale et Clinique (IREC), UCLouvain; Brussels, Belgium; 6 Core Facility Next Generation Sequencing, Medical Faculty Mannheim, Heidelberg University, Mannheim, Germany; 7 Central Unit Electron Microscopy, German Cancer Research Center (DKFZ), Heidelberg, Germany; 8 European Center for Angioscience, Medical Faculty Mannheim, Heidelberg University, Mannheim, Germany; 9 Department of Dermatology and Allergy, Evangelisches Krankenhaus Düsseldorf, Düsseldorf, Germany

**Keywords:** Cirrhosis, Liver sinusoidal endothelial cells (LSEC), Capillarization, Single-cell RNA-Seq analysis, ATAC-Seq analysis

## Abstract

**Background & Aims:**

Liver sinusoidal endothelial cells (LSECs) are highly specialized components of the hepatic vascular niche, regulating liver function and disease pathogenesis through angiocrine signaling. Recently, we identified GATA4 as a key transcription factor controlling LSEC development and protecting against liver fibrosis. As the transcription factor c-Maf was strongly downregulated in *Gata4*-deficient LSECs, we hypothesized that c-Maf might be an important downstream effector of GATA4 in LSEC differentiation and liver fibrogenesis.

**Methods:**

*Clec4g-iCre/Maf*^*fl/fl*^ (*Maf*^*LSEC-KO*^) mice with LSEC-specific *Maf* deficiency were generated and liver tissue was analyzed histologically. LSECs were isolated for bulk RNA-seq, ATAC-seq, and single-cell (sc) RNA-seq analysis. *Maf*^*LSEC-KO*^ livers were analyzed after MASH diet feeding. The expression of *MAF* and its targets was analyzed in published human scRNA-seq data.

**Results:**

Endothelial *Maf* deficiency resulted in perisinusoidal liver fibrosis (Sirius red 0.46% *vs*. 2.92%; *p* <0.05) without affecting metabolic liver zonation, accompanied by a switch from sinusoidal to continuous endothelial cell identity, which was aggravated upon MASH diet feeding (*p* <0.01). Furthermore, endothelial *Maf* deficiency caused LSEC proliferation (*p <*0.05) and expression of profibrotic angiocrine factors including *Pdgfb*, *Igfbp5*, *Flrt2, and Cxcl12*, among which FLRT2 (*p* <0.01) and CXCL12 (*p* <0.001) activated hepatic stellate cells *in vitro*. scRNA-seq revealed replacement of zonated LSEC subpopulations with capillarized, proliferative, sprouting and secretory endothelial cell subsets that promote liver fibrogenesis and angiogenesis. This fundamental dysregulation of LSEC gene expression and differentiation was caused by changes in chromatin accessibility and transcription factor activity following loss of *Maf*. Notably, endothelial *MAF* expression was also significantly reduced in human cirrhotic livers (*p <*0.0001).

**Conclusions:**

Hepatic endothelial c-Maf protects against metabolic dysfunction-associated steatohepatitis-like liver fibrosis and regulates endothelial differentiation and zonation by controlling chromatin opening.

**Impact and implications:**

This work builds on the known importance of liver sinusoidal endothelial cells in liver function and disease. Here, transcription factor c-Maf is identified as a master regulator in maintaining normal differentiation and zonation of liver sinusoidal endothelial cells, thereby protecting against the development of liver fibrosis/cirrhosis. The findings are significant for researchers and clinicians focusing on liver disease, as they suggest potential new targets for therapeutic intervention. These findings could instruct the development of novel preventive treatment options and antifibrotic therapy regimens as well as liver repair strategies, benefiting patients, clinicians and policy makers in the management of liver disease.

## Highlights

• Endothelial c-Maf prevents MASLD-like liver fibrosis.
• c-Maf is a master regulator of LSEC identity.
• c-Maf prevents development of pathogenic profibrotic endothelial cell subsets.
• c-Maf controls chromatin accessibility of LSEC.
• *MAF* is downregulated in human cirrhosis.

## Graphical abstract

**Figure:**
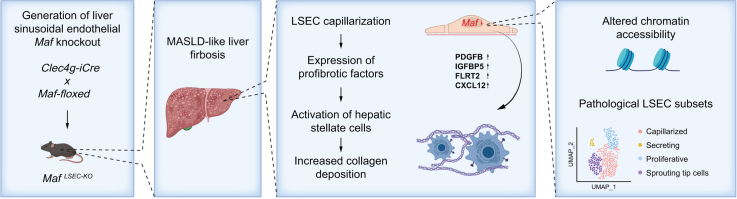

## Introduction

Liver sinusoidal endothelial cells (LSECs) represent a distinct organotypic endothelial cell (EC) subtype showing remarkable morphological and functional specialization. LSECs are discontinuous, exhibiting fenestrations and lacking a basement membrane, and together with resident hepatic macrophages, *i.e*. hepatic Kupffer cells, form a sinusoidal interface that allows direct interactions with stellate cells and hepatocytes, as well as easy exchange of blood cellular elements, nutrients and metabolites. LSECs play a central role in regulating blood flow and portal pressure, as well as in hepatic immune surveillance.^1^ In addition, LSECs control liver functions via angiocrine signaling. We and others have shown, for example, that iron metabolism is regulated by LSEC angiokines BMP2^2^ and BMP6,^3^ whereas metabolic liver zonation is controlled by angiocrine Wnt signaling.^4^

The establishment and maintenance of LSEC identity are regulated by signaling pathways and endothelial transcription factors. For example, we have shown that ALK1 signaling regulates hepatic vessel formation, angiodiversity, and angiocrine functions, thereby preventing hereditary hemorrhagic telangiectasia of the liver.^5^ Additionally, both our group and others have demonstrated the critical role of balanced Notch signaling in maintaining LSEC integrity.^6^^,^^7^

Few transcription factors have been described to control LSEC differentiation and liver functions. Loss of the ubiquitous endothelial transcription factor ERG caused endothelial-mesenchymal transition in LSECs and led to the development of periportal liver fibrosis.^8^ Our group identified GATA4 as a transcription factor specifically expressed in LSECs (compared to endothelial cells in most other vascular beds). As assessed in *Stab2-cre/Gata4*^*fl/fl*^ mice with loss of *Gata4* in endothelial cells early in embryonic development, we demonstrated that GATA4 controls embryonic LSEC differentiation and fetal hematopoiesis.^9^ Using *Clec4g-iCre/Gata4*^*fl/fl*^ mice with liver endothelial-specific loss of *Gata4* late in fetal life, we demonstrated that endothelial GATA4 controls metabolic dysfunction-associated steatohepatitis (MASH)-like perisinusoidal liver fibrosis by preventing a pathogenic switch in angiocrine signaling.^10^ Notably, in the latter study, endothelial *Maf* expression was significantly downregulated in LSECs from *Clec4g-iCre/Gata4*^*fl/fl*^ mice, indicating that c-Maf might be a downstream effector of GATA4 that regulates LSEC differentiation and function.

Gómez-Salinero *et al.* have shown that specification of fetal liver endothelial progenitors to functional, zonated adult sinusoids requires c-Maf. However, loss of *Maf* in the inducible *VE-cadherin-CreERT2* model was not accompanied by development of either spontaneous periportal or perisinusoidal liver fibrosis, while carbon tetrachloride-induced periportal liver fibrosis was aggravated.^11^

Here, using *Clec4g-iCre/Maf*^*fl/fl*^ (*Maf*^*LSEC-KO*^) mice with a high penetrance of excision from late fetal life onwards in a LSEC-selective manner, we demonstrate that loss of liver sinusoidal endothelial *Maf* caused MASH-like perisinusoidal liver fibrosis. Perisinusoidal liver fibrosis was driven by sinusoidal capillarization and by a switch towards expression of pro-fibrotic angiokines. Single-cell (sc) RNA sequencing (RNA-seq) analysis of LSECs revealed a fundamental loss of LSEC identity and replacement of normal LSEC subpopulations with capillarized, proliferative, sprouting and secretory hepatic microvascular EC subsets in *Maf*^*LSEC-KO*^ mice. Finally, we show that endothelial c-Maf protects from liver fibrosis by controlling LSEC chromatin accessibility, promoting hepatic sinusoidal endothelial *vs*. continuous endothelial identity and suppressing activation of profibrotic and angiogenic endothelial gene programs in the liver.

## Materials and methods

### Ethical compliance

The experimental protocols used in this study complied with national and international ethical guidelines and, in case of animal models, were approved by the animal welfare commission of the Regierungspraesidium Karlsruhe (Karlsruhe, Germany).

### Animal models

Female and male mice aged 3 and 6 months were used in this study. Mice were hosted under specific pathogen-free conditions in single ventilated cages in a 12 h/12 h day/night cycle and fed *ad libitum* with a standard rodent diet (V1534-000, Ssniff) with free access to water.

For the generation of liver sinusoidal endothelial conditional *Maf*-knockout mice, *Clec4g-iCre* mice (C57BL/6N-Tg(*Clec4g*-icre)1.1Sgoe, MGI:6280453)^7^ were crossed with *Maf*-floxed mice (B6.129P2-*Maf*^*tm2.1Cbm*^, MGI:5316775).^12^ Mice with the genotype *Clec4g-iCre*^*tg/wt*^ *x Maf*^*fl/fl*^ indicating homozygous recombination were denoted as *Maf*^*LSEC-KO*^. In all experiments, the littermates with the two genotypes *Clec4g-iCre*^*wt/wt*^ *x Maf*^*fl/fl*^ and *Clec4g-iCre*^*wt/wt*^ *x Maf*^*fl/wt*^ were used as controls.

### Statistical analysis

Statistical analyses were conducted in R 4.1.2 (R Core Team) and Prism 10 (GraphPad Software). For sample size calculation, we suggested an α-level of 0.05 and a β-level of 0.2, while the power was adjusted for each experiment. Welch’s *t* test and Mann-Whitney *U* test were used for statistical testing. Two-way ANOVA followed by Tukey’s *post hoc* test was used for statistical comparison of more than two groups with two independent variables. A *p* value of <0.05 was considered statistically significant. The appropriate statistical test was chosen according to the requirements of each test (*e.g.* normal distribution). Normal distribution was assessed using the Shapiro-Wilk test.

### Additional methodological details

For further information on materials and methods, please refer to the supplementary data.

## Results

### Endothelial *Maf* deficiency causes perisinusoidal liver fibrosis without affecting metabolic liver zonation

To investigate the role of c-Maf in LSEC differentiation and liver function, we generated a novel mouse model by crossing *Maf*-floxed mice with *Clec4g-iCre* driver mice resulting in *Clec4g-iCre/Maf*^*fl/fl*^ (*Maf*^*LSEC-KO*^) mice. *Maf*^*LSEC-KO*^ mice were born in approximately the expected Mendelian ratio and had a normal lifespan (Fig. S1A and B). At 12 weeks of age, body weight, liver weight and liver/body weight ratio were not significantly altered in *Maf*^*LSEC-KO*^ mice (Fig. S1C).

**Fig. 1:**
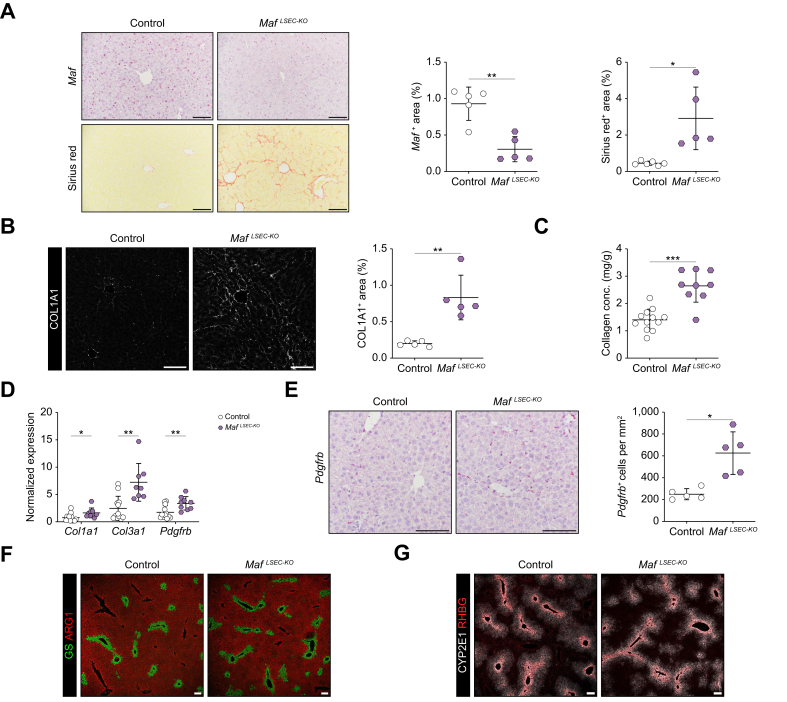
Endothelial *Maf* deficiency leads to perisinusoidal liver fibrosis while metabolic zonation is preserved. (A) *Maf* ISH and Sirius red staining with quantification (n = 5, 6). (B) Immunofluorescence staining and quantification of COL1A1 (n = 5). (C) Tissue collagen assay of livers (n = 9, 12). (D) qPCR for *Col1a1*, *Col3a1* and *Pdgfrb* using RNA from whole liver (n = 8). (E) *Pdgfrb* ISH in mouse livers (n = 5). (F) Immunofluorescence staining of GS and ARG1 (n = 8); and (G) CYP2E1 and RHBG in livers (n = 5). Scale bars: 100 μm. Mean ± SD. (A-E) Welch’s *t* test; *∗p <*0.05; ∗*∗p <*0.01; ∗∗*∗p <*0.001. ISH, *in situ* hybridization.

Because of *Maf* loss in the liver endothelium (Fig. 1A, upper panel), we detected significantly increased deposition of extracellular matrix proteins, as assessed by Sirius red staining of liver samples, mainly located in midzonal areas of the liver, *i.e.* perisinusoidal liver fibrosis (Fig. 1A, lower panel). Consistent with these observations, immunofluorescence (IF) staining showed significantly increased perivascular deposition of collagens 1 and 4 (COL1A1 and COL4A1) (Figs 1B and S1D). A collagen assay using whole liver lysate confirmed an increase in the amount of collagen in *Maf*^*LSEC-KO*^ livers (Fig. 1C). In addition, qPCR analysis of whole liver lysate showed increased *Col1a1* and *Col3a1* expression (Fig. 1D). Notably, IF for SMA and PDGBRB, as well as *in situ* hybridization (ISH) for *Pdgfrb,* showed an increase in the number of activated stellate cells, intricately involved in the development of liver fibrosis (Figs 1E and S1E). The increased number of activated stellate cells was consistent with an increase in *Pdgfrb* expression on qPCR from whole liver lysate (Fig. 1D), while expression of other stellate cell marker genes was not enhanced (Fig. S1F).

To define the cell type of origin for the different collagen subtypes enriched in *Maf*^*LSEC-KO*^ livers, namely *Col1a1*, *Col3a1*, and *Col4a1*, we performed FISH (fluorescence ISH) (Fig. S2A–C). We found that *Col1a1* and *Col3a1* are mainly produced by hepatic stellate cells (HSCs), whereas *Col4a1* is mainly derived from ECs (Fig. S2A–B).

Liver enzymes, including alanine aminotransferase, aspartate aminotransferase and glutamate dehydrogenase were slightly elevated in the plasma of *Maf*^*LSEC-KO*^ mice indicating moderate hepatopathy, which is often associated with perisinusoidal liver fibrosis, while other standard plasma values were normal (Fig. S3A). Immunofluorescent analysis using F4/80 and CD11b antibodies showed a significant increase in monocyte/macrophage numbers in *Maf*^*LSEC-KO*^ livers, indicating hepatic inflammation, which often accompanies and aggravates liver fibrosis (Fig. S3B).

On the contrary, further staining including H&E, Oil red O and Prussian blue, as well as a triglyceride assay on whole liver lysate, did not reveal hepatic steatosis or iron deposition in *Maf*^*LSEC-KO*^ livers (Fig. S3C and D). As *Maf* is known to be mainly expressed in midzonal LSECs, it was not unexpected that metabolic liver zonation, which is controlled by pericentral angiocrine factors, was also normal, as shown by immunofluorescence staining for GS, ARG1, CYP2E1, and RHBG (Figs 1F,G and S3E).

### Endothelial *Maf* deficiency leads to a shift from discontinuous to continuous marker expression, entailing sinusoidal capillarization, and to upregulation of profibrotic angiocrine factors

**Fig. 2:**
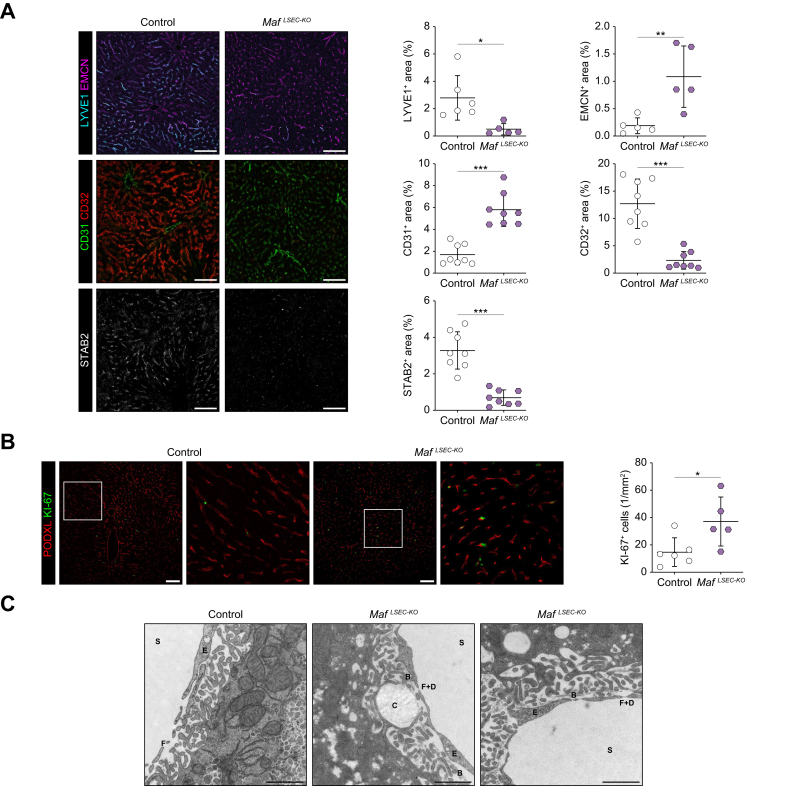
Upregulation of continuous endothelial marker genes and proliferation marker Ki-67 in LSECs from *Maf*^*LSEC-KO*^ mice. (A) Immunofluorescence staining and quantification of LYVE1, EMCN, CD31, CD32, and STAB2 in livers (n = 5, 6, 8). (B) Immunofluorescence staining for Ki-67 and pan-endothelial marker PODXL and Ki-67 quantification (n = 5, 6). (C) TEM of livers (S = sinusoidal lumen, E = endothelial cell, F = fenestration, D = diaphragm, B = basement membrane, C = collagen fiber) (n = 1, 2). (A-B) Scale bars: 100 μm; (C) scale bars: 1 μm. Mean ± SD. (A [LYVE1, EMCN, STAB2], B) Welch’s *t* test; (A [CD31, CD32]) Mann-Whitney *U* test; *∗p <*0.05; ∗*∗p <*0.01; ∗∗*∗p <*0.001. LSECs, liver sinusoidal endothelial cells; TEM, transmission electron microscopy.

When analyzing LSEC differentiation in *Maf*^*LSEC-KO*^ mice *in situ*, we detected a shift from discontinuous to continuous endothelial marker expression. IF staining showed that while LYVE1, CD32, and STAB2 were significantly downregulated, EMCN and CD31 were significantly upregulated along the whole length of the liver sinuses (Fig. 2A).

Interestingly, immunofluorescent staining for Ki-67 and pan-endothelial marker PODXL showed significantly increased cell proliferation of LSECs in *Maf*^*LSEC-KO*^ mice (Fig. 2B).

To examine the ultrastructural changes in LSECs upon *Maf* deficiency, we performed transmission electron microscopy (Figs 2C and S4A). LSECs in *Maf*^*LSEC-KO*^ mice were lined by a basement membrane, and fibrous collagen deposition was seen in the space of Disse (Fig. 2C), which is the ultrastructural correlate of the perisinusoidal liver fibrosis described above (Fig. 1A). In addition, *Maf*^*LSEC-KO*^ LSECs showed fenestrae that were covered by a diaphragm (Fig. 2C). Thus, our transmission electron microscopy results confirm development of sinusoidal capillarization not only at the marker level, but also at the ultrastructural level.

**Fig. 3:**
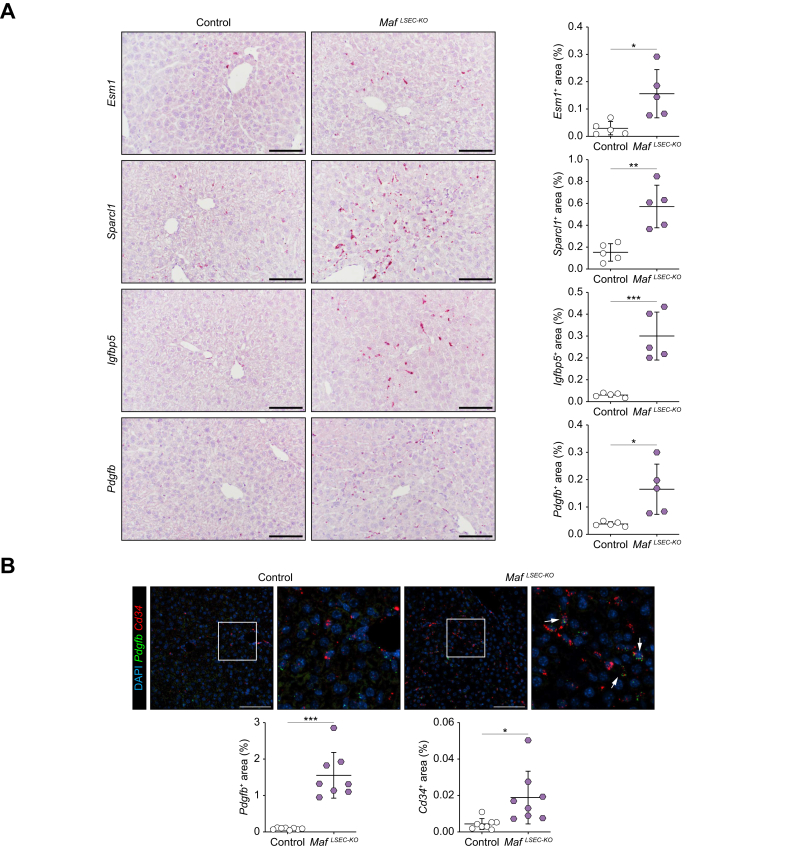
Endothelial *Maf* deficiency causes increased expression of profibrotic angiocrine factors. (A) ISH and quantification for *Esm1, Sparcl1, Igfbp5,* and *Pdgfb* of livers (n = 5). (B) FISH and quantification for *Pdgfb* and *Cd34* of livers (n = 8). Scale bars: 100 μm. Mean ± SD. (A [*Igfbp5, Pdgfb*], B [*Pdgfb*]) Welch’s *t* test; (A [*Esm1, Sparcl1*], B [*Cd34*]) Mann-Whitney *U* test; *∗p <*0.05; ∗*∗p <*0.01; ∗∗*∗p <*0.001. FISH, fluorescence ISH; ISH, *in situ* hybridization.

In addition to sinusoidal capillarization, the known profibrotic angiocrine factors *Esm1*, *Sparcl1*, *Igfbp5,* and *Pdgfb* were significantly upregulated in LSECs in *Maf*^*LSEC-KO*^ livers as seen by ISH, likely contributing to a profibrotic milieu around the hepatic sinus (Fig. 3A). Endothelial *Pdgfb* expression was confirmed using *Cd34*-*Pdgfb* FISH (Fig. 3B).

### Loss of endothelial *Maf* aggravates liver fibrosis in a dietary model of MASH

**Fig. 4:**
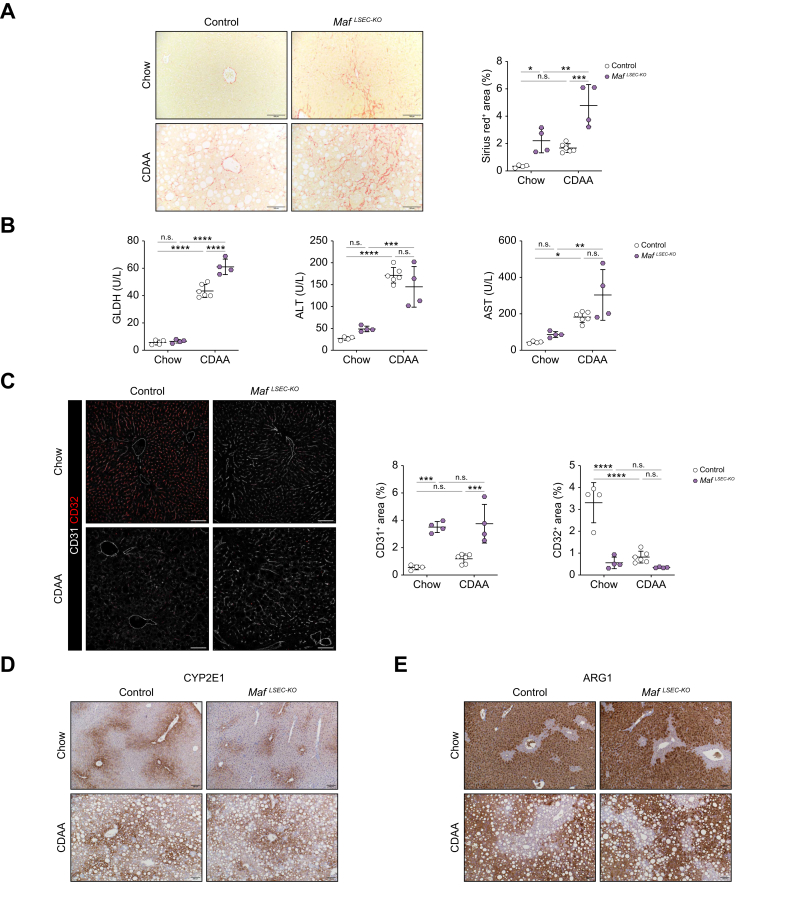
*Maf* knockout aggravates MASH diet-induced liver fibrosis. (A) Sirius red staining and quantification of control and *Maf*^*LSEC-KO*^ livers after Chow or CDAA diet for 10 weeks (n = 4, 6). (B) Blood plasma levels of GLDH, ALT, and AST in control and *Maf*^*LSEC-KO*^ mice (n = 4, 6). (C) Immunofluorescence staining and quantification for CD31 and CD32 (n = 4, 6). (D) CYP2E1 and (E) ARG1 Immunohistochemistry staining of control and *Maf*^*LSEC-KO*^ livers after Chow or CDAA diet for 10 weeks (n = 4, 6). Scale bars: 100 μm. (A-C) two-way ANOVA, Tukey’s *post hoc* test; n.s. *p* ≥0.05; *∗p <*0.05; ∗*∗p <*0.01; ∗∗*∗p <*0.001.; ∗∗∗*∗p <*0.0001. ALT, alanine aminotransferase; AST, aspartate aminotransferase; CDAA, choline-deficient, L-amino acid-defined; GLDH, glutamate dehydrogenase; LSECs, liver sinusoidal endothelial cells; MASH, metabolic dysfunction-associated steatohepatitis.

To investigate whether endothelial *Maf* deficiency aggravates liver injury in a dietary MASH model with late development of liver fibrosis, we fed *Maf*^*LSEC-KO*^ and control mice a choline-deficient, L-amino acid-defined (CDAA) diet for 10 weeks. Basic animal data such as body weight and liver weight, were not significantly altered while liver/body weight ratio was slightly increased in CDAA-fed *vs*. Chow-fed control animals (Fig. S4B). On the contrary, livers from *Maf*^*LSEC-KO*^ mice fed a CDAA diet did not only show significantly increased extracellular matrix deposition on Sirius red staining compared to CDAA-fed control animals, but also compared to Chow-fed *Maf*^*LSEC-KO*^ mice (Fig. 4A). The increased susceptibility of *Maf*^*LSEC-KO*^ livers to MASH-related injury was also reflected by significantly increased levels of hepatocyte damage marker glutamate dehydrogenase in the peripheral blood of CDAA-fed *Maf*^*LSEC-KO*^ mice compared to both CDAA-fed control animals and to Chow-fed *Maf*^*LSEC-KO*^ mice, while alanine and aspartate aminotransferase showed no significant differences between CDAA-fed control animals and CDAA-fed *Maf*^*LSEC-KO*^ mice (Fig. 4B).

IF indicated that the level of sinusoidal capillarization of LSECs in *Maf*^*LSEC-KO*^ mice was not further enhanced by CDAA diet feeding, as CD31 upregulation and CD32 loss were not significantly different between Chow-fed and CDAA-fed *Maf*^*LSEC-KO*^ mice, indicating that endothelial *Maf* deficiency *per se* caused a maximal effect (Fig. 4C). Furthermore, metabolic zonation of the liver was conserved upon CDAA diet feeding in both *Maf*^*LSEC-KO*^ and control livers as illustrated by pericentral markers CYP2E1 and GS, as well as periportal markers ARG1 and HAL (Figs 4D,E and S4C,-D).

In conclusion, dietary induction of MASH aggravated the degree of liver fibrosis caused by endothelial *Maf* deficiency in the liver. However, most other parameters analyzed in our basic characterization remained unaffected. Therefore, further experiments were performed exclusively with Chow-fed *Maf*^*LSEC-KO*^ and control mice.

### Increased endothelial cell proliferation and loss of LSEC zonation in *Maf*^*LSEC-KO*^ mice

To further analyze the changes in hepatic microvascular endothelial differentiation, bulk RNA-seq was performed using LSECs from *Maf*^*LSEC-KO*^ *vs.* control mice isolated by gradient centrifugation and positive selection for CD146 antigen expression (Table S1, Fig. S5A and B). Strictly speaking, these cells also include ECs from large hepatic blood vessels, *e.g*. central veins and portal arteries, but because LSECs represent by far the largest EC population in the liver^13^ and for readability, we refer to these cells as "LSECs".

RNA-seq confirmed the effective knockout of *Maf* in LSECs (Fig. S5C). To identify critical genes that promote the phenotype seen upon *Maf* deficiency in LSECs, we focused on the top differentially expressed genes in *Maf*^*LSEC-KO*^ LSECs.

**Fig. 5:**
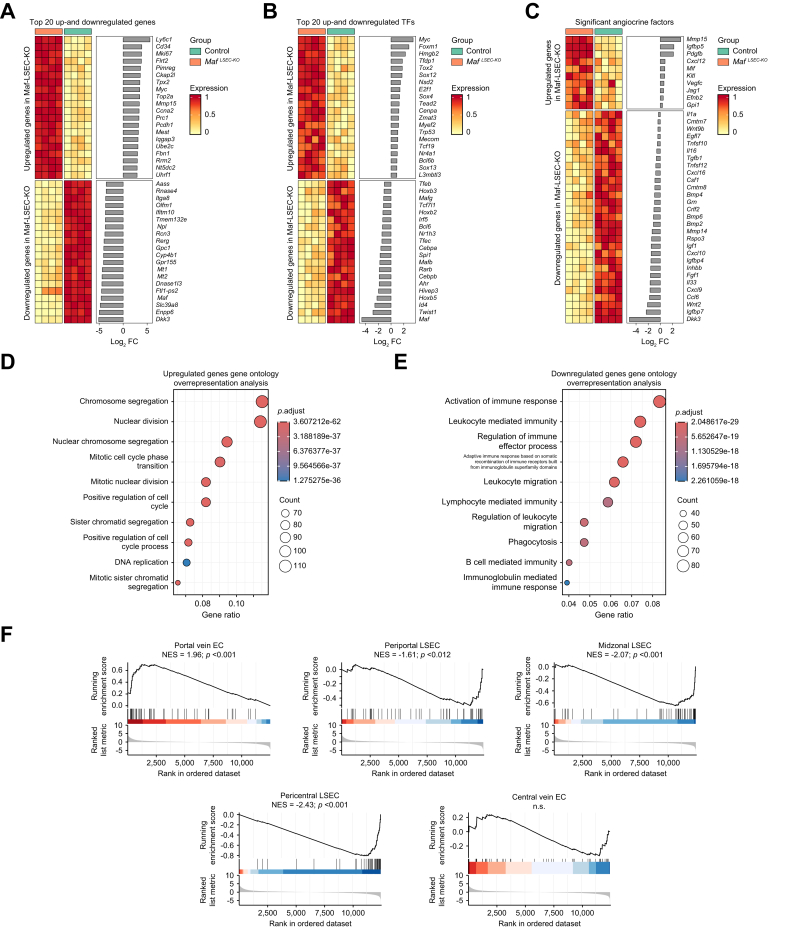
Dysregulation of transcription factors and angiocrine factors with a shift towards portal vein transcripts in *Maf*-deficient LSECs. Heat maps of (A) top significantly up- and downregulated genes, (B) transcription factors, and (C) angiocrine factors in LSECs. Gene ontology overrepresentation analyses of (D) upregulated and (E) downregulated genes. (F) Enrichment plots of portal vein, periportal, midzonal, pericentral, and central vein associated genes. NES, normalized enrichment score (n = 4). LSECs, liver sinusoidal endothelial cells.

Among the most strongly upregulated genes, we identified the continuous endothelium marker gene *Cd34* and the proliferation marker gene *Mki67* (Fig. 5A) confirming our IF data (Figs 2B and 3B). Interestingly, *Ly6c1* was the most strongly upregulated gene, a gene primarily known as a monocyte/macrophage marker. However, LY6C IF confirmed the endothelial expression of LY6C especially in *Maf*^*LSEC-KO*^ livers (Fig. S6A). As *Ly6c1* has been described to be expressed by ECs in various vascular beds, induced endothelial expression of *Ly6c1* in *Maf*^*LSEC-KO*^ livers may be part of the sinusoidal-to-continuous endothelial dedifferentiation program.^14^^,^^15^

Among the transcription factors, *Myc* was the most strongly upregulated in LSECs from *Maf*^*LSEC-KO*^ mice (Figs 5B and S6B). In addition, the transcriptomic data confirmed the upregulation of the profibrotic factors *Igfbpb5* and *Pdgfb* (Fig. 5C). Upregulation of these genes is consistent with LSEC dedifferentiation and is congruent with the increased proliferative and profibrotic capacity of LSECs due to *Maf* deficiency.

Notably, cell adhesion and extracellular matrix-interacting molecule *Flrt2* and *Cxcl12* were identified as novel candidate molecules that may contribute to liver fibrogenesis in *Maf*^*LSEC-KO*^ mice (Fig. 5A,C). Significant upregulation of *Flrt2*, was confirmed by ISH and qPCR (Figs S6C-E and S7A). *Cxcl12* was mostly expressed by cells other than LSECs, but there was no significant difference between the genotypes, albeit a trend towards higher expression in *Maf*^*LSEC-KO*^ livers (Fig. S6D and E).

Interestingly, *Wnt9b*, *Rspo3,* and *Wnt2* were significantly downregulated in our bulk RNA-seq data of *Maf*-deficient LSECs (Fig. 5C), while we did not see significant changes by ISH (Fig. S7B). Similarly, *Bmp2* and *Hgf* were not significantly altered on ISH. However, ISH is not as sensitive as RNA-seq, a difference which may explain why results for these genes in ISH did not confirm differential expression. In addition, *Wnt9b*, *Rspo3,* and *Wnt2* are preferentially expressed by central vein ECs and pericentral LSECs. Preservation of a certain gradient and level of expression of Wnt signaling molecules in LSECs may also explain preserved metabolic liver zonation in *Maf*^*LSEC-KO*^ mice (Fig. 1F,G), especially since *Maf* expression is lower in pericentral LSECs and *Maf* excision in pericentral LSECs is less effective compared to midzonal LSECs (see below).

Gene ontology analysis of transcripts altered in LSECs isolated from *Maf*^*LSEC-KO*^ mice further revealed significant upregulation of genes associated with cell proliferation, including chromosome segregation, nuclear division and mitotic cell cycle transition (Fig. 5D), while the downregulated genes were associated with immunological functions including activation of immune response, leukocyte-mediated immunity, and regulation of immune effector processes (Fig. 5E). The downregulation of immunological gene sets in LSECs from *Maf*^*LSEC-KO*^ mice, even though we observed more monocytes/macrophages in *Maf*^*LSEC-KO*^ liver tissue (Fig. S3B) that could have contaminated our LSEC preparations, could be due to the capillarization of LSECs in our *Maf*^*LSEC-KO*^ mice, since continuous ECs generally express fewer immunological molecules than healthy LSECs.^16^

Enrichment analysis to distinguish tip cells from stalk cells^17^ indicated a slight preponderance of tip cell markers among the genes upregulated in LSECs isolated from *Maf*^*LSEC-KO*^ mice, indicating ongoing angiogenesis (Fig. S8A).

Gene set-enrichment analysis of LSEC marker gene sets revealed an increase in the expression of portal vein endothelial genes in *Maf*^*LSEC-KO*^ LSECs, while specific periportal, midzonal, and pericentral LSEC transcripts were reduced (Fig. 5F). This finding confirms a change in differentiation of LSECs towards the differentiation of continuous ECs accompanied by loss of sinusoidal zonation in *Maf*^*LSEC-KO*^ mice. On the contrary, central vein transcripts were significantly less affected in *Maf*^*LSEC-KO*^ mice (Fig. 5F). In addition, lymphatic EC markers were slightly increased (Fig. S8B).

### Endothelial *Maf* deficiency causes dysregulated chromatin accessibility promoting counter-regulation of genes physiologically expressed or silenced in LSECs

**Fig. 6:**
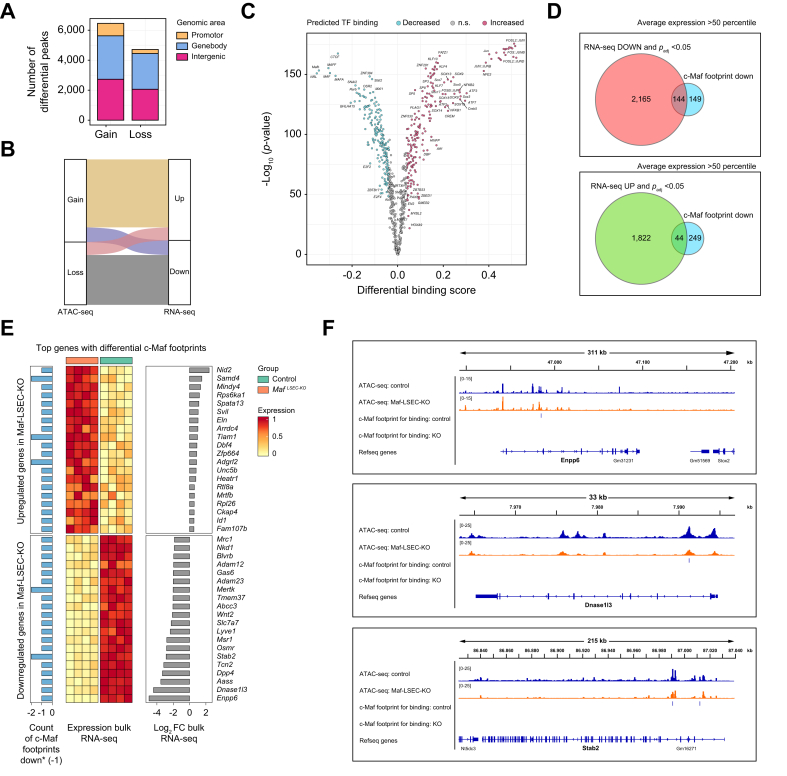
Loss of endothelial *Maf* leads to major alterations in chromatin accessibility. (A) Bar plot of differentially accessible chromatin areas at promotor, genebody, and intergenic regions in LSECs upon *Maf* deficiency. (B) Alluvial plot illustrating concordance of ATAC-seq and RNA-seq data. (C) Volcano plot of TOBIAS footprinting analysis of ATAC-seq data from LSECs. (D) Venn diagrams of genes showing loss of c-Maf footprints and significant dysregulation at the RNA level. (E) Heatmap of the 20 top up- and downregulated genes at the RNA level with loss of c-Maf footprints. (F) Example gene tracks for selected genes *Enpp6*, *Dnase1l3*, and *Stab2* with annotation for ATAC-seq signal and c-Maf footprints per genotype. ATAC-seq, assay for transposase-accessible chromatin with sequencing; LSECs, liver sinusoidal endothelial cells; RNA-seq, RNA sequencing.

To better understand the role of c-Maf in orchestrating LSEC gene expression, we characterized the chromatin landscape of LSECs isolated from control and *Maf*^*LSEC-KO*^ mice by ATAC-seq (assay for transposase-accessible chromatin with sequencing). In agreement with the bulk RNA-seq analyses (Fig. 5), *Maf* deficiency led to broad changes in the chromatin accessibility of LSECs, with over 10,000 differentially accessible regions in LSECs from *Maf*^*LSEC-KO*^ mice (Fig. 6A, Table S2), including 6,454 sites with increased accessibility and 4,713 sites with decreased accessibility. Overall, we observed a high degree of concordance between the changes observed at the chromatin accessibility and gene expression levels (Fig. 6B).

In addition, we performed TOBIAS footprinting analysis^18^ using our ATAC-seq data to identify transcription factors that differentially bind to the genome upon *Maf* deficiency in LSECs. Reassuringly, the TOBIAS results revealed a cluster of MAF transcription factors with markedly reduced activity in *Maf*^*LSEC-KO*^ LSECs, likely reflecting lack of c-Maf. Moreover, this analysis indicated reduced binding by the architectural transcription factor CTCF, whereas transcription factors such as FOS and JUN, which have been reported to form heterodimers with c-Maf,^19^ were more active and showed more genomic binding (Fig. 6C, Table S3).

Next, we focused on differential c-Maf footprints – unbound in the *Maf-*knockout LSECs and bound in controls – to identify direct transcriptional targets of c-Maf. After filtering for highly expressed genes (>50^th^ expression percentile in RNA-seq data), we identified 293 genes that were significantly less bound by c-Maf in the *Maf*-knockout LSECs. These genes were intersected with the significantly differentially expressed genes from our RNA-seq data to identify genes in which c-Maf shows direct transcriptional relevance (Fig. 6D).

A total of 144 genes from the differentially less bound list were also found among the significantly downregulated genes in RNA-seq, while only 44 genes overlapped with the significantly upregulated genes (Fig. 6D, Table S4). Among the genes directly positively regulated by c-Maf, we identified *Enpp6*, *Dnase1l3*, *Aass*, *Stab2*, *Lyve1*, and *Wnt2* (Fig. 6E-F). While the same analysis indicated that c-Maf is a direct negative regulator of *Nid2*, *Samd4*, and *Mindy4* (Fig. 6E).

### Endothelial *Maf* deficiency causes replacement of zonated LSEC subpopulations with capillarized, profibrotic and angiogenic endothelial cell subsets

To investigate whether endothelial *Maf* deficiency alters the composition and zonated gene expression profiles of LSEC subpopulations, we performed scRNA-seq on isolated LSECs from control and *Maf*^*LSEC-KO*^ mice followed by bioinformatic filtering to exclude contaminating ECs from large blood vessels, immune cells, stellate cells, and hepatocytes (*Adgre1*-, *Clec4f*-, *Ptprc*-, *Cd52*-, *Acta1*-, *Arg1*-, *Pecam1*+, *Vwf*-). We merged the filtered single-cell data from the different samples, *i.e. Maf*^*LSEC-KO*^ and controls, to investigate differences between the respective LSEC populations.

**Fig. 7:**
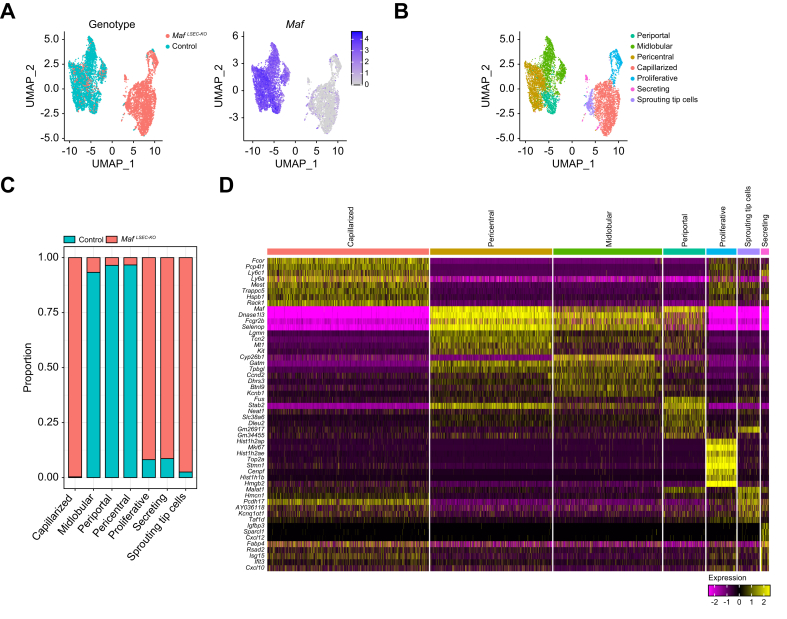

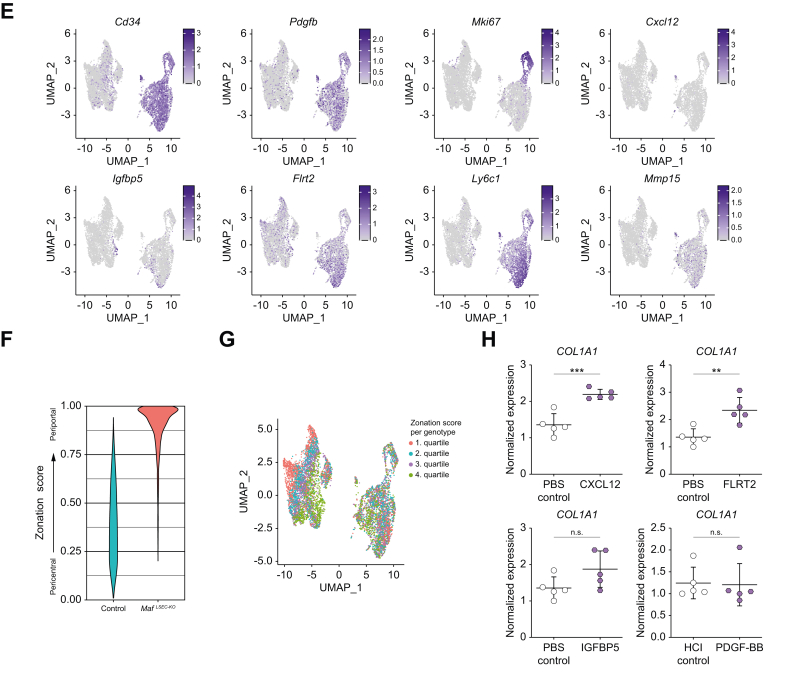
Identification of novel cell clusters in LSECs upon endothelial *Maf* deficiency. (A) Genotype annotation in UMAP plot of scRNA-seq from control and *Maf*^*LSEC-KO*^ LSECs. (B) Annotation for identified clusters in scRNA-seq data. (C) Plot of cell cluster proportions per genotype. (D) Heatmap of identified clusters and their marker genes. (E) Annotation for the genes of interest (*Cd34*, *Pdgfb*, *Mki67*, *Cxcl12, Igfbp5*, *Flrt2, Ly6c1,* and *Mmp15*) in UMAP plot. (F) Violin plot of LSEC zonation scores per genotype (0 corresponds pericentral, 1 corresponds periportal). (G) Annotation for the zonation score quartiles per genotype in UMAP plot. (H) qPCR for *COL1A1* using LX-2 cell RNA after stimulation with CXCL12, FLRT2, IGFBP5 and PDGF-BB (n = 5). (H [CXCL12, FLRT2, IGFBP5]) unpaired *t* test; (H [*PDGF-BB*]) Mann-Whitney *U* test; n.s. *p* >0.05; ∗*∗p <*0.01; ∗∗*∗p <*0.001. LSECs, liver sinusoidal endothelial cells; scRNA-seq, single-cell RNA sequencing; UMAP, uniform manifold approximation and projection.

The uniform manifold approximation and projection plot of the merged data showed an almost complete separation between control and *Maf*^*LSEC-KO*^ LSECs (Fig. 7A). Notably, *Maf*-expressing LSECs from *Maf*^*LSEC-KO*^ mice clustered together with LSECs from control animals (Fig. 7A). Clustering analysis revealed seven distinct clusters: periportal, midlobular, and pericentral subclusters – predominantly found in control LSECs – and capillarized, proliferative, sprouting, and secreting subclusters, which were predominantly observed in *Maf*^*LSEC-KO*^ LSECs (Fig. 7B-D).

The continuous EC genes *Cd34* and *Pdgfb* were almost exclusively expressed by ECs from *Maf*^*LSEC-KO*^ mice and *Cd34+* as well as *Pdgfb+* cells were homogeneously distributed throughout all the *Maf*^*LSEC-KO*^ LSEC subclusters (Figs 7E and S9A), indicating a general change in differentiation from normal LSECs to continuous profibrotic ECs. *Mki67* – already identified by bulk RNA-seq analysis (Fig. 5A) – labelled the proliferative cluster (Figs 7D,E and S9A). Similarly, the secreting subcluster in *Maf*^*LSEC-KO*^ LSECs was labelled by *Sparcl1* (Fig. 7D). Notably, the secreting cluster also showed expression of angiogenic chemokines like *Cxcl12* (Figs 7D,E and S9A). In addition, we analyzed expression of the profibrotic and angiogenic factors *Igfbp5* and *Flrt2* previously identified in our bulk RNA-seq analysis (Fig. 5A,C) and could show that they were preferentially expressed in the capillarized subcluster (Figs 7E and S9A). Using our scRNA-seq data, we again confirmed the endothelial expression of *Ly6c1* and *Mmp15,* which was previously identified by bulk-RNA-seq of LSECs in *Maf*^*LSEC-KO*^ livers (Figs 7E and S9A).

To analyze LSEC zonation in control and *Maf*^*LSEC-KO*^ mice, we developed a zonation score based on the expression ratio of known periportal and pericentral LSEC genes as described in detail in supplementary materials and methods. This analysis demonstrated disruption of physiological LSEC zonation in *Maf*^*LSEC-KO*^ mice (Fig. 7F,G) confirming the shift in endothelial phenotype towards periportal LSECs seen in our bulk RNA-seq data (Fig. 5F).

In addition, scRNA-seq data from LSECs confirmed the previous results that *Col4a1* is produced by ECs, whereas *Col1a1* and *Col3a1* must originate from other hepatic cells, most likely HSCs (Fig. S10A and B).

We also used our scRNA-seq data to investigate the zonated expression of Wnt factors. Interestingly, the Wnt factors *Wnt2*, *Wnt9b,* and *Rspo3* were highly downregulated in *Maf*^*LSEC-KO*^ LSECs, while a gradient of low expression levels was conserved, with higher expression in pericentral areas compared to periportal areas (Fig. S10C). This may be due to lower *Maf* expression in pericentral LSECs, and the likely reduced efficiency of *Maf* excision in these cells owing to lower *Clec4g* expression compared to midlobular LSECs (Fig. S10C). This may explain why we see conserved metabolic zonation in our *Maf*^*LSEC-KO*^ model (Fig. 1F,G), despite the significant loss of Wnt factors in our sequencing data from isolated LSECs.

### Profibrotic factors FLRT2 and CXCL12 activate HSCs *in vitro*

To investigate the functional role of the identified angiocrine factors *Pdgfb*, *Igfbp5*, *Flrt2* and *Cxcl12*, we stimulated the human HSC line LX-2 with the respective angiocrine factors *in vitro*. Notably, FLRT2 and CXCL12, but not IGFBP5 and PDGF-BB, significantly induced *COL1A1* expression in LX-2 cells, indicating HSC activation (Fig. 7H). These results highlight the profibrotic role of angiocrine factors FLRT2 and CXCL12 in liver fibrogenesis.

### *MAF* expression is significantly reduced in ECs of human cirrhotic liver

**Fig. 8:**
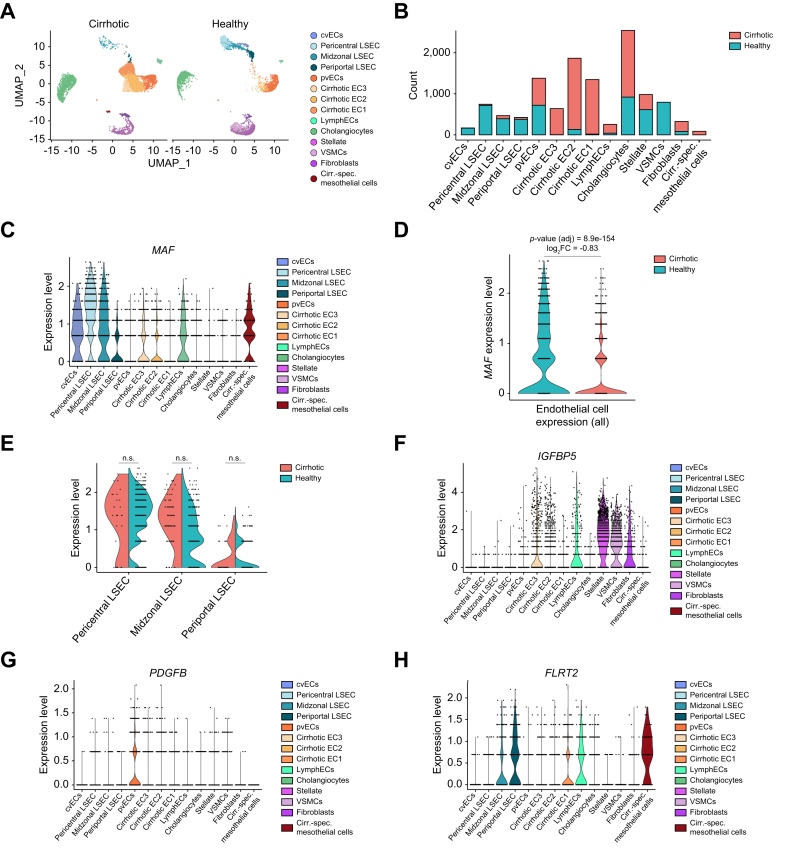
Downregulation of endothelial *MAF* in cirrhotic human livers. (A) UMAP plot of endothelial cells from human cirrhotic and control livers. (B) Bar plot of cell counts mapped to the identified clusters. (C) Violin plot of *MAF* expression per cell cluster. (D) Violin plot of *MAF* expression per phenotype. (E) Violin plot for pericentral, midzonal, and periportal LSECs per phenotype. Violin plots of (F) *IGFBP5*, (G) *PDGFB*, and (H) *FLRT2* expression per cell cluster. cvECs, central vein endothelial cells; LSECs, liver sinusoidal endothelial cells; pvECs, portal vein endothelial cells; UMAP, uniform manifold approximation and projection; VSMCs, vascular smooth muscle cells.

Transcription factors other than c-Maf that determine LSEC differentiation, such as *GATA4* or *ERG*, are known to be downregulated in human patients with liver fibrosis and cirrhosis.^8^^,^^10^ To elucidate whether the expression of transcription factor *MAF* is also altered in human cirrhosis, we analyzed published scRNA-seq data from healthy and cirrhotic human liver samples.^20^ Raw count data was re-analyzed in house (see Methods) to prioritize the resolution of endothelial and zone-specific LSEC clusters. The data was filtered for non-parenchymal cells, enabling the identification of clusters corresponding to distinct hepatic endothelial subpopulations, including pericentral, midzonal and periportal LSECs, as well as lymphatic, central vein and portal vein ECs (Fig. 8A). Consistent with the original publication,^20^ we detected three clusters consisting of cirrhosis-specific ECs, while clusters corresponding to all LSEC zones and central vein ECs were depleted in cirrhotic livers (Fig. 8A). Portal vein ECs retained roughly equal contribution in cells from healthy and cirrhotic livers, suggesting that the loss of characteristic gene expression in cirrhotic ECs is specific to the sinusoidal and central zones (Fig. 8B).

*MAF* expression was almost exclusively seen in healthy LSECs and to a lesser extent in central vein ECs, lymphatic ECs and a small cluster of cirrhosis-specific mesothelial cells (Fig. 8C). Notably, at a global level, *MAF* expression was significantly reduced in ECs from donors with cirrhosis (Fig. 8D), while it was preserved in the few LSECs that retained a pericentral, midzonal, or periportal expression profile (Fig. 8E). We further explored the expression of profibrotic and angiogenic factors that our analyses have implicated as mediators of the role of c-Maf in mouse LSECs (Fig. 5A, 5C). While *IGFBP5* was markedly elevated in cirrhotic ECs compared to LSECs (adjusted *p* value = 3.93e-56, log_2_ fold change = 1.62) (Fig. 8F), the expression of *PDGFB* and *FLRT2* was similar between healthy and cirrhotic ECs (Fig. 8G,H). These results show that the loss of endothelial *MAF* expression in human liver cirrhosis partially parallels the findings in *Maf*^*LSEC-KO*^ mice.

## Discussion

In this study, we show that the transcription factor c-Maf, expressed by normal LSECs, helps protect the liver against MASH-like perisinusoidal liver fibrosis by maintaining LSEC identity. c-Maf-dependent LSEC identity encompasses their zonation along the hepatic sinusoids, from pericentral to periportal regions. The effects of c-Maf in LSECs are mediated by widespread changes in chromatin accessibility, as evidenced by the high number of differentially accessible sites (over 10K). The direct and indirect effects of c-Maf in LSECs, as deduced from TOBIAS footprinting predictions, provide mechanistic insight into how dedifferentiation of LSECs and subsequent functional changes in the hepatic vascular niche may cause MASH-like perisinusoidal liver fibrosis.

Notably, hepatic endothelial c-Maf deficiency allowed for expression of continuous EC genes in liver sinusoids at the expense of sinusoidal EC-specific differentiation and was accompanied by disruption of endothelial zonation along the liver sinusoids. The known profibrotic angiocrine stellate cell activators *Pdgfb* and *Igfbp5* were increased in *c-Maf*-deficient LSECs. *Pdgfb* overexpression alone has been reported to induce liver fibrosis,^21^ while IGFBP5 is known to promote the survival of activated HSCs and myofibroblasts and to increase the expression of profibrotic genes, thereby contributing to liver fibrosis.^22^

Furthermore, we identified *Flrt2* and *Cxcl12* as potential drivers of sinusoidal capillarization and we could show *in vitro* that FLRT2 and CXCL12 contribute to HSC activation by inducing *COL1A1* expression. FLRT2 binds to latrophilin-2 thereby promoting tight junction assembly accompanied by reduced vascular permeability.^23^ In colorectal cancer, FLRT2 formed noncanonical inter-endothelial adhesions that safeguarded against oxidative stress through homophilic binding.^24^ Moreover, FLRT2 has been shown to prevent premature senescence and vascular ageing in endothelial cells upon exposure to risk factors for vascular diseases,^25^ indicating that expression of *Flrt2* may be part of a reactive program to protect the hepatic vasculature from hepatic injury and/or promote healing – a process that requires activation of HSCs and induction of fibrosis. *Cxcl12* is a known proangiogenic chemokine^26^ that is primarily expressed by hepatocytes and ECs.^27^ Therefore, the role of endothelial *Cxcl12* in liver fibrogenesis requires further investigation.

Using scRNA-seq analysis, we discovered that the complete population of LSECs in *Maf*^*LSEC-KO*^ mice underwent a transcriptomic switch in identity towards continuous EC differentiation characterized by homogeneously distributed *Cd34* and *Pdgfb* expression. Among these dedifferentiated LSECs, four distinctive subpopulations were identified, *i.e.* capillary, proliferative, sprouting and secretory clusters. While the secretory cluster may drive the scarring reaction to liver injury via expression of profibrotic angiokines, the proliferative and sprouting clusters may promote the angiogenic part of the reactive response. In line with the emergence of the proliferative and sprouting LSEC subpopulations in *Maf*^*LSEC-KO*^ mice, we identified *Myc* as the most strongly upregulated transcription factor in LSECs in *Maf*^*LSEC-KO*^ mice. MYC is known to be essential for vasculogenesis and angiogenesis^28^ and thus may mediate development of the proliferative and sprouting LSEC subpopulations in *Maf*^*LSEC-KO*^ mice. Furthermore, our data indicate that c-Maf is an important endothelial transcription factor supporting LSEC differentiation^29^ that represses *Myc,* and its vast dependent transcriptional machinery, to keep LSECs in a resting state that supports liver homeostasis.

Mechanistically, we show that the transcription factor c-Maf acts at the epigenetic level, as c-Maf deficiency resulted in remarkable changes to the chromatin accessibility landscape. Our transcription factor footprint analyses provide insight into the complex transcriptomic network of LSECs and reveal that other transcription factors, such as FOS and JUN, become more active upon *Maf* deficiency. As c-Maf forms heterodimers with FOS/JUN,^19^ loss of c-Maf in LSECs might cause a higher rate of FOS-JUN heterodimerization as reflected in the TOBIAS footprinting results. Furthermore, combined genetic deletion of transcription factors *Erg* and *Fli1* in organotypic ECs also causes upregulation of FOS and JUN.^30^

Therefore, we hypothesize that upregulation of FOS/JUN may be part of a default program activated in response to loss of identity in ECs in different vascular beds. Functionally, FOS is a well-known protooncogene that can dimerize with JUN family proteins to form the AP-1 transcription factor complex.^31^ FOS regulates fundamental cellular processes, including cell proliferation and differentiation, and may thus act collaboratively with MYC to support angiogenesis by FOS-dependent upregulation of proangiogenic chemokines.^32^

Our findings pave the way for the development of novel treatment strategies against liver fibrosis. Targeting the profibrotic signaling pathways of the angiocrine factors identified in this study may offer multiple therapeutic opportunities. For PDGF-BB inhibition, several small molecule tyrosine kinase inhibitors are available including imatinib, sorafenib, nilotinib, and sunitinib that suppress proliferative and fibrogenic properties of activated HSCs.^33^ IGFBP5 inhibition could be achieved with the IGF-1R inhibitor linsitinib,^34^ which is not currently approved for any clinical indication. In addition, erdafitinib^35^ and plerixafor^36^ are experimental candidates to inhibit FLRT2 and CXCL12 signaling, respectively. On the other hand, *Maf* could be targeted to increase its expression in LSECs either using lipid nanoparticles^37^ or lentiviral vectors.^38^

Taken together, this study provides important insights into the role of the endothelial transcription factor c-Maf in maintaining normal liver function and protecting against hepatic fibrogenesis, and may open new avenues for developing angiotargeted strategies for liver repair.

## Abbreviations

CDAA, choline-deficient, L-amino acid-defined; ECs, endothelial cells; HSCs, hepatic stellate cells; IF, immunofluorescence; ISH, *in situ* hybridization; KO, knockout; LSECs, liver sinusoidal endothelial cells; MASH, metabolic dysfunction-associated steatohepatitis; RNA-seq, RNA sequencing; sc, single-cell.

## Financial support

This work was supported by the Deutsche Forschungsgemeinschaft, Germany (DFG, German Research Foundation): RTG/GRK 2099 (project number 259332240); CRC/SFB 1366 (project number 394046768); ICON/EB 187/8-1 (project number 413262200). IC is recipient of a Sir Henry Dale Fellowship jointly funded by the Wellcome Trust and the Royal Society (224662/Z/21/Z). DN is recipient of a PhD studentship by the British Heart Foundation (FS/4yPhD/F/20/34128). RM is the recipient of a Fonds de la Recherche Scientifique – (FNRS-F.S.R.) fellowship (1.B306.22). AR is recipient of British Heart Foundation funding (RG/17/4/32662).

## Authors’ contributions

Conceptualization, C.D.S, P.-S.K., S.G.; Investigation, C.D.S., M.W., T.S., M.S., S.K.-Z., J.H., J.C., L.K., H.M., D.N., R.M., C.S., M.N., K.R.; Data Curation, C.D.S., M.W., J.C., L.K., H.M., R.M., C.S.; Writing – Original Draft, C.D.S., P.-S.K., S.G.; Writing – Review & Editing, C.D.S., M.W., T.S., M.S., S.K.-Z., J.H., J.C., L.K., M.N., K.R., G.D., H.M., D.N., I.C., A.R., R.M., C.S., P.-S.K., S.G.; Visualization, C.D.S., M.W., T.S., M.S., J.H., J.C., L.K., H.M., D.N., R.M, M.N., K.R.; Supervision, C.D.S., P.-S.K., S.G.; Funding Acquisition, C.D.S, M.W., P.-S.K., S.G.

## Conflict of interest

The authors declare no competing interests.

Please refer to the accompanying ICMJE disclosure forms for further details.
