# Supplementary material for: Endothelial c-Maf prevents MASLD-like liver fibrosis by regulating chromatin accessibility to suppress pathogenic microvascular cell subsets
Source: JHEP Rep. 2025 Jun 6;7(9):101475. doi: 10.1016/j.jhepr.2025.101475 (PMC12341620; doi:10.1016/j.jhepr.2025.101475)
Supplement: Multimedia component 1 [file mmc1.pdf]

Endothelial c-Maf prevents MASLD-like liver fibrosis by regulating chromatin accessibility to suppress pathogenic microvascular cell subsets

Manuel Winkler, Theresa Staniczek, Maximilian Suhayda, Sina Wietje Kürschner-Zacharias, Johannes Hoffmann, Julio Cordero, Linda Kraske, Hannah Maude, Dorka Nagy, Rita Manco, Carsten Sticht, Michelle Neßling, Karsten Richter, Gergana Dobreva, Anna Maria Randi, Inês Cebola, Kai Schledzewski, Philipp-Sebastian Reiners-Koch, Sergij Goerdt, and Christian David Schmid

Table of contents

Supplementary Material and Methods 2
Fig. S1 15
Fig. S2 16
Fig. S3 17
Fig. S4 19
Fig. S5 20
Fig. S6 21
Fig. S7 22
Fig. S8 23
Fig. S9 24
Fig. S10 25
Table S1 26
Table S2 26
Table S3 26
Table S4 26
Supplementary References 27

Supplementary Material and Methods

Tissue preparation

Isofluran (Baxter) anesthetized mice were sacrificed by cervical dislocation. The liver was removed and weighed. Sectioned organs were fixed in phosphate-buffered 4 % formaldehyde solution (P087, Carl Roth) at room temperature for one to five days, followed by paraffin embedding according to standard protocols. Liver tissue pieces were also snap-frozen in liquid nitrogen.

Formalin-fixed paraffin-embedded (FFPE) tissue sections (3 µm) were deparaffinized and rehydrated according to standard protocols. Sections were stained with hematoxylin & eosin (H&E), Picrosirius red (PSR), and Prussian Blue according to standard protocols. For Oil Red O staining, fresh frozen cryosections were processed according to the manufacturer's standard protocols and counterstained with haematoxylin solution.

Blood collection

For blood collection, mice were deeply anesthetized and sacrificed subsequently. Blood samples were taken from the retrobulbar venous plexus during day cycle in lithium heparin tubes (Microvette 500 LH, 20.1345.100, Sarstedt). Plasma was separated using centrifugation at 7000 x g for 7 min and analyzed for standard plasma values in a Cobas c311 analyzer (Roche Diagnostics).

Immunofluorescence

3 µm paraffin sections were de-paraffinized, rehydrated, and stained according to standard protocols. Heat induced epitope retrieval (HIER) citrate buffer pH 6.0 (ZUC028-500, Zytomed Sytems) was used for antigen retrieval at 95 °C for 45 min.

After air-drying, cryosections (8 µm) were fixed for 10 min with phosphate-buffered 4 % paraformaldehyde (PFA) (0335, Carl Roth) and blocked with 5 % normal donkey serum (017-000-121, Dianova, Hamburg, Germany) for 30 min.

Antibodies were diluted in Dako antibody diluent (S202230-2, Agilent Technologies). Primary antibodies were incubated overnight at 4 °C. Subsequently, sections were washed three times with phosphate-buffered saline (PBS) (A0964.9050, VWR International) and incubated with fluorophore-conjugated secondary antibodies for one hour at room temperature followed by another washing step with PBS. Finally, sections were mounted with Dako fluorescence mounting medium (S302380-2, Agilent Technologies), dried for one day at room temperature, and stored at 4 °C. Antibodies used for immunofluorescence are listed in CTAT file.

Immunohistochemistry

Sections of 3 µm thickness were prepared from paraffin-embedded tissue, following standard protocols for deparaffinisation and rehydration. Antigen retrieval was performed at pH6 (citrate buffer) for 45 minutes. The sections were then blocked with Dako Real Peroxidase Blocking Solution (Agilent Technologies, S2023) for 10 minutes. The primary antibody was diluted in Dako Antibody Diluent (Agilent Technologies, S202230-2) and incubated overnight at 4 °C in a humid chamber. Following this, the sections were washed three times with PBS and incubated with HRP-conjugated antibodies for 1 hour at room temperature. Following a further three washes with PBS, the sections were then incubated with Dako Liquid DAB + Substrate (Agilent Technologies, K3468) for a period of 8 minutes. They were then counterstained with haematoxylin solution, Gill No. 1 (Sigma-Aldrich, GHS132-1L) for a further 4 minutes, after which they were mounted with Dako aqueous mounting medium (Agilent Technologies, S3025).

Microscopy

Images were acquired using an Eclipse Ni-E motorized upright microscope equipped with CFI Plan Apochromat Lambda series objective lenses (4x, 10x, 20x, 40x, 60x), an Intensilight Epifluorescence Illuminator, a DS-Ri2 high-definition color camera, and a DS-Qi2 high-definition monochrome camera controlled by NIS-Elements AR 5.6.30 software (Nikon Instruments). To capture the total thickness of the specimen, fluorescence microscopy images were acquired as series of z-axis images. Acquired

images were background corrected, deconvoluted, and focused using NIS-Elements AR 5.6.30 and Fiji ImageJ 2.0.0-rc69/1.53c [1,2].

Representative areas per sample were selected for quantification of immunofluorescence or in situ hybridization images. The images were quantified using ImageJ software with a pre-programmed threshold appropriate for the analysis. The area was then measured using the 'Measure' tool implemented in ImageJ.

qPCR

RNA Isolation

Liver tissue was homogenized using the Precellys® Tissue Homogenizer (Bertin Technologies, France) and the Precellys® Lysing Kit CKMix (2 mL tubes with ceramic beads) at 5000 rpm for 2 × 20 seconds. Total RNA from liver tissue and hepatic endothelial cells was isolated using innuPREP RNA Mini Kit 2.0 (845-KS-2040250, Analytik Jena) followed by DNA digestion using the TURBO DNA-free Kit (AM1907, Thermo Fisher Scientific) according to the manufacturer's protocols. RNA concentration and quality were measured using a NanoPhotometer NP80 (Implen) and a 2100 Bioanalyzer (Agilent Technologies).

Reverse Transcription

Reverse transcription was performed usingMaxima Reverse Transcriptase (EP0752, Thermo Fisher Scientific) and Oligo(dT)18 primers (SO131, Thermo Fisher Scientific) according to the manufacturer’s instructions.

qPCR settings and analysis

innuMIX qPCR SyGreen Sensitive (845-AS-1310200, Analytik Jena, Jena, Germany) was used on a qTOWER 3 G touch thermal cycler (Analytik Jena) for quantitative PCR (qPCR). qPCR primers were designed with NCBIs PrimerBLAST (https://www.ncbi.nlm.nih.gov/tools/primer-blast/). For mRNA specificity, qPCR primers were designed to span an exon-exon junction where possible. Primers were tested with no template controls, original RNA, and melt curve analysis. Primer sequences are listed in

the CTAT file. qPCR output files were analyzed in qPCRsoft 4.0.8.0 (Analytik Jena). Considering the amplification efficiencies determined from the standard curves, normalized expression values were calculated using the Pfaffl method. For normalization, reference genes *Gak*, *Mrpl46*, and *Srp72* [3] were used.

Tissue Collagen Assay

Liver tissue was analyzed for collagen content using the Sensitive Tissue Collagen Assay (QZBtiscol1, QuickZyme) according to the manufacturer's instructions. Optical densities were detected with an Infinite 200 plate reader (Tecan Group) set to 570 nm.

Hepatic triglyceride concentration assay

To analyze the hepatic triglyceride concentration, 100 mg snap frozen liver tissue was homogenized in 5 % NP-40 solution (74385, Merck) by two cycles of heating to 80–100°C for 5 minutes using a shaking dry incubator (ThermoMixer C, Eppendorf, Hamburg, Germany) and cooling to room temperature. The supernatant was used to determine the triglyceride concentration after centrifugation at top speed for two minutes. Triglyceride concentration was analyzed using Triglyceride Assay Kit – Quantification (ab65336, Abcam) based on the manufacturer's protocol.

Transmission electron microscopy (TEM)

Dissected liver lobes of six months old mice were immersed in freshly prepared aldehyde fixative (4% formaldehyde, 2 % glutaraldehyde, 1 mM MgCl2 and 1 mM CaCl2 buffered to pH 7.2 in 100 mM Na-cacodylate). Vibratome sections (200 µm) of liver lobes were postfixed with1 % osmium tetroxide, dehydrated with ethanol and embedded in epoxy resin. The ultra-thin sections (60 nm) contrast-stained with uranyl and lead were viewed in a Zeiss EM 910 at 80 kV (Carl Zeiss, Oberkochen, Germany). Micrographs were taken with a digital CCD-Camera (TRS, Moorenweiss, Germany).

In-situ-hybridization

For RNA in situ hybridization (ISH), RNAscope 2.5 HD Red (322350, Advanced Cell Diagnostics) on FFPE tissue sections (3 µm) according to the manufacturer's protocols was used. ISH probes are listed in CTAT file.

RNA fluorescence in situ hybridization (FISH)

FFPE tissue sections (3 µm) were used for RNA fluorescence in situ hybridization (FISH) and processed according to the manufacturer's protocols. The RNAscope 2.5 HD Duplex kit (Advanced Cell Diagnostics, 322,430) was used. FISH probes are listed in CTAT file.

MASH-induced liver fibrosis mouse model

To induce MASH-induced liver fibrosis 11-week-old female *Maf*$^{LSEC\text{-}KO}$ and littermate controls were fed choline-deficient, L-amino acid-defined (CDAA) diet (E15666-94, Ssniff) for ten weeks. The body weight was monitored during the experiment and blood samples were taken at the end of the experiment prior scarification of the mice.

Isolation of hepatic endothelial cells

Isolation of hepatic endothelial cells was performed as described before [4]. Mice were deeply anesthetized with Isolfluran (Baxter) and sacrificed by cervical dislocation. Livers were perfused *in situ* via the portal vein with a 0.05 % collagenase/amino acid/saccharide solution (C2674, Sigma-Aldrich). Livers were dissected and mechanically disrupted. Liver tissue from three mice was pooled and digested at 37 °C in collagenase/Gey's balanced salt solution (G9779, Sigma-Aldrich). After removing hepatocytes by low speed centrifugation, the cells were filtered through a 250 µm mesh followed by a 100 µm cell strainer. Non-parenchymal cells were separated by a 19.3 % Nycodenz (1002424, Axis-Shield, Alere Technologies) gradient. Subsequently, magnetic-activated cell sorting (MACS) was used with anti-CD146 MicroBeads (ME-9F1, 130-092-007, Miltenyi Biotech) to purify endothelial cells. The purity of hepatic endothelial cells was analyzed by fluorescence activated cell

sorting (FACS) using a BD FACSCanto II (BD Biosciences, Franklin Lakes, NJ, USA). After gating on live singlet cells, the purity of hepatic endothelial cells was indicated by LYVE1+ and/or CD31+ cells. Positivity for LYVE-1 and/or CD31 of hepatic endothelial cells from controls was 95 %, for *Maf*$^{\text{LSEC-KO}}$ was 86 % (Fig. S6A). Macrophage contamination was assessed by CD11b positivity. Positivity of hepatic endothelial cells for CD11b was 2 % for controls and 4 % for *Maf*$^{\textit{LSEC-KO}}$ (Fig. S5A).

Bulk RNA-seq of mouse hepatic endothelial cells

Library preparation and sequencing

RNA of isolated hepatic endothelial cells was conducted as described above. Library preparation and sequencing was conducted by BGI Tech Solutions (Hong Kong) using the BGISEQ-500 platform and paired-end 100-bp read length. Raw reads were filtered using the SOAPnuke software to obtain clean reads (filter parameters: -n 0.03 -l 20 -q 0.4 -A 0.28). At least 20×10^6 clean reads per sample were obtained.

Data analysis

RNA-seq data processing was performed with R (version 3.6.3) and bioconductor (version 3.9) using the NGS analysis package systempipeR [5] in Rstudio (version 1.1.463). Quality control of clean sequencing reads was performed using FastQC (Babraham Bioinformatics). Low-quality reads were removed using trim_galore (version 0.6.4). The resulting reads were aligned to the mouse genome version GRCm38.p6 from GeneCode and counted using kallisto version 0.46.1 [6]. The count data was transformed to log2-counts per million (logCPM) using the voom-function from the limma package [7]. Differential expression analysis was performed using the limma package in R. A false positive rate of $\alpha = 0.05$ with FDR correction was taken as the level of significance.

Volcano plots and heatmaps were created using ggplot2 package (version 2.2.1) and the complexHeatmap package (version 2.0.0) [8]. For Gene Ontology enrichment analysis and gene set enrichment analysis (GSEA), clusterProfiler was used [9]. Only genes with RNA expression >1cpm

were used in the analysis. For Gene Ontology enrichment analysis, genes were further filtered for log2 fold-change >1 or <-1, respectively. GSEA was performed using key genes of LSEC zonation, based on data from Su et al. [10].

ATAC-seq of mouse hepatic endothelial cells

Library preparation

Hepatic endothelial cells were isolated from three 12 weeks old control and *Maf*$^{LSEC-KO}$ mice using a modified protocol from [11]. Briefly, 50000 freshly-isolated hepatic endothelial cells were incubated in 50 µL cold lysis buffer (10 mM Tris-HCl pH 7.4, 10 mM NaCl, 3 mM MgCl2, 0.1 % Igepal CA-630) for three minutes. The lysis was stopped by adding 1 mL resuspension buffer (10 mM Tris-HCl pH 7.4, 10 mM NaCl, 3 mM MgCl2) and the nuclei were pelleted by 10 min centrifugation (500 x g, 4 °C). Isolated nuclei were incubated in transposition reaction mix containing 2.5 µL Nextera Tagment DNA Enzyme TDE (15027916, Illumina) in 47.5 µL 2x transposition buffer (20 mM Tris-HCl pH 7.6, 10 mM MgCl2, 20% Dimethylformamide) for 30 min at 37 °C. Immediately following the transposition reaction, purification was carried out using ChIP DNA Clean and Concentrator Kit (D5205, Zymo). Sequencing libraries were performed using NEB Next High-Fidelity 2x PCR Master Mix (M0541S, New England Biolabs) and Nextera Index Kit (15055290, Illumina). Magnetic bead purification with two-sided size selection using undiluted Agencourt AMPure XP Beads (A63881, Beckman Coulter) ensured library sizes between 150 and 1000 bp. The quality of the libraries was analyzed by Bioanalyzer High Sensitivity DNA analysis kit (5067-4626, Agilent).

Sequencing

Libraries were mixed in equimolar ratios and sequenced on NextSeq550 platform using v2.5 chemistry (15058251, 15057931, 15057941, 20022408, Illumina Nextseq 500/550).

Data analysis

All raw reads were trimmed using Trimmomatic-0.36 with the parameters (ILLUMINACLIP:2:30:10 LEADING:3 TRAILING:3 SLIDINGWINDOW:4:15 MINLEN:20 CROP:70 HEADCROP:10. The

trimmed reads were mapped to the mouse genome from UCSC version mm10 using Bowtie2 (v2.4.4) (default settings). Mapped reads were converted to from sam to bam by the help of samtools (v1.13). PCR duplicates were removed from the bam files with the help of MarkDuplicates.jar from Picard1.119. Bam files were merged by the help of bamtools (v2.5.1) and from the merged files the bigwig files were created. We used bamCoverage from deptools (v3.5.4) (-bs 20 –smoothLength 40 -p max –normalizeUsing RPKM -e 150). Peak calling was performed using MACS2 (v2.2.9.1). Detected peaks were annotated with annotatePeaks.pl from homer (v4.11) [12]. The peaks overlapping the blacklist from mm10 genome were removed. Peaks from controls and *Maf*$^{LSEC\text{-}KO}$ were merged using bedtools (v2.30.0) merge (-d 100). Quantification of bam files on merged peaks was performed using bedtools (v2.30.0) multicov (defaults settings). Differential binding was performed using DESEQ2 (v1.42.1).

Transcription factor footprint analysis

Transcription factor footprint analysis was performed using TOBIAS (v0.16.1) following the program instructions. The JASPAR 2024 vertebrate database was used as a motif reference. The detected differential footprints were annotated to the mouse genome (mm10) using the ChIPseeker package [13].

ATAC-Seq Data Visualization

The interactive genome browser (IGV) program was used to visualize the normalized bigwig files from the ATAC-seq data, as well as the peak files.

ATAC-seq Code Availability

All the codes used in these manuscripts are available upon request to the corresponding authors.

Single-cell RNA-seq analysis of mouse LSEC

Suspension Preparation

Hepatic endothelial cells from three *Maf*$^{LSEC\text{-}KO}$ and three control mice were isolated and pooled as described above.

Hepatic endothelial cells suspensions were adjusted to a concentration of approximately 1,000 cells/µL in PBS to ensure optimal capture efficiency for 10x Genomics Chromium Next GEM technology.

10x Chromium Platform

Single-cell suspensions were loaded into the 10x Genomics Chromium Controller (10x Genomics) to generate Gel Bead-in-Emulsions (GEMs), following the manufacturer's instructions. A targeted cell recovery of 10,000 cells was used to ensure sufficient capture while minimizing doublet formation.

Inside each GEM, reverse transcription was performed using reagents from the 10x Genomics Chromium Single Cell 3' Reagent Kit v3.1. Barcoded mRNA transcripts were converted into cDNA within the emulsion droplets. Following reverse transcription, cDNA was purified with Silane Dynabeads (Thermo Fisher Scientific), and the cDNA libraries were amplified by polymerase chain reaction (PCR) to generate sufficient material for sequencing.

Amplified cDNA was subjected to fragmentation, end repair, and A-tailing using the Chromium Single Cell 3' Library Construction Kit. Adapter ligation and final amplification were performed to generate sequencing-ready libraries. Sample quality was assessed using an Agilent Bioanalyzer (Agilent Technologies) to ensure library integrity and appropriate fragment sizes.

Sequencing

Final libraries were paired-end sequenced on an Illumina NovaSeq 6000 platform (Illumina) at a targeted depth of 200,000 reads per cell.

Data processing

The raw sequencing data were processed using the 10x Genomics Cell Ranger pipeline (v6.0), which performed demultiplexing, barcode assignment, and alignment to the mouse reference genome (mm10).

On the raw matrix output of Cell Ranger, the empty droplets were identified using low UMI counts (500 UMIs), and Otsu's Method, as described in Ben-Moshe et al. [14]. The mean UMI counts of the empty droplets were then subtracted from each cell. Further analyses were performed using the Seurat

package (version 5.0.1) [15]. Cells with total UMI counts lower than 1500 or total gene counts lower than 750 were removed. Cell clustering was based on PCA dimensionality reduction using 16 or 20 PCs (for the control and *Maf*$^{LSEC\text{-}KO}$ data, respectively) and a resolution value of 0.3. For further analysis, mitochondrial ("^mt-") and ribosomal ("Rp") genes were manually removed from the gene list since they are prone to batch-related expression variability. Additionally, doublets were identified and removed using the DoubleFinder Package (version 2.0.3) [16] . Cell type-specific markers were then utilized to interpret the single-cell clusters: *Adgre1* and *Clec4f* for macrophages, *Ptprc* and *Cd52* for immune cells, *Acta1* for hepatic stellate cells, *Arg1* for hepatocytes, and *Pecam1* for endothelial cells. The clustering was used to subset only the endothelial cells. The last subset was done excluding the cells that expressed *Vwf* to delete the vascular endothelial cells [17]. This processing was done independently for each sequencing run. Finally, the processed control and *Maf*$^{LSEC\text{-}KO}$ Seurat Objects were merged. Data were normalized and scaled using the SCTransform function. Cell clustering was based on PCA dimensionality reduction using 22 PCs and a resolution value of 0.3. The resulting clusters were then manually annotated after extracting cluster markers using the FindAllMarkers Seurat function.

Zonation reconstruction of LSEC

The LSEC zonation was computed by adapting the Method used in Ben-Moshe et al. [14]. Specifically, as starting landmarks well-established pericentral landmark genes were used [18]: *Cdh13*, *Wnt2*, *Rspo3*, and *Wnt9b* for the control cells, while for *Maf*$^{LSEC\text{-}KO}$ only *Cdh13* and *Wnt2* were used, as *Wnt9b* and *Rspo3* were too low expressed. After computing Spearman correlations for each gene with the combined expression level of these pericentral landmark genes, genes with mean expression higher than 1E-5 for the control and 1E-6 for the *Maf*$^{LSEC\text{-}KO}$, correlation pval lower than 0.01 and correlation higher than 0.3 and lower than -0.2 (control) and -0.1 (*Maf*$^{LSEC\text{-}KO}$) were considered as central or portal landmark genes, respectively. Following a two-step normalization process which involved normalizing based on the sum of the total UMI count for each cell and scaling by dividing them by their maximal expression, we calculated the zonation coordinate as the ratio of pericentral landmark gene expression

to the sum of central and pericentral landmark gene expression (pLM/(cLM+pLM)). Finally, cells were equally assigned to four discrete zones – periportal, mid-lobule 1 and 2, and pericentral – based on percentiles. To derive zonation profiles of genes, we computed the mean expression levels and corresponding standard errors for cells grouped by zone.

In vitro stimulation of LX-2 cells

For stimulation of human hepatic stellate cell line LX-2 (RRID:CVCL_5792, SCC064, Sigma-Aldrich) with recombinant proteins, LX-2 cells were seeded at a density of 50.000 cells per well in a 6-well plate. After starving with 0.5 % FCS for 24 h, the cells were stimulated with 100 ng/mL CXCL12 (Bio-Techne), 800 ng/mL FLRT2 (Bio-Techne), 100 ng/mL IGFBP-5 (Bio-Techne) or 20 ng/mL PDGF-BB (Bio-Techne), respectively. PBS was used as control for CXCL12, FLRT2 and IGFBP-5, 4 mM HCl was used as control for PDGF-BB. After 24 h stimulation, the cells were harvested and RNA isolated using innuPREP RNA Mini Kit 2.0 (845-KS-2040250, Analytik Jena) followed by DNA digestion using the TURBO DNA-free Kit (AM1907, Thermo Fisher Scientific) according to the manufacturer's protocols. RNA concentration was measured using a NanoPhotometer NP80 (Implen). The cell line was regularly tested for mycoplasma absence by PCR. The PCR Mycoplasma Detection Kit (Biozol, ABM-G238) was used according to the manufacturer's instructions.

Reverse transcription was performed using Maxima Reverse Transcriptase (EP0752, Thermo Fisher Scientific) and Oligo(dT)18 primers (SO131, Thermo Fisher Scientific) according to the manufacturer’s instructions.

qPCR was performed using innuMIX qPCR SyGreen Sensitive (845-AS-1310200, Analytik Jena, Jena, Germany) on a qTOWER 3 G touch thermal cycler (Analytik Jena). qPCR primers were designed with NCBIs PrimerBLAST (https://www.ncbi.nlm.nih.gov/tools/primer-blast/). For mRNA specificity, qPCR primers were designed to span an exon-exon junction where possible. Primers were tested with no template controls, original RNA, and melt curve analysis. Primer sequences are listed in CTAT file. qPCR output files were analyzed in qPCRsoft 4.0.8.0 (Analytik Jena). Considering the amplification

efficiencies determined from the standard curves, normalized expression values were calculated using the Pfaffl method. For normalization the reference genes *CACTIN* and *HLCS* were used.

Analysis of MAF expression in human liver single-cell RNA-seq data

MAF expression was explored in published single-cell RNA-seq (scRNA-seq) data from human livers with a healthy or cirrhotic phenotype [Ramachandran et al. 2019, https://www.nature.com/articles/s41586-019-1631-3] [19]. Cell count matrices, barcodes and gene labels were downloaded from GEO (GEO accession: GSE136103) for all samples and read into R using the Seurat package (v4.3.0) [https://satijalab.org/seurat/authors - use Hao and Hao et al. Integrated analysis of multimodal single-cell data. Cell (2021) for Seurat v4]. Sample-level processing was carried out using the Seurat SCTransform v2 [20] pipeline along with scDblFinder [21] to remove doublets. Clusters were identified using Seurat FindNeighbors and FindClusters, and low-quality cells were filtered out by removing clusters with a mitochondrial gene in the top five marker genes. Processed samples were integrated using SelectIntegrationFeatures > PrepSCTIntegration > FindIntegrationAnchors > IntegrateData > RunPCA > RunUMAP > FindNeighbors > FindClusters. Cell type labels were predicted via integration with the Liver Cell Atlas [22]. Briefly, cell annotation and count matrices were downloaded from the Liver Cell Atlas (www.livercellatlas.org) and processed with SCTransform v2, RunPCA and RunUMAP. The Seurat commands FindTransferAnchors > TransferData were used to predict cell type labels for the Ramachandran et al. data.

Further analysis was concentrated on NPCs by isolating clusters labelled as stromal cells, fibroblasts, cholangiocytes and endothelial cells. Cells were subset for those with nFeature_RNA > 200 and percent.mt < 15 and the above steps repeated to remove small clusters of contaminating non-NPCs. Using the high-confidence set of NPCs, cell types were first assigned independent for healthy and cirrhotic samples (stage 1) and subsequently for all healthy and cirrhotic NPCs processed together (stage 2), with cell types assigned in stage 1 used to inform the final identity of combined clusters. Cell types were predicted using the label transfer method as above but with the CD45- Liver Cell Atlas,

combined with marker gene expression, namely: *PROX1*, *FLT4*, *PDPN,* and *LYVE1* for lymphatic endothelial cells, LSEC marker genes as published by Nagy et al. [23], stellate cell marker genes from PangloaDB 2020, *VWF*, *CD34*, *ENG*, *ACKR1,* and *PECAM1* for macrovascular ECs, *FCGR2B*, *STAB2*, *LYVE1*, *CD14*, *ICAM1*, *FCN3,* and *FCN2* for pericentral LSECs and *PECAM1*, *F8*, *SPARCL1,* and *CLEC14A* for periportal LSECs. Healthy NPC clusters were labelled as pericentral, midzonal and periportal LSEC, lymphatic EC, central vein EC (cvEC) and portal vein EC (pvEC). Notably, we did not observe clear expression of pericentral LSEC marker genes in the cirrhotic NPCs. Three EC clusters identified in the combined data analysis (stage 2) contained cells almost entirely from cirrhotic samples and were labelled as cirrhosis-specific ECs. Combined cell clusters from stage 2 were used to interrogate differential gene expression using the Seurat command, FindMarkers.

Fig. S1

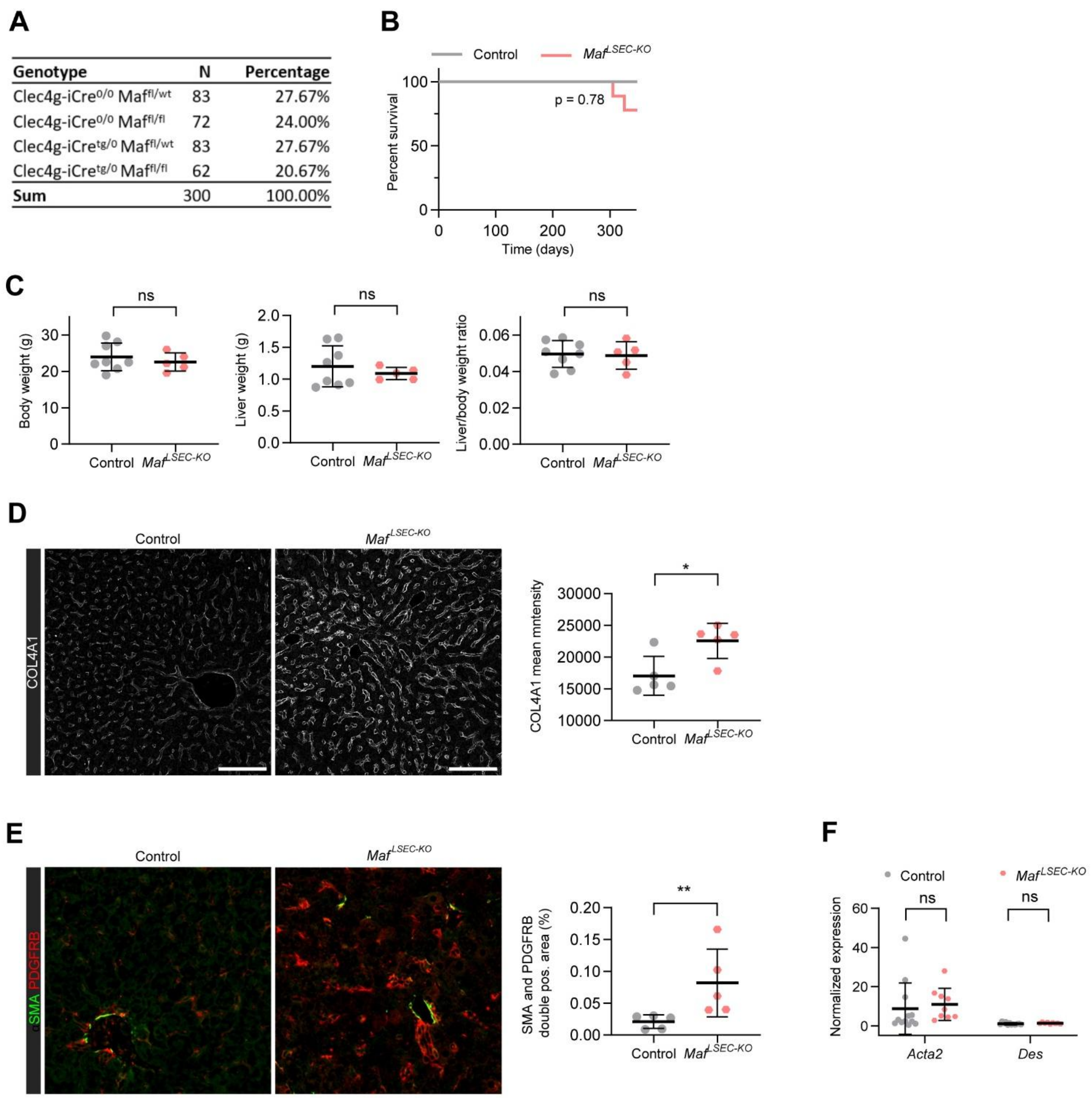

Fig. S1. Basic animal data for *$Maf^{LSEC-KO}$* mice aged three months.

(A) Genotype distribution of *$Maf^{LSEC-KO}$* mice (*$Clec4g$-$icre^{tg/0}$ x $Maf^{fl/fl}$*) (*n* = 300). (B) Kaplan-Meier-curve over a period of 345 days (*n* = 8, 9). (C) Body and liver weight and liver/body weight ratio (*n* = 5, 8). (D) Immunofluorescence staining and intensity quantification of COL4A1 in the liver (*n* = 5). (E) Immunofluorescence staining and quantification of SMA and PDGFRB double positive area in the liver (*n* = 5). (F) Expression levels of *Acta2* and *Des* in livers (*n* = 9, 12). Scale bars: 100 µm. Mean ± SD. (B) Log-rank test; (C-F) Welch's *t* test; n.s. $p \geq .05$; * $p < .05$; ** $p < .01$.

Fig. S2

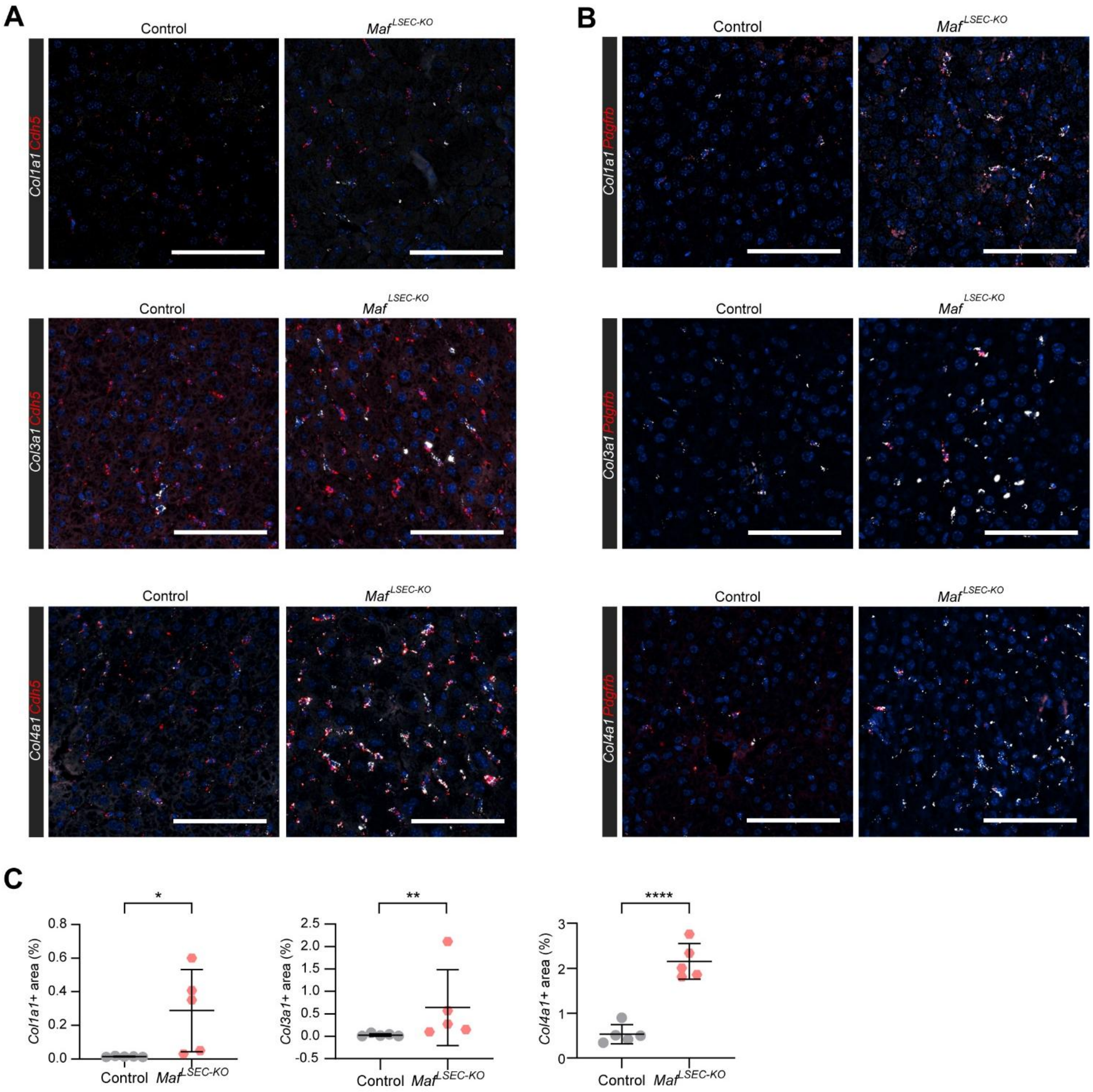

Fig. S2. FISH of collagen genes in *Maf*$^{LSEC\text{-}KO}$ mice.

(A) FISH of liver tissue for *Col1a1*, *Col3a1,* and *Col4a1* using *Cdh5* as an endothelial marker ($n = 5$). (B) FISH of liver tissue for *Col1a1*, *Col3a1,* and *Col4a1* using *Pdgfrb* as a marker for activated HSC ($n = 5$). (C) Quantification of *Col1a1*, *Col3a1,* and *Col4a1* areas in (A) ($n = 5$). (C) Welch's *t* test; * $p < .05$; ** $p < .01$, **** $p < .0001$.

Fig. S3

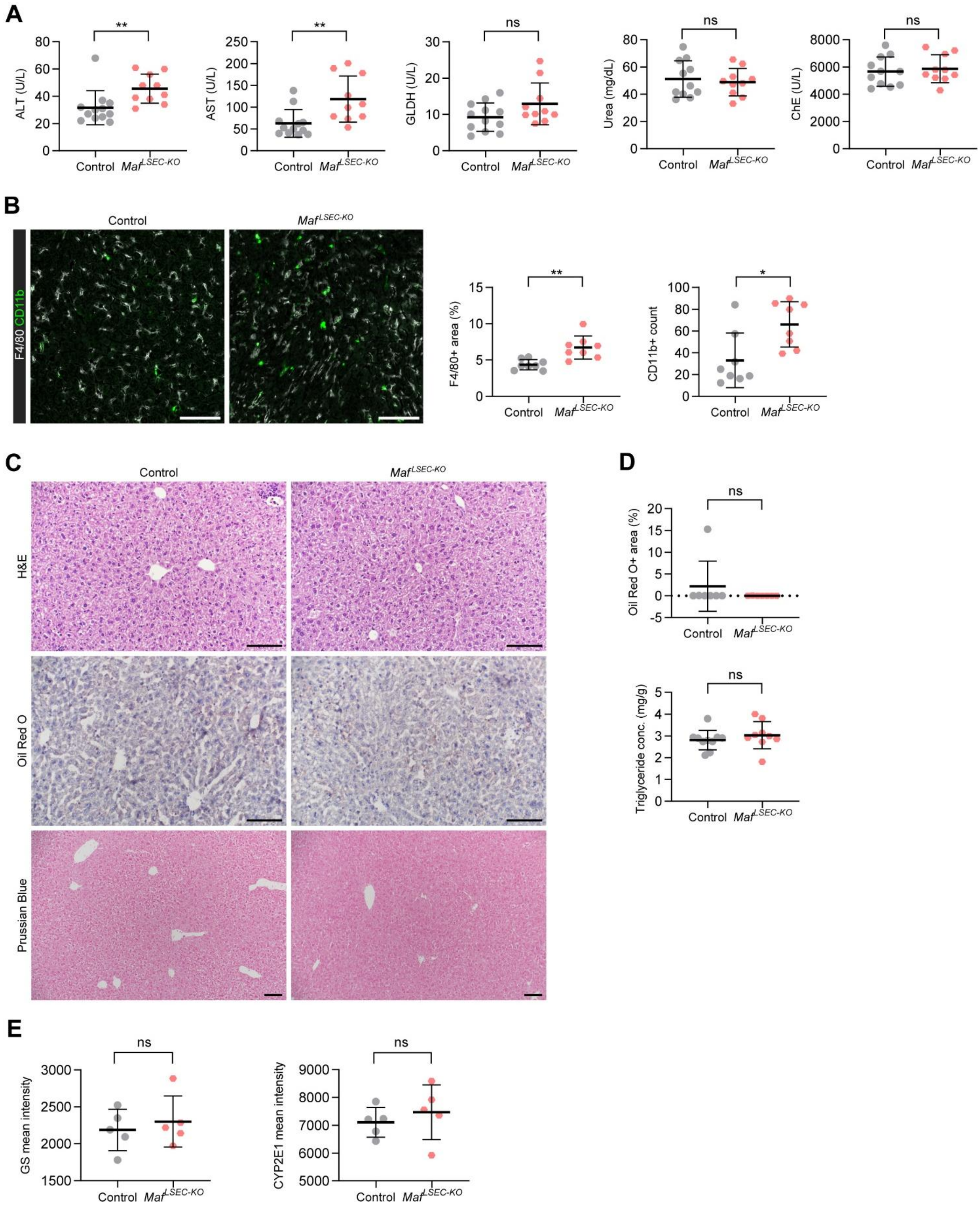

Fig. S3. Plasma values and histology for *Maf*$^{LSEC\text{-}KO}$ mice.

(A) Blood plasma levels of ALT, AST, GLDH, Urea and Cholinesterase (ChE) (*n* = 10, 11, 12). (B) Immunofluorescence staining and quantification of F4/80 and CD11b (*n* = 8). (C) H&E, Oil Red O, and Prussian blue

staining (*n* = 7, 10). (D) Quantification for Oil Red O staining (n = 7) and triglyceride concentration of livers (n = 9, 10). (E) Quantification of GS and CYP2E1 mean intensity (n = 5). Scale bars: 100 µm. Mean ± SD. (A) Mann-Whitney *U* test; (B, D, E) Welch's *t* test; n.s. $p \geq .05$; * $p < .05$; ** $p < .01$.

Fig. S4

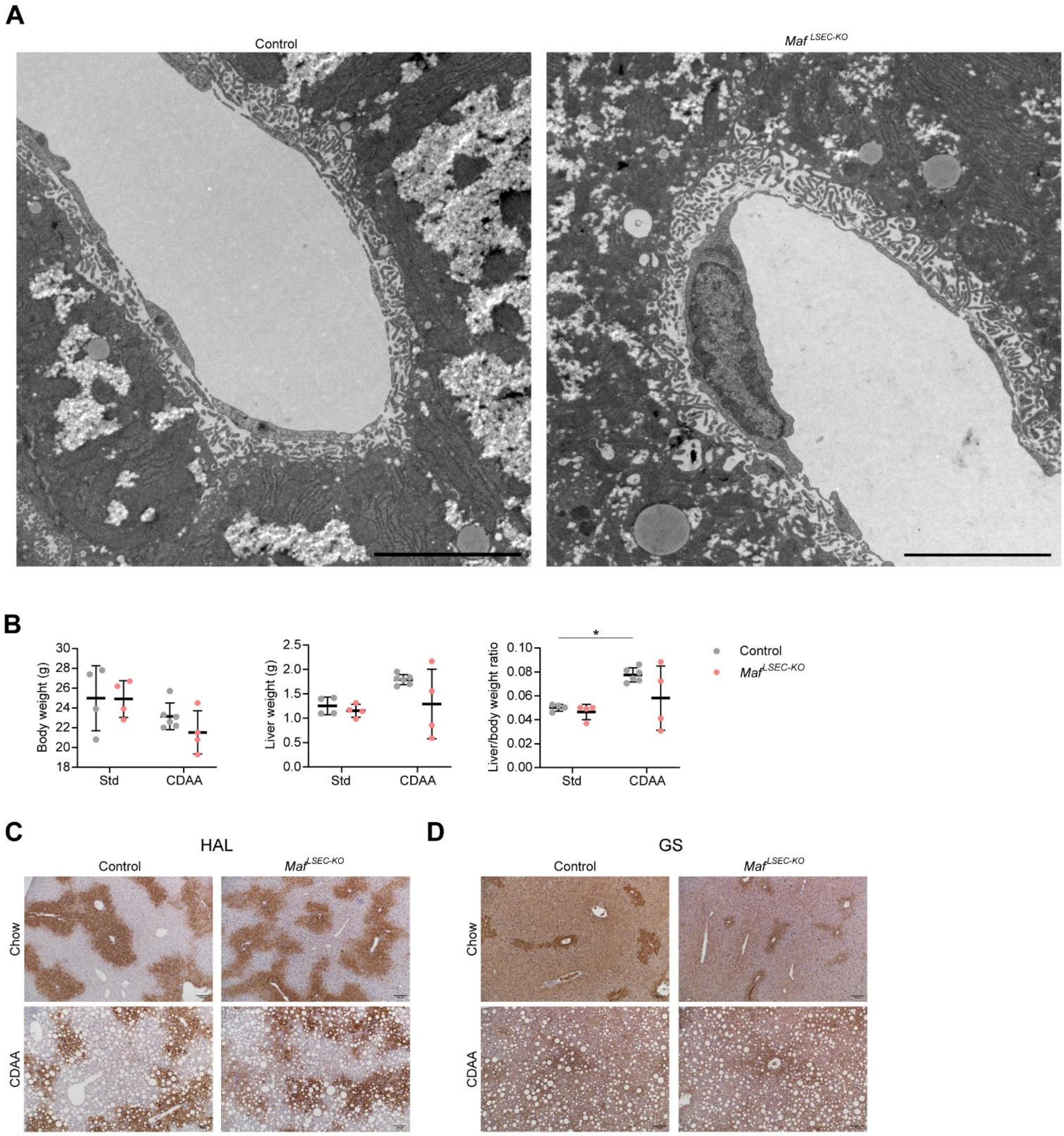

Fig. S4. Transmission Electron Microscopy of Chow fed mice; and basic animal data and metabolic zonation of CDAA diet fed mice.

(A) Transmission Electron Microscopy overview of liver sinus. (B) Body weight, liver weight, and liver/body weight ratio after Chow and CDAA diet (n = 4, 6). IHC staining of (C) HAL and (D) GS in livers after Chow and CDAA diet (n = 4, 6). (A) Scale bars: 5 µm. (C-D) Scale bars: 100 µm. (B) Two-way ANOVA and Tukey's post-hoc test; * $p < .05$.

Fig. S5

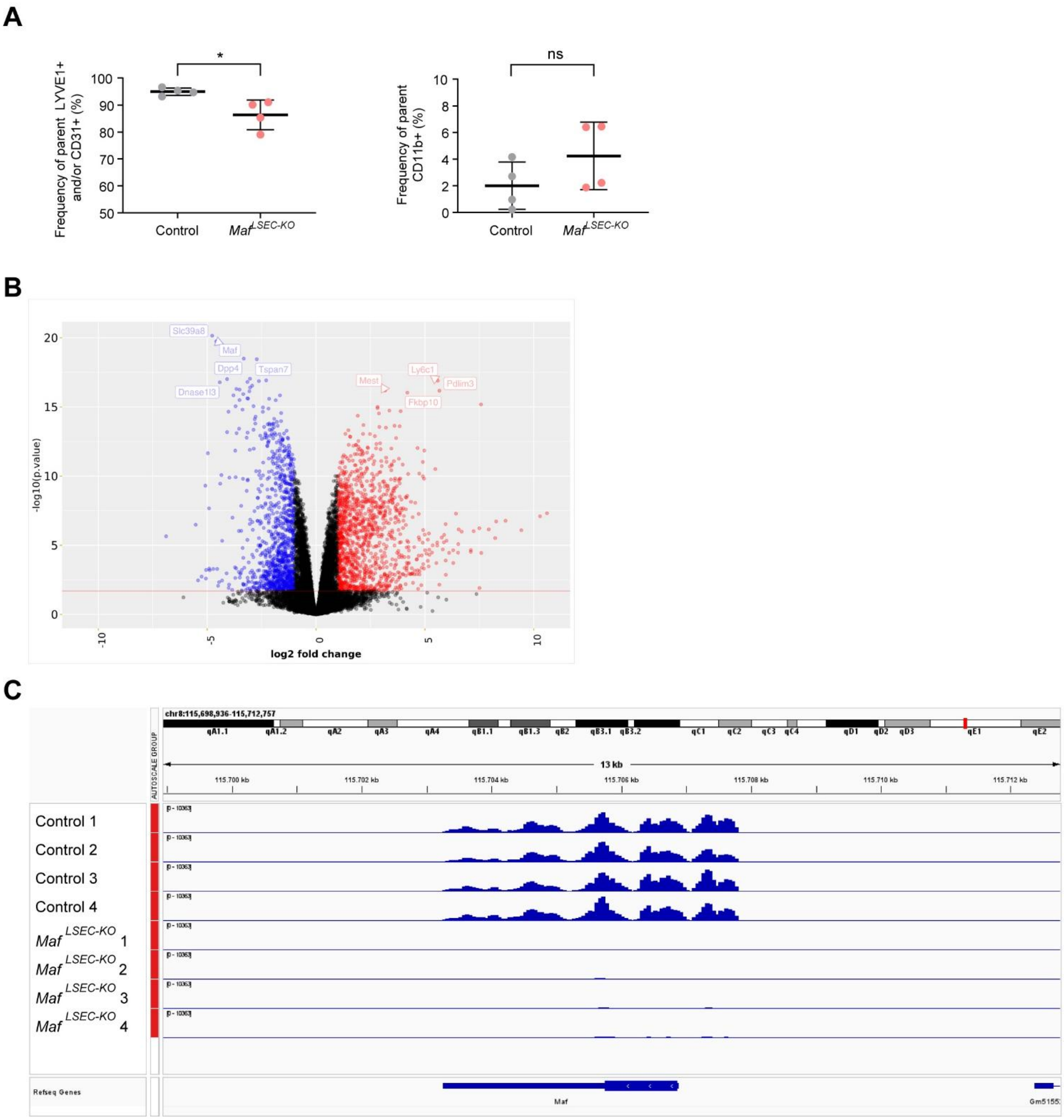

Fig. S5. Bulk RNA-seq analysis of LSEC.

(A) FACS analysis of isolated LSEC using sinusoidal endothelial marker LYVE1, endothelial marker CD31 and myeloid marker CD11b. (B) Volcano plot of fold changes and *p* values of bulk RNA-seq data of LSEC. (C) Gene track for bulk RNA-seq signal from isolated LSEC at the *Maf* locus for controls and $Maf^{LSEC\text{-}KO}$ mice ($n = 4$). Scale bars: 100 µm. Mean ± SD. (A) Welch's *t* test; n.s. $p \geq .05$; * $p < .05$.

Fig. S6

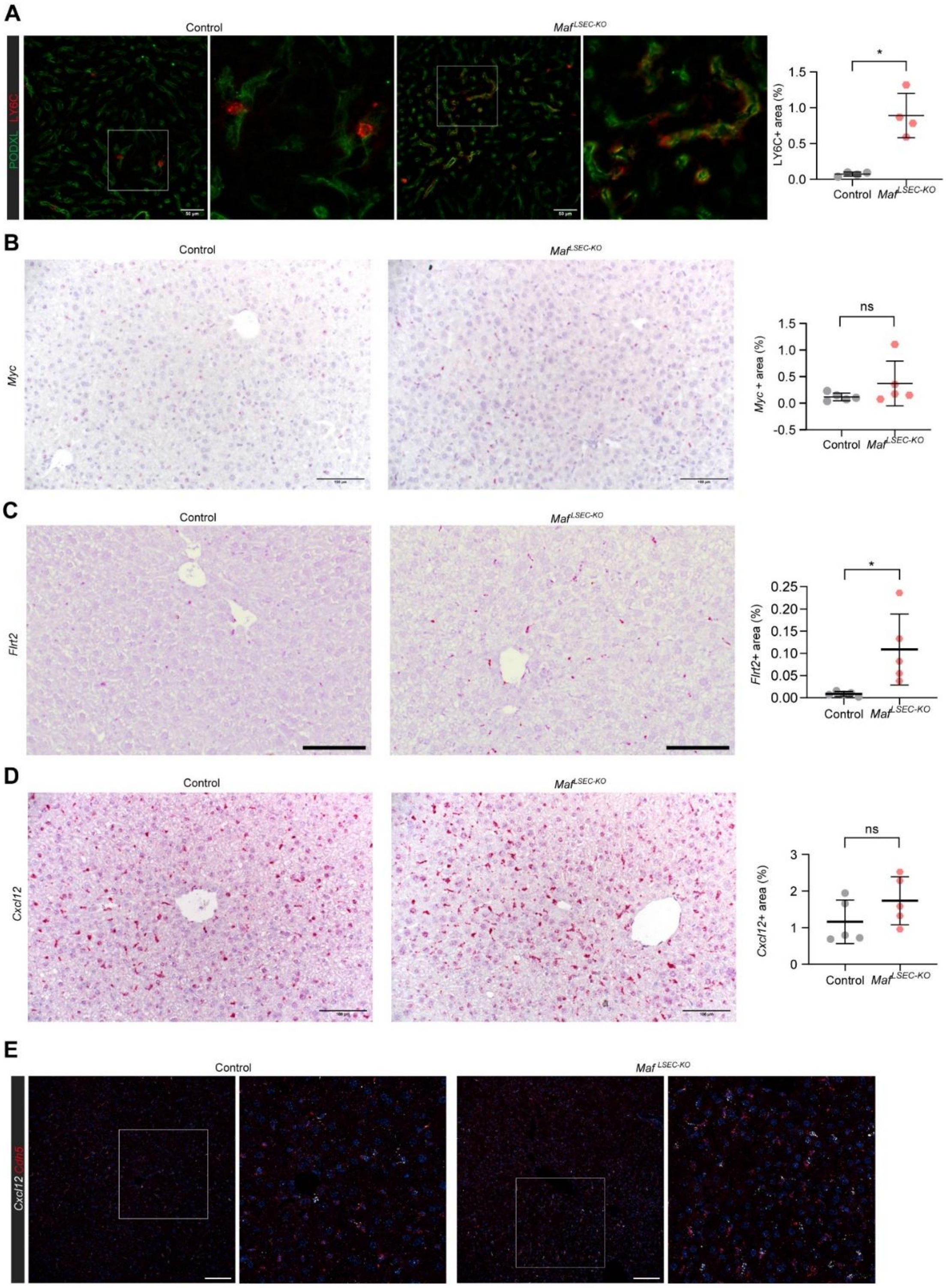

Fig. S6. Immunofluorescence staining and *in situ* hybridization of dysregulated genes identified by bulk RNA-seq.

(A) Immunofluorescence staining with zoom-in for PODXL and LY6C and quantification of LY6C area (n = 5). (B) *In situ* hybridization and quantification of (B) *Myc,* (C) *Flrt2,* and (D) *Cxcl12* (n = 5). (E) FISH of *Cxcl12* and *Cdh5* with zoom-in (n = 5). (B) Mann-Whitney *U* test; (A, C-E) Welch's *t* test. n.s. $p \geq .05$; * $p < .05$.

Fig. S7

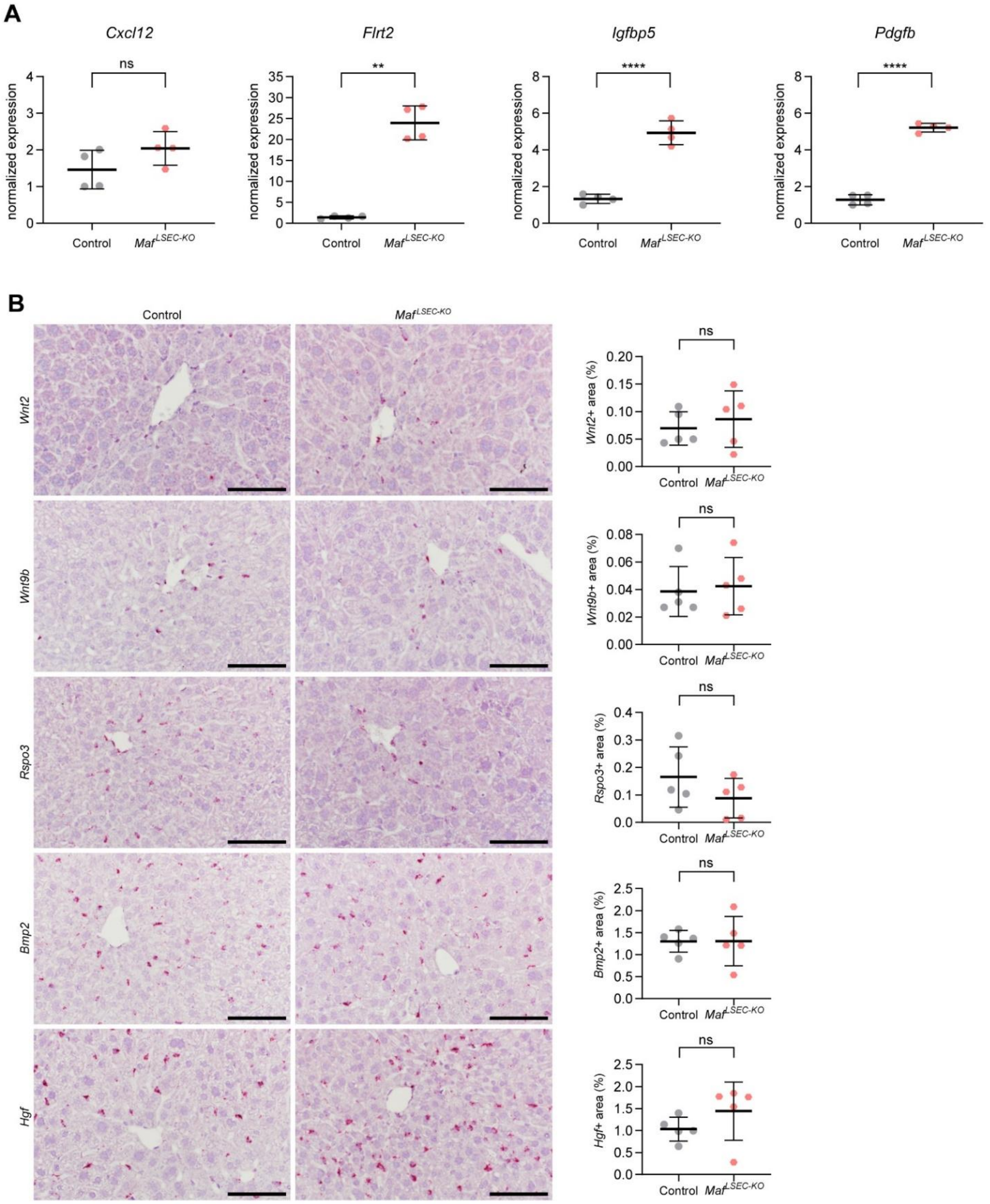

Fig. S7. *In situ* hybridization of hepatic angiocrine factors.

(A) qPCR of *Cxcl12*, *Flrt2*, *Igfbp5,* and *Pdgfb* using RNA from isolated LSEC (n = 4). (B) *In situ* hybridization and quantification for *Wnt2, Wnt9b, Rspo3, Bmp2* and *Hgf* (n = 5). Mean ± SD. (B [*Wnt2, Rspo3, Bmp2*]) Welch's *t* test. (B [Wnt9b, Hgf] Mann-Whitney *U* test. n.s. $p \geq .05$.; * $p < .05$.

Fig. S8

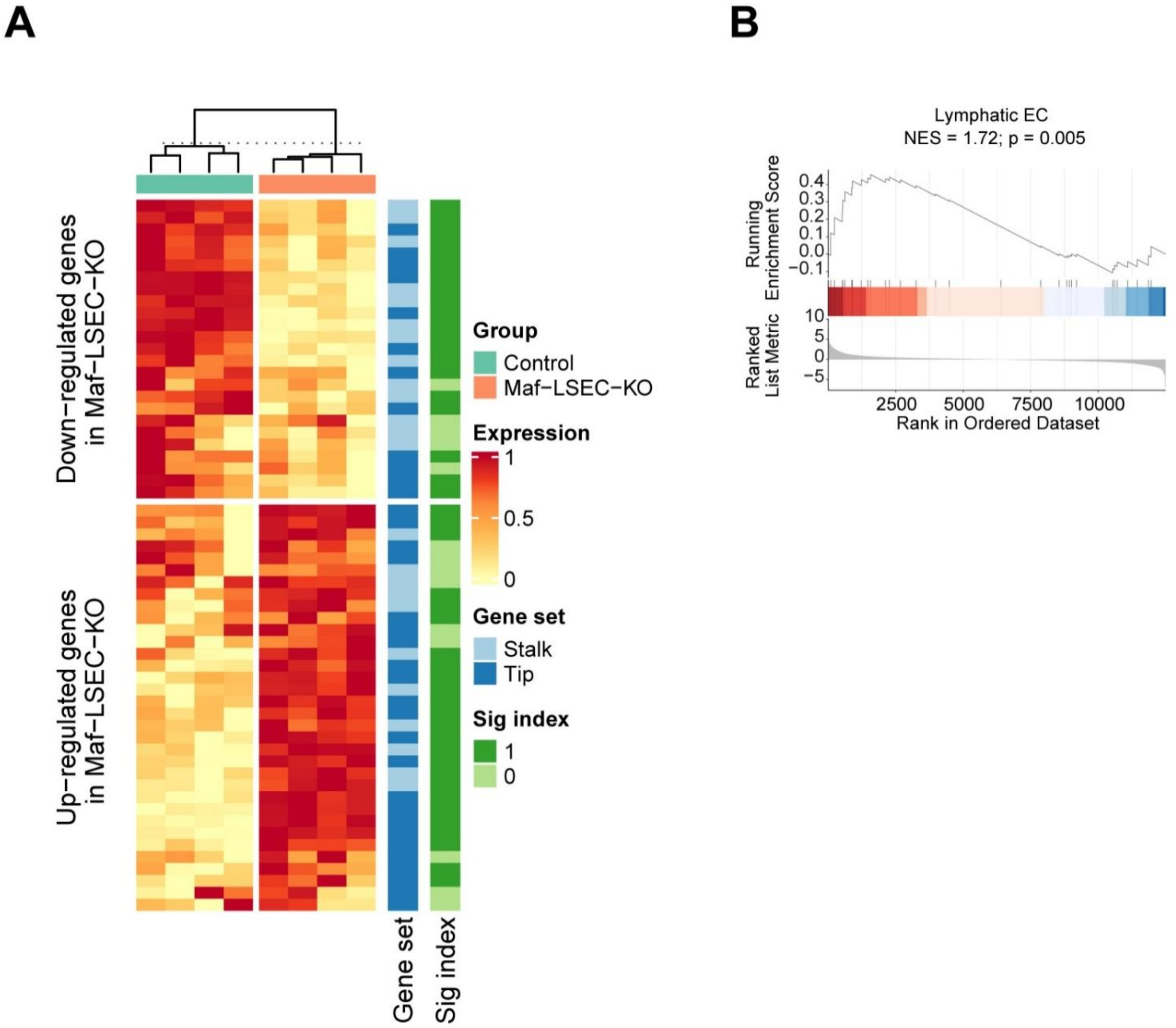

Fig. S8. Heatmap of angiogenesis related genes and enrichment analysis for lymphatic EC.

(A) Heatmap of tip and stalk cell genes in RNA-seq data from LSEC. (B) Enrichment plot for lymphatic EC associated genes. NES, normalized enrichment score.

Fig. S9

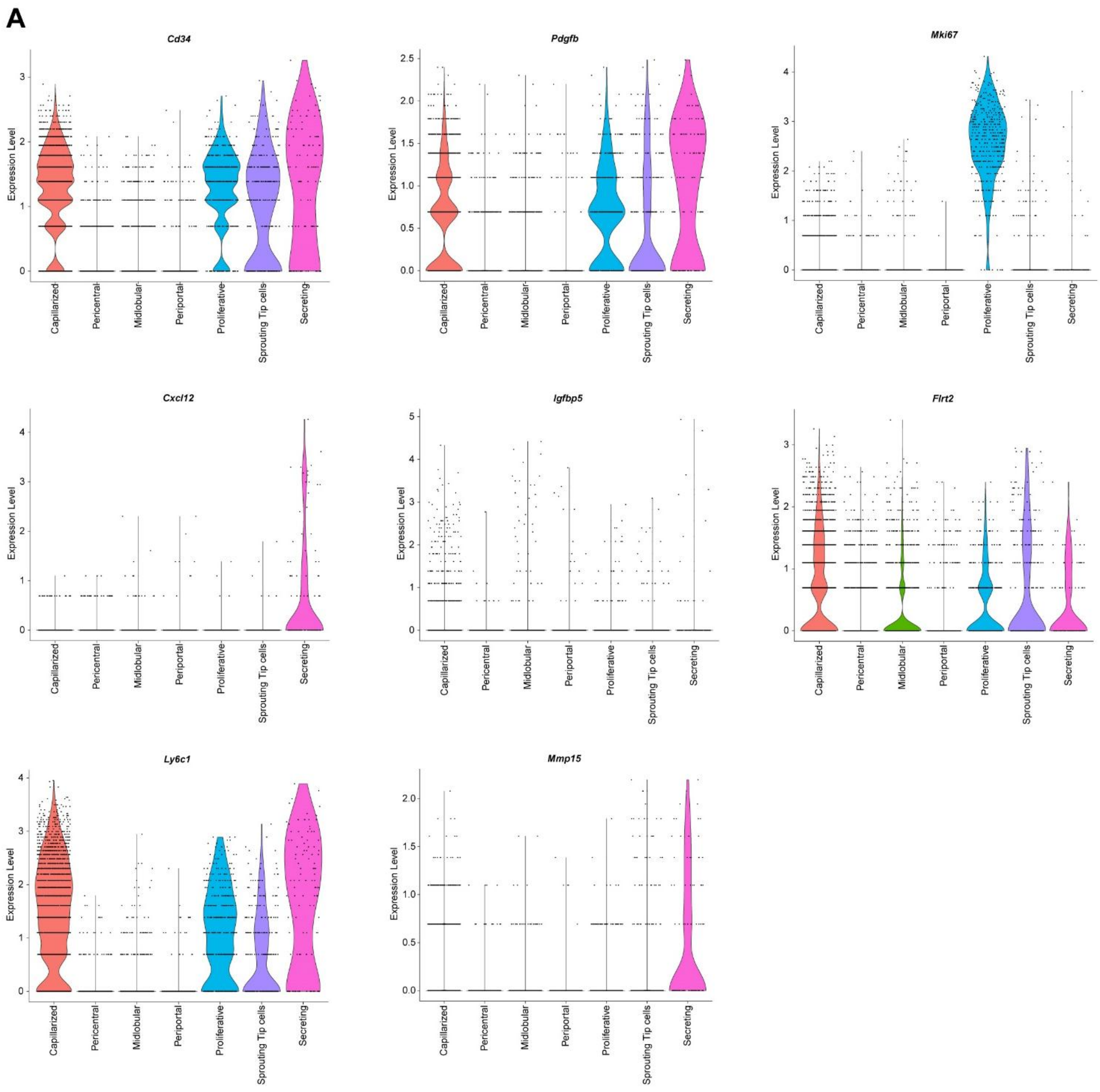

Fig. S9. Gene expressions for genes of interest in scRNA-seq of LSEC.

(A) Violin plots for the genes of interest (*Cd34*, *Pdgfb*, *Mki67*, *Cxcl12, Igfbp5*, *Flrt2, Ly6c1,* and *Mmp15*).

Fig. S10

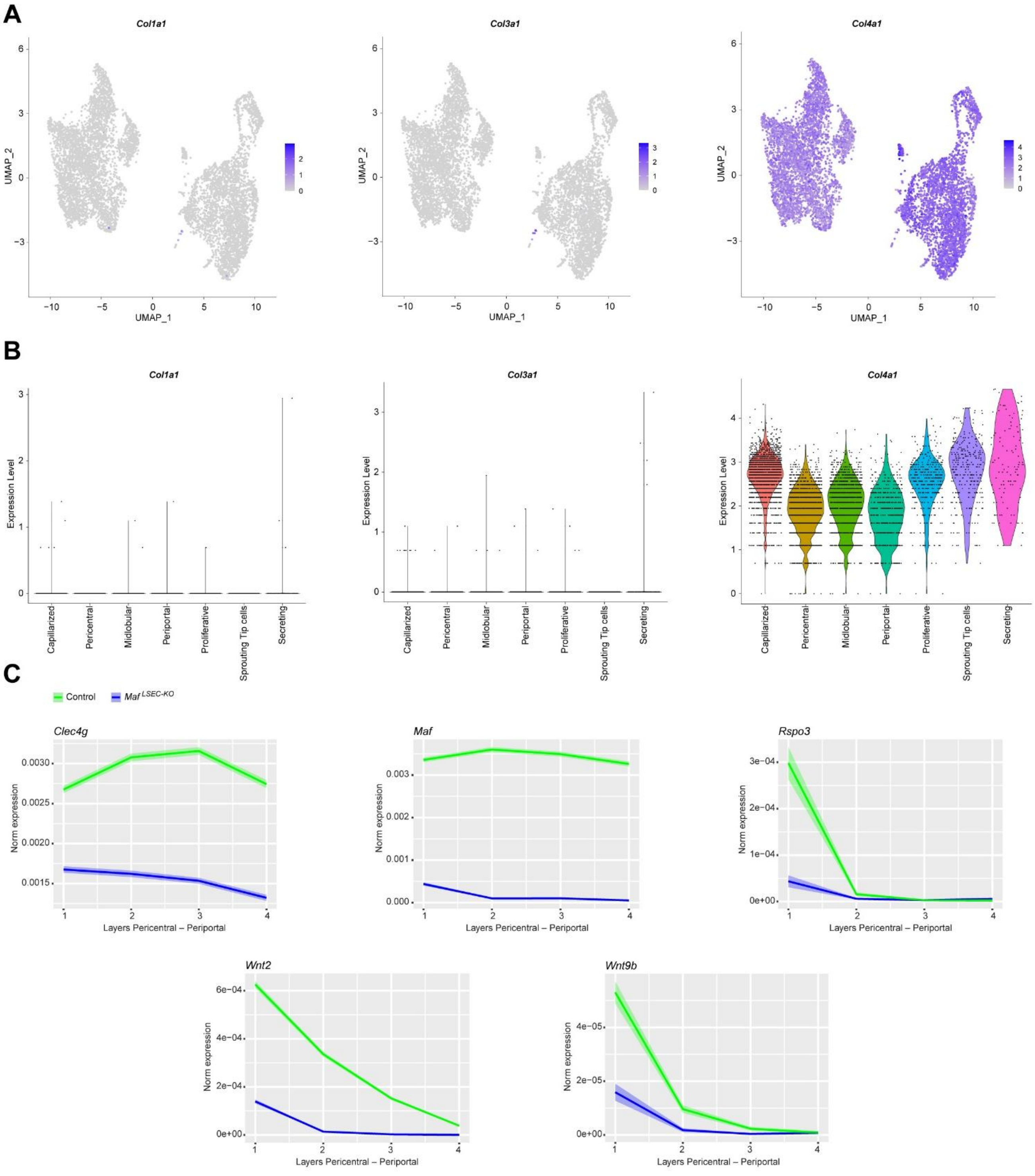

Fig. S10. Gene expressions for genes of interest and zonated expression of Wnt factors in scRNA-seq of LSEC. Annotation for the genes of interest (*Col1a1*, *Col3a1* and *Col4a1*) in (A) UMAP plot and (B) violin plot. (C) Gene expressions for *Clec4g*, *Maf*, and Wnt factors (*Rspo3*, *Wnt2*, and *Wnt9b*) in LSEC zones ranging from pericentral (1) to periportal (4).

Table S1

Results of bulk RNA-seq analysis of hepatic endothelial cells from *Maf*$^{LSEC-KO}$ vs. control mice

See separate Excel file: Table_S1_bulk-RNA-seq_LSEC_Maf-LSEC-KO_vs_Controls.xlsx

Table S2

Results of ATAC-seq analysis of hepatic endothelial cells from *Maf*$^{LSEC-KO}$ vs. control mice

See separate Excel file: Table_S2_ATAC-seq_LSEC_Maf-LSEC-KO_vs_Controls.xlsx

Table S3

TOBIAS footprint analysis

See separate Excel file:

Table_S3_ATAC-seq_LSEC_TOBIAS_footprints_Maf-LSEC-KO_vs_Controls.xlsx

Table S4

Directly c-Maf regulated genes identified by downregulated c-Maf footprints and significant dysregulation in bulk RNA-seq data of hepatic endothelial cells

See separate Excel file:

Table_S4_Genelist_c-Maf-footprint_down_and_adjp_0.05_in_bulk-RNA-seq.xlsx

Supplementary References

[1] Schneider CA, Rasband WS, Eliceiri KW. NIH Image to ImageJ: 25 years of image analysis. Nat Methods 2012;9:671–5. https://doi.org/10.1038/nmeth.2089.

[2] Schindelin J, Arganda-Carreras I, Frise E, et al. Fiji: an open-source platform for biological-image analysis. Nature Methods 2012;9:676–82. https://doi.org/10.1038/nmeth.2019.

[3] Hruz T, Wyss M, Docquier M, et al. RefGenes: identification of reliable and condition specific reference genes for RT-qPCR data normalization. BMC Genomics 2011;12:156. https://doi.org/10.1186/1471-2164-12-156.

[4] Winkler M, Staniczek T, Kürschner SW, et al. Endothelial GATA4 controls liver fibrosis and regeneration by preventing a pathogenic switch in angiocrine signaling. Journal of Hepatology 2021;74:380–93. https://doi.org/10.1016/j.jhep.2020.08.033.

[5] H Backman TW, Girke T. systemPipeR: NGS workflow and report generation environment. BMC Bioinformatics 2016;17:388. https://doi.org/10.1186/s12859-016-1241-0.

[6] Bray NL, Pimentel H, Melsted P, et al. Near-optimal probabilistic RNA-seq quantification. Nat Biotechnol 2016;34:525–7. https://doi.org/10.1038/nbt.3519.

[7] Ritchie ME, Phipson B, Wu D, et al. limma powers differential expression analyses for RNA-sequencing and microarray studies. Nucleic Acids Research 2015;43:e47. https://doi.org/10.1093/nar/gkv007.

[8] Gu Z, Eils R, Schlesner M. Complex heatmaps reveal patterns and correlations in multidimensional genomic data. Bioinformatics 2016;32:2847–9. https://doi.org/10.1093/bioinformatics/btw313.

[9] Xu S, Hu E, Cai Y, et al. Using clusterProfiler to characterize multiomics data. Nat Protoc 2024:1–29. https://doi.org/10.1038/s41596-024-01020-z.

[10] Su T, Yang Y, Lai S, et al. Single-Cell Transcriptomics Reveals Zone-Specific Alterations of Liver Sinusoidal Endothelial Cells in Cirrhosis. Cellular and Molecular Gastroenterology and Hepatology 2021;11:1139–61. https://doi.org/10.1016/j.jcmgh.2020.12.007.

[11] Corces MR, Trevino AE, Hamilton EG, et al. An improved ATAC-seq protocol reduces background and enables interrogation of frozen tissues. Nat Methods 2017;14:959–62. https://doi.org/10.1038/nmeth.4396.

[12] Heinz S, Benner C, Spann N, et al. Simple combinations of lineage-determining transcription factors prime cis-regulatory elements required for macrophage and B cell identities. Mol Cell 2010;38:576–89. https://doi.org/10.1016/j.molcel.2010.05.004.

[13] Wang Q, Li M, Wu T, et al. Exploring Epigenomic Datasets by ChIPseeker. Current Protocols 2022;2:e585. https://doi.org/10.1002/cpz1.585.

[14] Ben-Moshe S, Veg T, Manco R, et al. The spatiotemporal program of zonal liver regeneration following acute injury. Cell Stem Cell 2022;29:973-989.e10. https://doi.org/10.1016/j.stem.2022.04.008.

[15] Butler A, Hoffman P, Smibert P, et al. Integrating single-cell transcriptomic data across different conditions, technologies, and species. Nat Biotechnol 2018;36:411–20. https://doi.org/10.1038/nbt.4096.

[16] McGinnis CS, Murrow LM, Gartner ZJ. DoubletFinder: Doublet Detection in Single-Cell RNA Sequencing Data Using Artificial Nearest Neighbors. Cell Syst 2019;8:329-337.e4. https://doi.org/10.1016/j.cels.2019.03.003.

[17] Kalucka J, de Rooij LPMH, Goveia J, et al. Single-Cell Transcriptome Atlas of Murine Endothelial Cells. Cell 2020;180:764-779.e20. https://doi.org/10.1016/j.cell.2020.01.015.

[18] Halpern KB, Shenhav R, Massalha H, et al. Paired-cell sequencing enables spatial gene expression mapping of liver endothelial cells. Nature Biotechnology 2018;36:962. https://doi.org/10.1038/nbt.4231 https://www.nature.com/articles/nbt.4231#supplementary-information.

[19] Ramachandran P, Dobie R, Wilson-Kanamori JR, et al. Resolving the fibrotic niche of human liver cirrhosis at single-cell level. Nature 2019;575:512–8. https://doi.org/10.1038/s41586-019-1631-3.

[20] Hafemeister C, Satija R. Normalization and variance stabilization of single-cell RNA-seq data using regularized negative binomial regression. Genome Biology 2019;20:296. https://doi.org/10.1186/s13059-019-1874-1.

[21] Germain P-L, Lun A, Garcia Meixide C, et al. Doublet identification in single-cell sequencing data using scDblFinder. F1000Res 2021;10:979. https://doi.org/10.12688/f1000research.73600.2.

[22] Guilliams M, Bonnardel J, Haest B, et al. Spatial proteogenomics reveals distinct and evolutionarily conserved hepatic macrophage niches. Cell 2022;185:379-396.e38. https://doi.org/10.1016/j.cell.2021.12.018.

[23] Nagy D, Maude H, Birdsey GM, et al. RISING STARS: Liver sinusoidal endothelial transcription factors in metabolic homeostasis and disease. J Mol Endocrinol 2023;71:e230026. https://doi.org/10.1530/JME-23-0026.
