# Supplementary material for: Endothelial c-Maf prevents MASLD-like liver fibrosis by regulating chromatin accessibility to suppress pathogenic microvascular cell subsets
Source: JHEP Rep. 2025 Jun 6;7(9):101475. doi: 10.1016/j.jhepr.2025.101475 (PMC12341620; doi:10.1016/j.jhepr.2025.101475)
Supplement: Multimedia component 2 [file mmc2.docx]

**JHEP Reports**

**CTAT methods**

Tables for a “Complete, Transparent, Accurate and Timely account” (CTAT) are now mandatory for all revised submissions. The aim is to enhance the reproducibility of methods.

- Only include the parts relevant to your study
- Refer to the CTAT in the main text as ‘Supplementary CTAT Table’
- Do not add subheadings
- Add as many rows as needed to include all information
- Only include one item per row

**If the CTAT form is not relevant to your study, please outline the reasons why:**

|  |
| --- |

- 1. **Antibodies**

| **Name** | **Citation** | **Supplier** | **Cat no.** | **Clone no.** |
| --- | --- | --- | --- | --- |
| Rabbit anti-mouse/human alpha smooth muscle Actin | AB_2223021 | Abcam | ab5694 | N/A |
| Rabbit anti-mouse Arginase-1 | RRID: AB_2800207 | Cell Signaling Technology | #93668 | N/A |
| Rat anti-mouse/human CD11b | PRID: AB_312785 | BioLegend | 101202 | M1/70 |
| Rat anti-mouse CD31 | PRID: AB_2631039 | Dianova | DIA-310 | SZ31 |
| Goat anti-mouse CD32/CD16 | PRID: AB_354811 | R&D Systems | AF1460 | N/A |
| Rabbit anti-mouse Collagen type I | RRID: AB_978381 | Acris Antibodies GmbH | R1038 | N/A |
| Rabbit anti-human Collagen type IV | RRID: AB_789360 | Novus Biological | NB120-6586 | N/A |
| Rabbit anti-mouse CYP2E1 | PRID: AB_1078613 | Sigma-Aldrich | HPA009128 | N/A |
| Rat anit-mouse Endomucin | RRID: AB_10859306 | Abcam | Ab106100 | N/A |
| Rabbit anti-mouse F4/80 | PRID: AB_2798990 | Cell Signaling Technology | 30325 | D4C8V |
| Guinea pig anti-Glutamine Synthetase | PRID: AB_2620128 | Synaptic Systems | 367 005 | N/A |
| Rabbit anti-human HAL | PRID:  AB_1850484 | Sigma-Aldrich | AV45694 | N/A |
| Rabbit anti-mouse/human Ki67 | RRID: AB_302459 | Abcam | ab16667 | SP6 |
| Rat anti-mouse Ly6c | PRID: AB_302004 | Abcam | ab15627 | ER-MP20 |
| Goat anti-mouse Lyve-1 | RRID: AB_2297188 | R&D Systems | AF2125 | N/A |
| Goat anti mouse/human PDGF R beta | RRID:  AB_2162639 | Neuromics | GT15065-100 | N/A |
| Goat anti-mouse Podocalyxin | RRID: AB_354858 | R&D Systems | AF1556 | N/A |
| Goat anti-mouse RHBG | PRID: AB_10990341 | Thermo Fisher Scientific | PA5-19369 | N/A |
| Rabbit anti-mouse Stabilin2 c-Term Pep 15 |  |  | M881-P15 |  |
| HRP conjugated goat anti-rabbit IgG | RRID:  AB_2630375 | Agilent | K4003 | N/A |
| Cy™3 AffiniPure Donkey Anti-Rabbit IgG |  | Dianova | 711-165-152 | N/A |
| Alexa Fluor 647-AffiniPure Donkey Anti-Goat IgG | RRID:  AB_2340437 | Dianova | 705-605-147 | N/A |
| Cy™3 AffiniPure Donkey Anti-Rat IgG |  | Dianova | 712-165-153 | N/A |
| Alexa Fluor 488-AffiniPure Donkey Anti-Rat IgG |  | Dianova | 712-545-153 | N/A |
| Alexa Fluor 488-AffiniPure Donkey Anti-Guinea Pig IgG |  | Dianova | 706-546-148 | N/A |
| Brilliant Violet 510™ anti-mouse/human CD11b Antibody | RRID: AB_2561390 | Biolegend | 101245 | M1/70 |
| PerCP/Cyanine5.5 anti-mouse CD31 Antibody | RRID: AB_2566761 | Biolegend | 102522 | MEC13.3 |
| Mouse LYVE-1 PE-conjugated Antibody | RRID: AB_10889020 | R&D | FAB2125P | 223322 |
| BD Horizon™ Fixable Viability Stain 780 | RRID: AB_2869673 | BD | 565388 | N/A |

- 1. **Cell lines**

| **Name** | **Citation** | **Supplier** | **Cat no.** | **Passage no.** | **Authentication test method** |
| --- | --- | --- | --- | --- | --- |
| LX-2 | NA | Sigma-Aldrich | SCC064 | 12 | STR Analysis |

- 1. **Organisms**

| **Name** | **Citation** | **Supplier** | **Strain** | **Sex** | **Age** | **Overall n number** |
| --- | --- | --- | --- | --- | --- | --- |
| Mouse:  Maf^LSEC-KO^  C57BL/6 -Tg(Clec4g-icre)1.1Sgoe Maftm2.1Cbm | NA | Homemade | B6 | Female/male | 3 – 5 months | 300 |
| Mouse:  Clec4g-iCre C57BL/6N-Tg(Clec4g-icre)1.1Sgoe | Cancer Res. 2019. 1;79(3):598-610 | Homemade | B6 | Male | 2 – 6 months | Crossed with floxed mice |
| Mouse:  B6.129P2-Maftm2.1Cbm | Science. 2012 Mar 16;335(6074):1373-6 | Max-Delbrück-Centrum (MDC) | B6.129P2 | Female | 2 – 6 months | Crossed with Cre mice |

- 1. **Sequence based reagents**

| **Name** | **Sequence** | **Supplier** |
| --- | --- | --- |
| **Primer for qRT-PCR** |  |  |
| Human *CACTIN*_Forward | TGCTGGAGGATATCCAGGTCT |  |
| Human *CACTIN*_Reverse | GCTTGGAGATCTCGTCCTCG |  |
| Human *COL1A1*_Forward | GACCTGGTAGCCGTGGTTTC | metabion international AG |
| Human *COL1A1*_Reverse | CTTCCAGTCAGACCCTTGGC | metabion international AG |
| Human *HLCS*_Forward | ACCAACATGGAGGCCTTCTC | metabion international AG |
| Human *HLCS*_Reverse | CTGAAACATCAGCCCATCCAG | metabion international AG |
| Mouse *Acta2*_Forward | CAGACATCAGGGAGTAATGGTTG | metabion international AG |
| Mouse *Acta2*_Reverse | GGCCACACGAAGCTCGTTAT | metabion international AG |
| Mouse *Col1a1*_Forward | CAGGCTGGTGTGATGGGATT | metabion international AG |
| Mouse *Col1a1*_Reverse | AAACCTCTCTCGCCTCTTGC | metabion international AG |
| Mouse *Col3a1* Forward | GAGGAATGGGTGGCTATCCG | metabion international AG |
| Mouse *Col3a1*_Reverse | GCGTCCATCAAAGCCTCTGT | metabion international AG |
| Mouse *Cxcl12*_Forward | GGAGAAAGCTTTAAACAAGAGGCT | metabion international AG |
| Mouse *Cxcl12*_Reverse | GCTATGGGCCCTTCCCTAAC | metabion international AG |
| Mouse *Des*_Forward | GAGGAGAGCAGGATCAACCTT | metabion international AG |
| Mouse *Des*_Reverse | CTCTCCATCCCGGGTCTCAA | metabion international AG |
| Mouse *Flrt2*_Forward | CAGACTGGCAGTTCTCAACGA | metabion international AG |
| Mouse *Flrt2*_Reverse | TGCAGAGTCTGTAATCTGGC | metabion international AG |
| Mouse *Gak*_Forward | CTGCCCACCAGGCATTTG | metabion international AG |
| Mouse *Gak*_Reverse | CCATGTCACATACATATTCAATGTACCT | metabion international AG |
| Mouse *Igfbp5*_Forward | CCTGCACCTGAGATGAGACAG | metabion international AG |
| Mouse *Igfbp5*_Reverse | ACCAGCAGATGCCACGTTTG | metabion international AG |
| Mouse *Mrpl46*_Forward | GGGAGCAGGCATTCCTACAG | metabion international AG |
| Mouse *Mrpl46*_Reverse | GGTCCGGTCATTTTTTTTGTCA | metabion international AG |
| Mouse *Pdgfb*_Forward | CTACCTGCGTCTGGTCAGC | metabion international AG |
| Mouse *Pdgfb*_Reverse | GCTCAGCCCCATCTTCATCTAC | metabion international AG |
| Mouse *Pdgfrb*_Forward | ATGGGTGGAGATTCGCAGGA | metabion international AG |
| Mouse *Pdgfrb*_Reverse | TCATAGCGTGGCTTCTTCTGCC | metabion international AG |
| Mouse *Srp72*_Forward | CACCCAGCAGACAGACAAACTG | metabion international AG |
| Mouse *Srp72*_Reverse | GCACTCATCGTAGCGTTCCA | metabion international AG |
| **Primers for Genotyping** |  |  |
| Mouse *Clec4g-iCre*_Forward | AAGCTGAACAACAGGAAATGGTTC | metabion international AG |
| Mouse *Clec4g-iCre*_Reverse | GGAGATGTCCTTCACTCTGATTCT | metabion international AG |
| Mouse *Maf*_Forward | ATGATCAGGCTCAGGCTTAA | metabion international AG |
| Mouse *Maf*_Reverse | CGCACCCTGACAACGTG | metabion international AG |

- 1. **Biological samples**

| **Description** | **Source** | **Identifier** |
| --- | --- | --- |
|  |  |  |

- 1. **Deposited data**

| **Name of repository** | **Identifier** | **Link** |
| --- | --- | --- |
| Gene Expression Omnibus | [GSE277535](https://www.ncbi.nlm.nih.gov/geo/query/acc.cgi?acc=GSE277535) | https://www.ncbi.nlm.nih.gov/geo/query/acc.cgi?acc=[GSE277535](https://www.ncbi.nlm.nih.gov/geo/query/acc.cgi?acc=GSE277535)  (bulk RNA-seq of hepatic EC), |
| Gene Expression Omnibus | GSE277933 | <https://www.ncbi.nlm.nih.gov/geo/query/acc.cgi?acc=GSE277933>  (ATAC-seq of hepatic EC) |
| Gene Expression Omnibus | GSE281716 | <https://www.ncbi.nlm.nih.gov/geo/query/acc.cgi?acc=GSE281716>  (scRNA-seq of hepatic EC) |

- 1. **Software**

| **Software name** | **Manufacturer** | **Version** |
| --- | --- | --- |
| Fiji ImageJ | Open Source/National  Institutes of Health | 1.54f |
| NIS-Elements AR | Nikon Instruments | 5.30.6 |
| GraphPad Prism 10 | GraphPad Software, Inc. | 10.2.3. |
| R | Open Source/R Core Team | 3.6.3 |
| qPCRsoft | Analytik Jena | 4.0.8.0 |
| FlowJo | FlowJo, LLC | 10.7.2 |
| BioRender | BioRender |  |
| Bioconductor |  | 3.9 |
| Adobe Illustrator | Adobe | 28.7.1 |

- 1. **Other (*e.g*. drugs, proteins, vectors etc.)**

| Tissue Collagen Assay | QZBtiscol1 | QuickZyme |
| --- | --- | --- |
| CDAA diet | ssniff | E15666-94 |
| Triglyceride Assay Kit - Quantification | Abcam | ab65336 |
| **Probe** | **Supplier** | **Order No.** |
| Mm-Bmp2-E3 | Advanced Cell Diagnostics | 427341 |
| Mm-Cd34-C2 | Advanced Cell Diagnostics | 319161-C2 |
| Mm-Cdh5-C2 | Advanced Cell Diagnostics | 312531-C2 |
| Mm-Col1a1 | Advanced Cell Diagnostics | 319371 |
| Mm-Col3a1 | Advanced Cell Diagnostics | 455771 |
| Mm-Col4a1 | Advanced Cell Diagnostics | 412871 |
| Mm-Cxcl12 | Advanced Cell Diagnostics | 422711 |
| Mm-Esm1-E3 | Advanced Cell Diagnostics | 411761 |
| Mm-Flrt2 | Advanced Cell Diagnostics | 490291 |
| Mm-Hgf | Advanced Cell Diagnostics | 315631 |
| Mm-Igfbp5 | Advanced Cell Diagnostics | 425731 |
| Mm-Maf | Advanced Cell Diagnostics | 412951 |
| Mm-Myc | Advanced Cell Diagnostics | 413451 |
| Mm-Pdgfb | Advanced Cell Diagnostics | 424651 |
| Mm-Pdgfrb | Advanced Cell Diagnostics | 411381 |
| Mm-Pdgfrb-C2 | Advanced Cell Diagnostics | 411381-C2 |
| Mm-Rspo3 | Advanced Cell Diagnostics | 402011 |
| Mm-Sparcl1 | Advanced Cell Diagnostics | 424641 |
| Mm-Wnt2 | Advanced Cell Diagnostics | 313601 |
| Mm-Wnt9b | Advanced Cell Diagnostics | 405091 |
| Positive Control Probe Mm-Ppib | Advanced Cell Diagnostics | 313911 |
| Negative Control Probe DapB | Advanced Cell Diagnostics | 310043 |
| **Proteins** | **Supplier** | **Order No.** |
| Recombinant Human/Rhesus Macaque/Feline CXCL12/SDF-1 alpha | Bio-Techne | 350-NS-050/CF |
| Recombinant Human FLRT2 Protein, CF | Bio-Techne | 2877-FL-050 |
| Recombinant Human IGFBP-5 Protein, CF | Bio-Techne | 875-B5-025 |
| Recombinant Human PDGF-BB Protein, CF | Bio-Techne | 220-BB-010 |
| RNAscope 2.5 HD Duplex Kit | Advanced Cell Diagnostics | 322430 |
| innuPREP RNA Mini Kit 2.0 | Analytik Jena | 845-KS-2040250 |
| Oligo(dT)18 primer | Thermo Fisher Scientific | SO131 |
| Collagenase A | Sigma-Aldrich | C2674 |
| Gey’s balanced salt solution | Sigma-Aldrich | G9779 |
| Nycodenz | Axis-Shield | 1002424 |
| anti-CD146 MicroBeads | Miltenyi Biotech | ME-9F1, 130-092-007 |
| NP-40 | Merck | 74385 |
| Isofluran CP | WDT | 21311 |
| 4 % formaldehyde solution | Carl Roth | P087 |
| HIER citrate buffer pH 6.0 | Zytomed Sytems | ZUC028-500 |
| 5 % normal donkey serum | Dianova | 017-000-121 |
| Dako antibody diluent | Agilent Technologies | S202230-2 |
| Phosphate-buffered saline | VWR International | A0964.9050, |
| Dako fluorescence mounting medium | Agilent Technologies | S302380-2 |
| Meyer’s hemalum solution | Merck | 1.09249.2500 |
| Dako aqueous mounting medium | Agilent Technologies | S3025 |

- 1. **Please provide the details of the corresponding methods author for the manuscript:**

| **Christian David Schmid** |
| --- |

**2.0 Please confirm for randomised controlled trials all versions of the clinical protocol are included in the submission. These will be published online as supplementary information.**

| **NA** |
| --- |
