# Supplementary material for: Endothelial c-Maf prevents MASLD-like liver fibrosis by regulating chromatin accessibility to suppress pathogenic microvascular cell subsets
Source: JHEP Rep. 2025 Jun 6;7(9):101475. doi: 10.1016/j.jhepr.2025.101475 (PMC12341620; doi:10.1016/j.jhepr.2025.101475)
Supplement: Multimedia component 8 [file mmc8.pdf]

JHEP|Reports

Endothelial c-Maf prevents MASLD-like liver fibrosis by regulating chromatin accessibility to suppress pathogenic microvascular cell subsets

Authors

Manuel Winkler, Theresa Staniczek, Maximilian Suhayda, …, Philipp-Sebastian Reiners-Koch, Sergij Goerdt, Christian David Schmid

Correspondence

christian.schmid@medma.uni-heidelberg.de (C.D. Schmid).

Graphical abstract

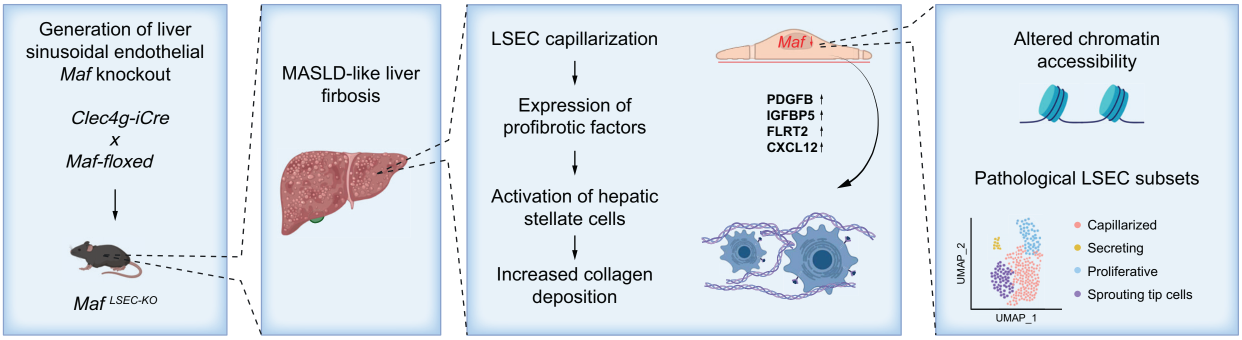

Highlights:

- Endothelial c-Maf prevents MASLD-like liver fibrosis.
- c-Maf is a master regulator of LSEC identity.
- c-Maf prevents development of pathogenic profibrotic endothelial cell subsets.
- c-Maf controls chromatin accessibility of LSEC.
- *MAF* is downregulated in human cirrhosis.

Impact and implications:

This work builds on the known importance of liver sinusoidal endothelial cells in liver function and disease. Here, transcription factor c-Maf is identified as a master regulator in maintaining normal differentiation and zonation of liver sinusoidal endothelial cells, thereby protecting against the development of liver fibrosis/cirrhosis. The findings are significant for researchers and clinicians focusing on liver disease, as they suggest potential new targets for therapeutic intervention. These findings could instruct the development of novel preventive treatment options and antifibrotic therapy regimens as well as liver repair strategies, benefiting patients, clinicians and policy makers in the management of liver disease.

https://doi.org/10.1016/j.jhepr.2025.101475
© 2025 The Author(s). Published by Elsevier B.V. on behalf of European Association for the Study of the Liver (EASL). This is an open access article under the CC BY license (http://creativecommons.org/licenses/by/4.0/). JHEP Reports, 2025, 7, 1–15

Endothelial c-Maf prevents MASLD-like liver fibrosis by regulating chromatin accessibility to suppress pathogenic microvascular cell subsets

Manuel Winkler[1,†], Theresa Staniczek[1,†], Maximilian Suhayda[1,†], Sina Wietje Kürschner-Zacharias[1], Johannes Hoffmann[1], Julio Cordero[2], Linda Kraske[2], Hannah Maude[3], Dorka Nagy[3,4], Rita Manco[5], Carsten Sticht[6], Michelle Neßling[7], Karsten Richter[7], Gergana Dobreva[2], Anna Maria Randi[4], Inês Cebola[3], Kai Schledzewski[1], Philipp-Sebastian Reiners-Koch[1,8,9], Sergij Goerdt[1,8], Christian David Schmid[1,*]

JHEP Reports **2025**. vol. 7 | 1–15

Check for updates

Background & Aims: Liver sinusoidal endothelial cells (LSECs) are highly specialized components of the hepatic vascular niche, regulating liver function and disease pathogenesis through angiocrine signaling. Recently, we identified GATA4 as a key transcription factor controlling LSEC development and protecting against liver fibrosis. As the transcription factor c-Maf was strongly downregulated in *Gata4*-deficient LSECs, we hypothesized that c-Maf might be an important downstream effector of GATA4 in LSEC differentiation and liver fibrogenesis.

Methods: *Clec4g-iCre/Maf$^{fl/fl}$* (*Maf$^{LSEC\text{-}KO}$*) mice with LSEC-specific *Maf* deficiency were generated and liver tissue was analyzed histologically. LSECs were isolated for bulk RNA-seq, ATAC-seq, and single-cell (sc) RNA-seq analysis. *Maf$^{LSEC\text{-}KO}$* livers were analyzed after MASH diet feeding. The expression of *MAF* and its targets was analyzed in published human scRNA-seq data.

Results: Endothelial *Maf* deficiency resulted in perisinusoidal liver fibrosis (Sirius red 0.46% *vs*. 2.92%; *p* <0.05) without affecting metabolic liver zonation, accompanied by a switch from sinusoidal to continuous endothelial cell identity, which was aggravated upon MASH diet feeding (*p* <0.01). Furthermore, endothelial *Maf* deficiency caused LSEC proliferation (*p* <0.05) and expression of profibrotic angiocrine factors including *Pdgfb*, *Igfbp5*, *Flrt2, and Cxcl12*, among which FLRT2 (*p* <0.01) and CXCL12 (*p* <0.001) activated hepatic stellate cells *in vitro*. scRNA-seq revealed replacement of zonated LSEC subpopulations with capillarized, proliferative, sprouting and secretory endothelial cell subsets that promote liver fibrogenesis and angiogenesis. This fundamental dysregulation of LSEC gene expression and differentiation was caused by changes in chromatin accessibility and transcription factor activity following loss of *Maf*. Notably, endothelial *MAF* expression was also significantly reduced in human cirrhotic livers (*p* <0.0001).

Conclusions: Hepatic endothelial c-Maf protects against metabolic dysfunction-associated steatohepatitis-like liver fibrosis and regulates endothelial differentiation and zonation by controlling chromatin opening.

© 2025 The Author(s). Published by Elsevier B.V. on behalf of European Association for the Study of the Liver (EASL). This is an open access article under the CC BY license (http://creativecommons.org/licenses/by/4.0/).

Introduction

Liver sinusoidal endothelial cells (LSECs) represent a distinct organotypic endothelial cell (EC) subtype showing remarkable morphological and functional specialization. LSECs are discontinuous, exhibiting fenestrations and lacking a basement membrane, and together with resident hepatic macrophages, *i.e*. hepatic Kupffer cells, form a sinusoidal interface that allows direct interactions with stellate cells and hepatocytes, as well as easy exchange of blood cellular elements, nutrients and metabolites. LSECs play a central role in regulating blood flow and portal pressure, as well as in hepatic immune surveillance.[1] In addition, LSECs control liver functions via angiocrine signaling. We and others have shown, for example, that iron metabolism is regulated by LSEC angiokines BMP2[2] and BMP6,[3] whereas metabolic liver zonation is controlled by angiocrine Wnt signaling.[4]

The establishment and maintenance of LSEC identity are regulated by signaling pathways and endothelial transcription factors. For example, we have shown that ALK1 signaling regulates hepatic vessel formation, angiodiversity, and angiocrine functions, thereby preventing hereditary hemorrhagic telangiectasia of the liver.[5] Additionally, both our group and others have demonstrated the critical role of balanced Notch signaling in maintaining LSEC integrity.[6,7]

Few transcription factors have been described to control LSEC differentiation and liver functions. Loss of the ubiquitous endothelial transcription factor ERG caused endothelial-mesenchymal transition in LSECs and led to the development

* Corresponding author. Address: Department of Dermatology, Venereology and Allergology, University Medical Center and Medical Faculty Mannheim, Heidelberg University, Theodor-Kutzer-Ufer 1-3, 68167 Mannheim, Germany; Tel.: +49 621 383 2280.
E-mail address: christian.schmid@medma.uni-heidelberg.de (C.D. Schmid).
† These first authors contributed equally to the work: Manuel Winkler, Theresa Staniczek, Maximilian Suhayda
https://doi.org/10.1016/j.jhepr.2025.101475

of periportal liver fibrosis.[8] Our group identified GATA4 as a transcription factor specifically expressed in LSECs (compared to endothelial cells in most other vascular beds). As assessed in *Stab2-cre/Gata4* $^{fl/fl}$ mice with loss of *Gata4* in endothelial cells early in embryonic development, we demonstrated that GATA4 controls embryonic LSEC differentiation and fetal hematopoiesis.[9] Using *Clec4g-iCre/Gata4* $^{fl/fl}$ mice with liver endothelial-specific loss of *Gata4* late in fetal life, we demonstrated that endothelial GATA4 controls metabolic dysfunction-associated steatohepatitis (MASH)-like perisinusoidal liver fibrosis by preventing a pathogenic switch in angiocrine signaling.[10] Notably, in the latter study, endothelial *Maf* expression was significantly downregulated in LSECs from *Clec4g-iCre/Gata4* $^{fl/fl}$ mice, indicating that c-Maf might be a downstream effector of GATA4 that regulates LSEC differentiation and function.

Gómez-Salinero *et al.* have shown that specification of fetal liver endothelial progenitors to functional, zonated adult sinusoids requires c-Maf. However, loss of *Maf* in the inducible *VE-cadherin-CreERT2* model was not accompanied by development of either spontaneous periportal or perisinusoidal liver fibrosis, while carbon tetrachloride-induced periportal liver fibrosis was aggravated.[11]

Here, using *Clec4g-iCre/Maf* $^{fl/fl}$ (*Maf* $^{LSEC\text{-}KO}$) mice with a high penetrance of excision from late fetal life onwards in a LSEC-selective manner, we demonstrate that loss of liver sinusoidal endothelial *Maf* caused MASH-like perisinusoidal liver fibrosis. Perisinusoidal liver fibrosis was driven by sinusoidal capillarization and by a switch towards expression of pro-fibrotic angiokines. Single-cell (sc) RNA sequencing (RNA-seq) analysis of LSECs revealed a fundamental loss of LSEC identity and replacement of normal LSEC subpopulations with capillarized, proliferative, sprouting and secretory hepatic microvascular EC subsets in *Maf* $^{LSEC\text{-}KO}$ mice. Finally, we show that endothelial c-Maf protects from liver fibrosis by controlling LSEC chromatin accessibility, promoting hepatic sinusoidal endothelial *vs.* continuous endothelial identity and suppressing activation of profibrotic and angiogenic endothelial gene programs in the liver.

Materials and methods

Ethical compliance

The experimental protocols used in this study complied with national and international ethical guidelines and, in case of animal models, were approved by the animal welfare commission of the Regierungspraesidium Karlsruhe (Karlsruhe, Germany).

Animal models

Female and male mice aged 3 and 6 months were used in this study. Mice were hosted under specific pathogen-free conditions in single ventilated cages in a 12 h/12 h day/night cycle and fed *ad libitum* with a standard rodent diet (V1534-000, Ssniff) with free access to water.

For the generation of liver sinusoidal endothelial conditional *Maf*-knockout mice, *Clec4g-iCre* mice (C57BL/6N-Tg(*Clec4g*-icre)1.1Sgoe, MGI:6280453)[7] were crossed with *Maf*-floxed mice (B6.129P2-*Maf* $^{tm2.1Cbm}$, MGI:5316775).[12] Mice with the genotype *Clec4g-iCre* $^{tg/wt}$ x *Maf* $^{fl/fl}$ indicating homozygous recombination were denoted as *Maf* $^{LSEC\text{-}KO}$. In all experiments, the littermates with the two genotypes *Clec4g-iCre* $^{wt/wt}$ x *Maf* $^{fl/fl}$ and *Clec4g-iCre* $^{wt/wt}$ x *Maf* $^{fl/wt}$ were used as controls.

Statistical analysis

Statistical analyses were conducted in R 4.1.2 (R Core Team) and Prism 10 (GraphPad Software). For sample size calculation, we suggested an α-level of 0.05 and a β-level of 0.2, while the power was adjusted for each experiment. Welch's *t* test and Mann-Whitney *U* test were used for statistical testing. Two-way ANOVA followed by Tukey's *post hoc* test was used for statistical comparison of more than two groups with two independent variables. A *p* value of <0.05 was considered statistically significant. The appropriate statistical test was chosen according to the requirements of each test (*e.g.* normal distribution). Normal distribution was assessed using the Shapiro-Wilk test.

Additional methodological details

For further information on materials and methods, please refer to the supplementary data.

Results

Endothelial *Maf* deficiency causes perisinusoidal liver fibrosis without affecting metabolic liver zonation

To investigate the role of c-Maf in LSEC differentiation and liver function, we generated a novel mouse model by crossing *Maf*-floxed mice with *Clec4g-iCre* driver mice resulting in *Clec4g-iCre/Maf* $^{fl/fl}$ (*Maf* $^{LSEC\text{-}KO}$) mice. *Maf* $^{LSEC\text{-}KO}$ mice were born in approximately the expected Mendelian ratio and had a normal lifespan (Fig. S1A and B). At 12 weeks of age, body weight, liver weight and liver/body weight ratio were not significantly altered in *Maf* $^{LSEC\text{-}KO}$ mice (Fig. S1C).

Because of *Maf* loss in the liver endothelium (Fig. 1A, upper panel), we detected significantly increased deposition of extracellular matrix proteins, as assessed by Sirius red staining of liver samples, mainly located in midzonal areas of the liver, *i.e.* perisinusoidal liver fibrosis (Fig. 1A, lower panel). Consistent with these observations, immunofluorescence (IF) staining showed significantly increased perivascular deposition of collagens 1 and 4 (COL1A1 and COL4A1) (Figs 1B and S1D). A collagen assay using whole liver lysate confirmed an increase in the amount of collagen in *Maf* $^{LSEC\text{-}KO}$ livers (Fig. 1C). In addition, qPCR analysis of whole liver lysate showed increased *Col1a1* and *Col3a1* expression (Fig. 1D). Notably, IF for SMA and PDGBRB, as well as *in situ* hybridization (ISH) for *Pdgfrb,* showed an increase in the number of activated stellate cells, intricately involved in the development of liver fibrosis (Figs 1E and S1E). The increased number of activated stellate cells was consistent with an increase in *Pdgfrb* expression on qPCR from whole liver lysate (Fig. 1D), while expression of other stellate cell marker genes was not enhanced (Fig. S1F).

To define the cell type of origin for the different collagen subtypes enriched in *Maf* $^{LSEC\text{-}KO}$ livers, namely *Col1a1*, *Col3a1*, and *Col4a1*, we performed FISH (fluorescence ISH) (Fig. S2A–C). We found that *Col1a1* and *Col3a1* are mainly produced by hepatic stellate cells (HSCs), whereas *Col4a1* is mainly derived from ECs (Fig. S2A–B).

Liver enzymes, including alanine aminotransferase, aspartate aminotransferase and glutamate dehydrogenase were slightly elevated in the plasma of *Maf* $^{LSEC\text{-}KO}$ mice indicating

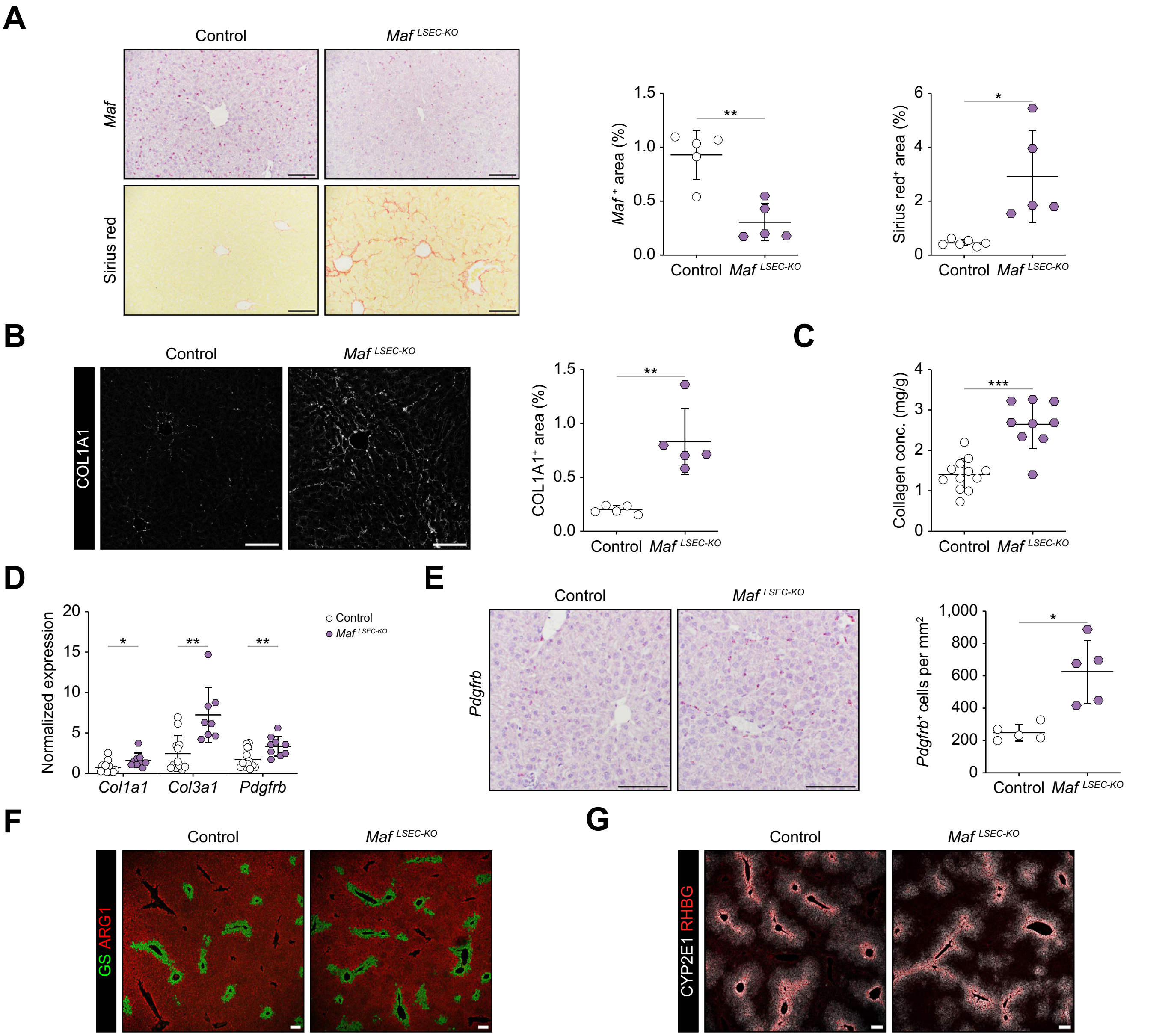

Fig. 1. Endothelial *Maf* deficiency leads to perisinusoidal liver fibrosis while metabolic zonation is preserved. (A) *Maf* ISH and Sirius red staining with quantification (n = 5, 6). (B) Immunofluorescence staining and quantification of COL1A1 (n = 5). (C) Tissue collagen assay of livers (n = 9, 12). (D) qPCR for *Col1a1*, *Col3a1* and *Pdgfrb* using RNA from whole liver (n = 8). (E) *Pdgfrb* ISH in mouse livers (n = 5). (F) Immunofluorescence staining of GS and ARG1 (n = 8); and (G) CYP2E1 and RHBG in livers (n = 5). Scale bars: 100 µm. Mean ± SD. (A-E) Welch's *t* test; **p* <0.05; ***p* <0.01; ****p* <0.001. ISH, *in situ* hybridization.

moderate hepatopathy, which is often associated with perisinusoidal liver fibrosis, while other standard plasma values were normal (Fig. S3A). Immunofluorescent analysis using F4/80 and CD11b antibodies showed a significant increase in monocyte/macrophage numbers in $Maf^{LSEC\text{-}KO}$ livers, indicating hepatic inflammation, which often accompanies and aggravates liver fibrosis (Fig. S3B).

On the contrary, further staining including H&E, Oil red O and Prussian blue, as well as a triglyceride assay on whole liver lysate, did not reveal hepatic steatosis or iron deposition in $Maf^{LSEC\text{-}KO}$ livers (Fig. S3C and D). As *Maf* is known to be mainly expressed in midzonal LSECs, it was not unexpected that metabolic liver zonation, which is controlled by pericentral angiocrine factors, was also normal, as shown by immunofluorescence staining for GS, ARG1, CYP2E1, and RHBG (Figs 1F,G and S3E).

Endothelial *Maf* deficiency leads to a shift from discontinuous to continuous marker expression, entailing sinusoidal capillarization, and to upregulation of profibrotic angiocrine factors

When analyzing LSEC differentiation in $Maf^{LSEC\text{-}KO}$ mice *in situ*, we detected a shift from discontinuous to continuous endothelial marker expression. IF staining showed that while LYVE1, CD32, and STAB2 were significantly downregulated, EMCN

and CD31 were significantly upregulated along the whole length of the liver sinuses (Fig. 2A).

Interestingly, immunofluorescent staining for Ki-67 and pan-endothelial marker PODXL showed significantly increased cell proliferation of LSECs in *Maf*$^{LSEC-KO}$ mice (Fig. 2B).

To examine the ultrastructural changes in LSECs upon *Maf* deficiency, we performed transmission electron microscopy (Figs 2C and S4A). LSECs in *Maf*$^{LSEC-KO}$ mice were lined by a basement membrane, and fibrous collagen deposition was seen in the space of Disse (Fig. 2C), which is the ultrastructural

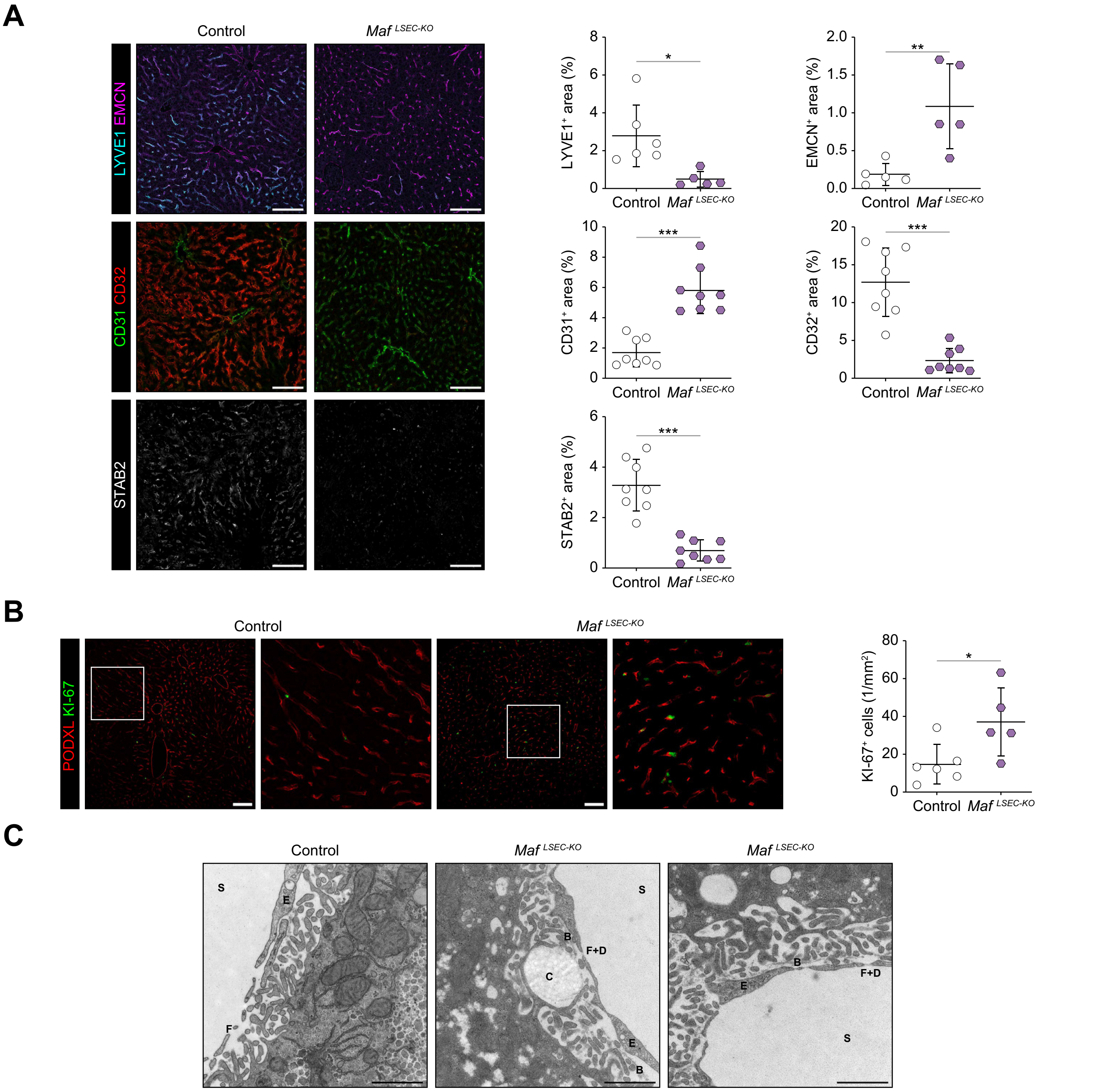

Fig. 2. Upregulation of continuous endothelial marker genes and proliferation marker Ki-67 in LSECs from *Maf*$^{LSEC-KO}$ mice. (A) Immunofluorescence staining and quantification of LYVE1, EMCN, CD31, CD32, and STAB2 in livers (n = 5, 6, 8). (B) Immunofluorescence staining for Ki-67 and pan-endothelial marker PODXL and Ki-67 quantification (n = 5, 6). (C) TEM of livers (S = sinusoidal lumen, E = endothelial cell, F = fenestration, D = diaphragm, B = basement membrane, C = collagen fiber) (n = 1, 2). (A-B) Scale bars: 100 µm; (C) scale bars: 1 µm. Mean ± SD. (A [LYVE1, EMCN, STAB2], B) Welch's *t* test; (A [CD31, CD32]) Mann-Whitney *U* test; **p* <0.05; ***p* <0.01; ****p* <0.001. LSECs, liver sinusoidal endothelial cells; TEM, transmission electron microscopy.

correlate of the perisinusoidal liver fibrosis described above (Fig. 1A). In addition, *Maf* $^{LSEC\text{-}KO}$ LSECs showed fenestrae that were covered by a diaphragm (Fig. 2C). Thus, our transmission electron microscopy results confirm development of sinusoidal capillarization not only at the marker level, but also at the ultrastructural level.

In addition to sinusoidal capillarization, the known profibrotic angiocrine factors *Esm1*, *Sparcl1*, *Igfbp5,* and *Pdgfb* were significantly upregulated in LSECs in *Maf* $^{LSEC\text{-}KO}$ livers as seen by ISH, likely contributing to a profibrotic milieu around the hepatic sinus (Fig. 3A). Endothelial *Pdgfb* expression was confirmed using *Cd34-Pdgfb* FISH (Fig. 3B).

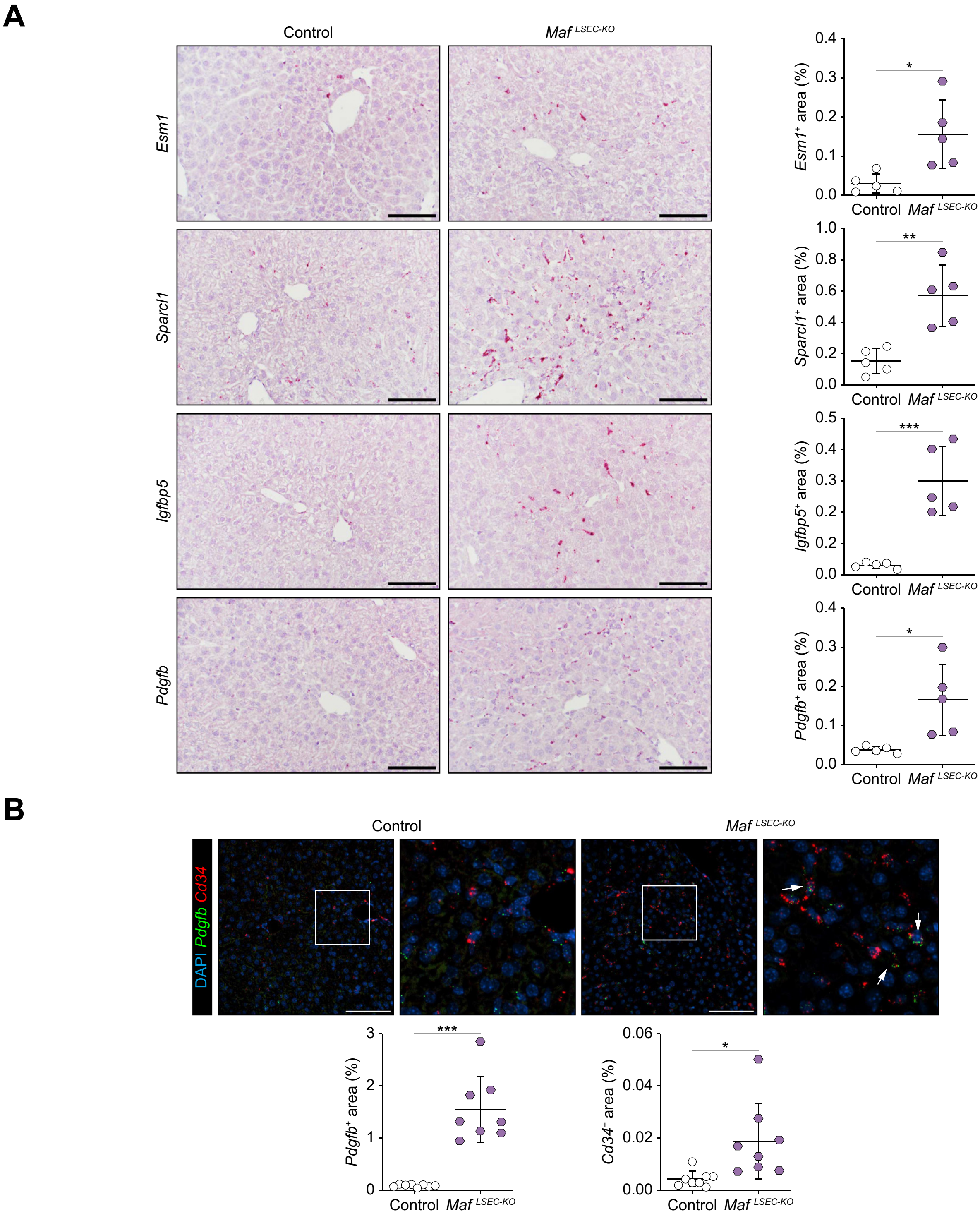

Fig. 3. Endothelial *Maf* deficiency causes increased expression of profibrotic angiocrine factors. (A) ISH and quantification for *Esm1, Sparcl1, Igfbp5,* and *Pdgfb* of livers (n = 5). (B) FISH and quantification for *Pdgfb* and *Cd34* of livers (n = 8). Scale bars: 100 µm. Mean ± SD. (A [*Igfbp5, Pdgfb*], B [*Pdgfb*]) Welch's *t* test; (A [*Esm1, Sparcl1*], B [*Cd34*]) Mann-Whitney *U* test; $^{*}p$ <0.05; $^{**}p$ <0.01; $^{***}p$ <0.001. FISH, fluorescence ISH; ISH, *in situ* hybridization.

Loss of endothelial *Maf* aggravates liver fibrosis in a dietary model of MASH

To investigate whether endothelial *Maf* deficiency aggravates liver injury in a dietary MASH model with late development of liver fibrosis, we fed *Maf* $^{LSEC\text{-}KO}$ and control mice a choline-deficient, L-amino acid-defined (CDAA) diet for 10 weeks. Basic animal data such as body weight and liver weight, were not significantly altered while liver/body weight ratio was slightly increased in CDAA-fed *vs*. Chow-fed control animals (Fig. S4B). On the contrary, livers from *Maf* $^{LSEC\text{-}KO}$ mice fed a CDAA diet did not only show significantly increased extracellular matrix deposition on Sirius red staining compared to CDAA-fed control animals, but also compared to Chow-fed *Maf* $^{LSEC\text{-}KO}$ mice (Fig. 4A). The increased susceptibility of *Maf* $^{LSEC\text{-}KO}$ livers to MASH-related injury was also reflected by significantly increased levels of hepatocyte damage marker glutamate dehydrogenase in the peripheral blood of CDAA-fed *Maf* $^{LSEC\text{-}KO}$ mice compared to both CDAA-fed control animals and to Chow-fed *Maf* $^{LSEC\text{-}KO}$ mice, while alanine and aspartate aminotransferase showed no significant differences between CDAA-fed control animals and CDAA-fed *Maf* $^{LSEC\text{-}KO}$ mice (Fig. 4B).

IF indicated that the level of sinusoidal capillarization of LSECs in *Maf* $^{LSEC\text{-}KO}$ mice was not further enhanced by CDAA diet feeding, as CD31 upregulation and CD32 loss were not significantly different between Chow-fed and CDAA-fed *Maf* $^{LSEC\text{-}KO}$ mice, indicating that endothelial *Maf* deficiency *per se* caused a maximal effect (Fig. 4C). Furthermore, metabolic zonation of the liver was conserved upon CDAA diet feeding in both *Maf* $^{LSEC\text{-}KO}$ and control livers as illustrated by pericentral markers CYP2E1 and GS, as well as periportal markers ARG1 and HAL (Figs 4D,E and S4C,-D).

In conclusion, dietary induction of MASH aggravated the degree of liver fibrosis caused by endothelial *Maf* deficiency in the liver. However, most other parameters analyzed in our basic characterization remained unaffected. Therefore, further experiments were performed exclusively with Chow-fed *Maf* $^{LSEC\text{-}KO}$ and control mice.

Increased endothelial cell proliferation and loss of LSEC zonation in *Maf* $^{LSEC\text{-}KO}$ mice

To further analyze the changes in hepatic microvascular endothelial differentiation, bulk RNA-seq was performed using LSECs from *Maf* $^{LSEC\text{-}KO}$ *vs.* control mice isolated by gradient centrifugation and positive selection for CD146 antigen expression (Table S1, Fig. S5A and B). Strictly speaking, these cells also include ECs from large hepatic blood vessels, *e.g.* central veins and portal arteries, but because LSECs represent by far the largest EC population in the liver[13] and for readability, we refer to these cells as "LSECs".

RNA-seq confirmed the effective knockout of *Maf* in LSECs (Fig. S5C). To identify critical genes that promote the phenotype seen upon *Maf* deficiency in LSECs, we focused on the top differentially expressed genes in *Maf* $^{LSEC\text{-}KO}$ LSECs.

Among the most strongly upregulated genes, we identified the continuous endothelium marker gene *Cd34* and the proliferation marker gene *Mki67* (Fig. 5A) confirming our IF data (Figs 2B and 3B). Interestingly, *Ly6c1* was the most strongly upregulated gene, a gene primarily known as a monocyte/macrophage marker. However, LY6C IF confirmed the endothelial expression of LY6C especially in *Maf* $^{LSEC\text{-}KO}$ livers (Fig. S6A). As *Ly6c1* has been described to be expressed by ECs in various vascular beds, induced endothelial expression of *Ly6c1* in *Maf* $^{LSEC\text{-}KO}$ livers may be part of the sinusoidal-to-continuous endothelial dedifferentiation program.[14,15]

Among the transcription factors, *Myc* was the most strongly upregulated in LSECs from *Maf* $^{LSEC\text{-}KO}$ mice (Figs 5B and S6B). In addition, the transcriptomic data confirmed the upregulation of the profibrotic factors *Igfbpb5* and *Pdgfb* (Fig. 5C). Upregulation of these genes is consistent with LSEC dedifferentiation and is congruent with the increased proliferative and profibrotic capacity of LSECs due to *Maf* deficiency.

Notably, cell adhesion and extracellular matrix-interacting molecule *Flrt2* and *Cxcl12* were identified as novel candidate molecules that may contribute to liver fibrogenesis in *Maf* $^{LSEC\text{-}KO}$ mice (Fig. 5A,C). Significant upregulation of *Flrt2*, was confirmed by ISH and qPCR (Figs S6C-E and S7A). *Cxcl12* was mostly expressed by cells other than LSECs, but there was no significant difference between the genotypes, albeit a trend towards higher expression in *Maf* $^{LSEC\text{-}KO}$ livers (Fig. S6D and E).

Interestingly, *Wnt9b*, *Rspo3,* and *Wnt2* were significantly downregulated in our bulk RNA-seq data of *Maf*-deficient LSECs (Fig. 5C), while we did not see significant changes by ISH (Fig. S7B). Similarly, *Bmp2* and *Hgf* were not significantly altered on ISH. However, ISH is not as sensitive as RNA-seq, a difference which may explain why results for these genes in ISH did not confirm differential expression. In addition, *Wnt9b*, *Rspo3,* and *Wnt2* are preferentially expressed by central vein ECs and pericentral LSECs. Preservation of a certain gradient and level of expression of Wnt signaling molecules in LSECs may also explain preserved metabolic liver zonation in *Maf* $^{LSEC\text{-}KO}$ mice (Fig. 1F,G), especially since *Maf* expression is lower in pericentral LSECs and *Maf* excision in pericentral LSECs is less effective compared to midzonal LSECs (see below).

Gene ontology analysis of transcripts altered in LSECs isolated from *Maf* $^{LSEC\text{-}KO}$ mice further revealed significant upregulation of genes associated with cell proliferation, including chromosome segregation, nuclear division and mitotic cell cycle transition (Fig. 5D), while the downregulated genes were associated with immunological functions including activation of immune response, leukocyte-mediated immunity, and regulation of immune effector processes (Fig. 5E). The downregulation of immunological gene sets in LSECs from *Maf* $^{LSEC\text{-}KO}$ mice, even though we observed more monocytes/macrophages in *Maf* $^{LSEC\text{-}KO}$ liver tissue (Fig. S3B) that could have contaminated our LSEC preparations, could be due to the capillarization of LSECs in our *Maf* $^{LSEC\text{-}KO}$ mice, since continuous ECs generally express fewer immunological molecules than healthy LSECs.[16]

Enrichment analysis to distinguish tip cells from stalk cells[17] indicated a slight preponderance of tip cell markers among the genes upregulated in LSECs isolated from *Maf* $^{LSEC\text{-}KO}$ mice, indicating ongoing angiogenesis (Fig. S8A).

Gene set-enrichment analysis of LSEC marker gene sets revealed an increase in the expression of portal vein endothelial genes in *Maf* $^{LSEC\text{-}KO}$ LSECs, while specific periportal, midzonal, and pericentral LSEC transcripts were reduced (Fig. 5F). This finding confirms a change in differentiation of LSECs towards the differentiation of continuous ECs accompanied by loss of sinusoidal zonation in *Maf* $^{LSEC\text{-}KO}$ mice. On the contrary, central vein transcripts were significantly less affected in *Maf* $^{LSEC\text{-}KO}$ mice (Fig. 5F). In addition, lymphatic EC markers were slightly increased (Fig. S8B).

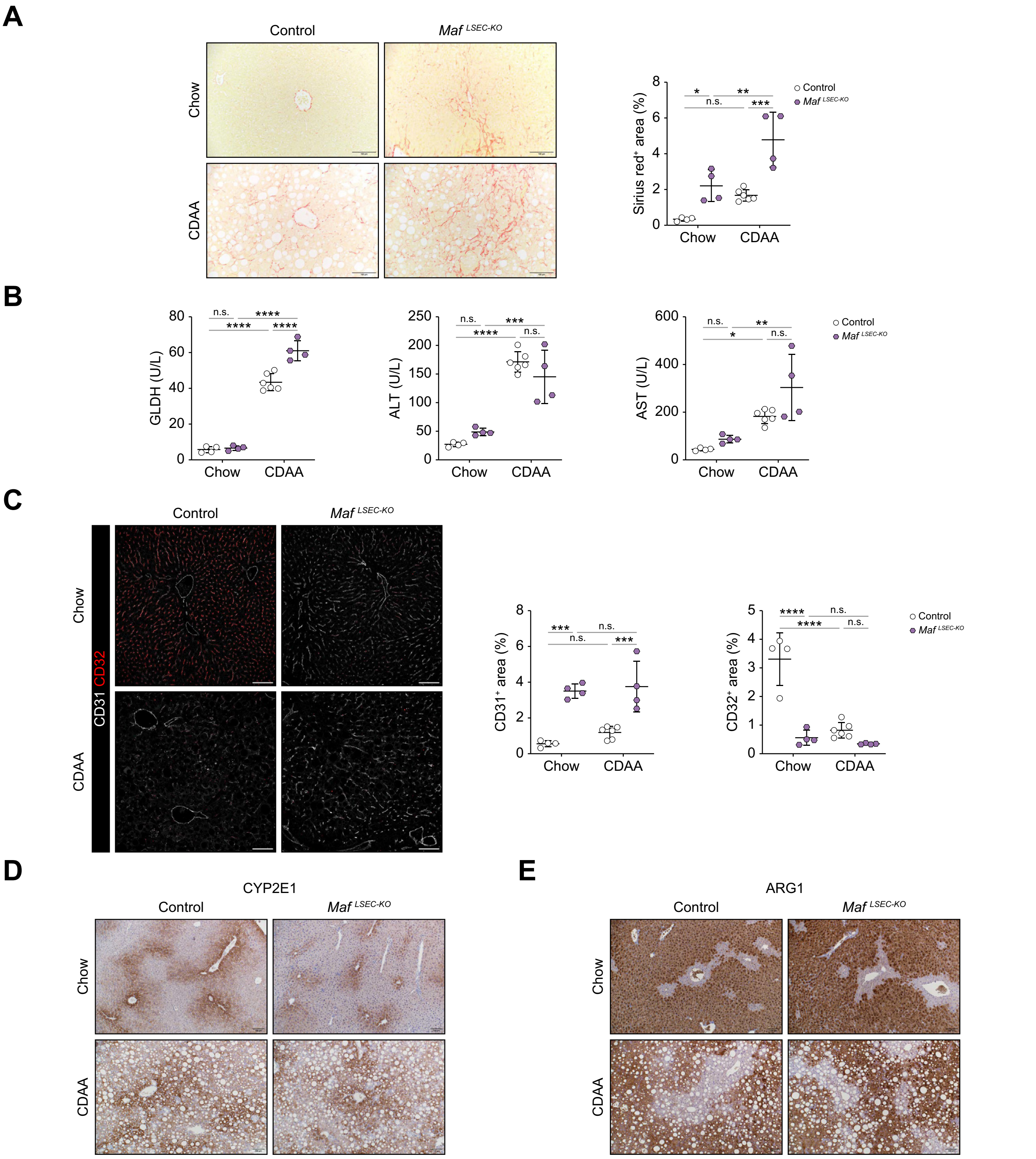

Fig. 4. ***Maf*** **knockout aggravates MASH diet-induced liver fibrosis.** (A) Sirius red staining and quantification of control and *Maf*$^{LSEC-KO}$ livers after Chow or CDAA diet for 10 weeks (n = 4, 6). (B) Blood plasma levels of GLDH, ALT, and AST in control and *Maf*$^{LSEC-KO}$ mice (n = 4, 6). (C) Immunofluorescence staining and quantification for CD31 and CD32 (n = 4, 6). (D) CYP2E1 and (E) ARG1 Immunohistochemistry staining of control and *Maf*$^{LSEC-KO}$ livers after Chow or CDAA diet for 10 weeks (n = 4, 6). Scale bars: 100 μm. (A-C) two-way ANOVA, Tukey's *post hoc* test; n.s. *p* ≥0.05; **p* <0.05; ***p* <0.01; ****p* <0.001.; *****p* <0.0001. ALT, alanine aminotransferase; AST, aspartate aminotransferase; CDAA, choline-deficient, L-amino acid-defined; GLDH, glutamate dehydrogenase; LSECs, liver sinusoidal endothelial cells; MASH, metabolic dysfunction-associated steatohepatitis.

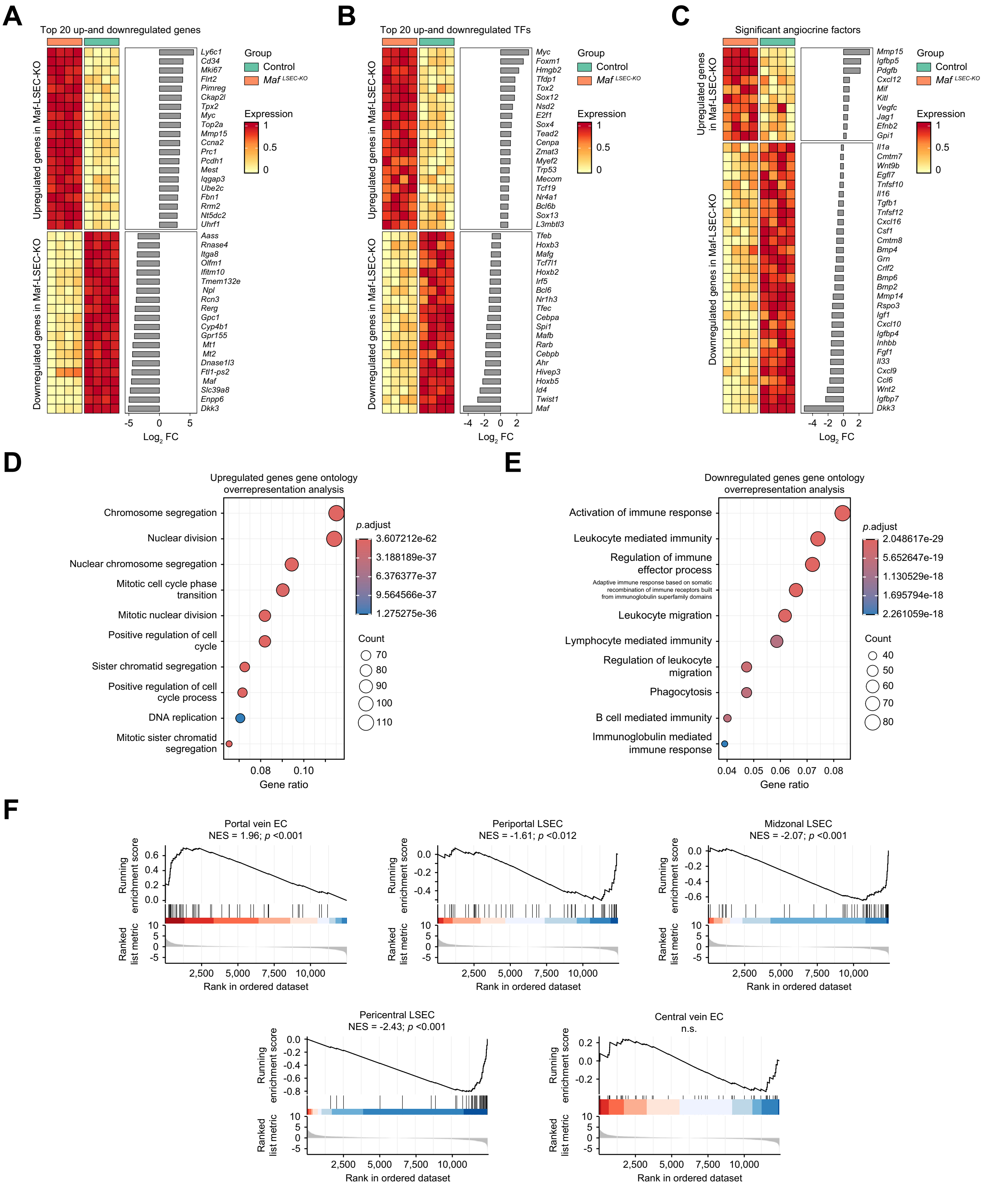

Fig. 5. Dysregulation of transcription factors and angiocrine factors with a shift towards portal vein transcripts in *Maf*-deficient LSECs. Heat maps of (A) top significantly up- and downregulated genes, (B) transcription factors, and (C) angiocrine factors in LSECs. Gene ontology overrepresentation analyses of (D) upregulated and (E) downregulated genes. (F) Enrichment plots of portal vein, periportal, midzonal, pericentral, and central vein associated genes. NES, normalized enrichment score (n = 4). LSECs, liver sinusoidal endothelial cells.

Endothelial *Maf* deficiency causes dysregulated chromatin accessibility promoting counter-regulation of genes physiologically expressed or silenced in LSECs

To better understand the role of c-Maf in orchestrating LSEC gene expression, we characterized the chromatin landscape of LSECs isolated from control and $Maf^{LSEC-KO}$ mice by ATAC-seq (assay for transposase-accessible chromatin with sequencing). In agreement with the bulk RNA-seq analyses (Fig. 5), *Maf* deficiency led to broad changes in the chromatin accessibility of LSECs, with over 10,000 differentially accessible regions in LSECs from $Maf^{LSEC-KO}$ mice (Fig. 6A, Table S2), including 6,454 sites with increased accessibility and 4,713 sites with decreased accessibility. Overall, we observed a high degree of concordance between the changes observed at the chromatin accessibility and gene expression levels (Fig. 6B).

In addition, we performed TOBIAS footprinting analysis[18] using our ATAC-seq data to identify transcription factors that differentially bind to the genome upon *Maf* deficiency in LSECs. Reassuringly, the TOBIAS results revealed a cluster of MAF transcription factors with markedly reduced activity in $Maf^{LSEC-KO}$ LSECs, likely reflecting lack of c-Maf. Moreover, this analysis indicated reduced binding by the architectural transcription factor CTCF, whereas transcription factors such as FOS and JUN, which have been reported to form heterodimers with c-Maf,[19] were more active and showed more genomic binding (Fig. 6C, Table S3).

Next, we focused on differential c-Maf footprints – unbound in the *Maf*-knockout LSECs and bound in controls – to identify direct transcriptional targets of c-Maf. After filtering for highly expressed genes (>50th expression percentile in RNA-seq data), we identified 293 genes that were significantly less bound by c-Maf in the *Maf*-knockout LSECs. These genes were intersected with the significantly differentially expressed genes from our RNA-seq data to identify genes in which c-Maf shows direct transcriptional relevance (Fig. 6D).

A total of 144 genes from the differentially less bound list were also found among the significantly downregulated genes in RNA-seq, while only 44 genes overlapped with the significantly upregulated genes (Fig. 6D, Table S4). Among the genes directly positively regulated by c-Maf, we identified *Enpp6*, *Dnase1l3*, *Aass*, *Stab2*, *Lyve1*, and *Wnt2* (Fig. 6E-F). While the same analysis indicated that c-Maf is a direct negative regulator of *Nid2*, *Samd4*, and *Mindy4* (Fig. 6E).

Endothelial *Maf* deficiency causes replacement of zonated LSEC subpopulations with capillarized, profibrotic and angiogenic endothelial cell subsets

To investigate whether endothelial *Maf* deficiency alters the composition and zonated gene expression profiles of LSEC subpopulations, we performed scRNA-seq on isolated LSECs from control and $Maf^{LSEC-KO}$ mice followed by bioinformatic filtering to exclude contaminating ECs from large blood vessels, immune cells, stellate cells, and hepatocytes (*Adgre1*-, *Clec4f*-, *Ptprc*-, *Cd52*-, *Acta1*-, *Arg1*-, *Pecam1*+, *Vwf*-). We merged the filtered single-cell data from the different samples, *i.e.* $Maf^{LSEC-KO}$ and controls, to investigate differences between the respective LSEC populations.

The uniform manifold approximation and projection plot of the merged data showed an almost complete separation between control and $Maf^{LSEC-KO}$ LSECs (Fig. 7A). Notably, *Maf*-expressing LSECs from $Maf^{LSEC-KO}$ mice clustered together with LSECs from control animals (Fig. 7A). Clustering analysis revealed seven distinct clusters: periportal, midlobular, and pericentral subclusters – predominantly found in control LSECs – and capillarized, proliferative, sprouting, and secreting subclusters, which were predominantly observed in $Maf^{LSEC-KO}$ LSECs (Fig. 7B-D).

The continuous EC genes *Cd34* and *Pdgfb* were almost exclusively expressed by ECs from $Maf^{LSEC-KO}$ mice and *Cd34+* as well as *Pdgfb+* cells were homogeneously distributed throughout all the $Maf^{LSEC-KO}$ LSEC subclusters (Figs 7E and S9A), indicating a general change in differentiation from normal LSECs to continuous profibrotic ECs. *Mki67* – already identified by bulk RNA-seq analysis (Fig. 5A) – labelled the proliferative cluster (Figs 7D,E and S9A). Similarly, the secreting subcluster in $Maf^{LSEC-KO}$ LSECs was labelled by *Sparcl1* (Fig. 7D). Notably, the secreting cluster also showed expression of angiogenic chemokines like *Cxcl12* (Figs 7D,E and S9A). In addition, we analyzed expression of the profibrotic and angiogenic factors *Igfbp5* and *Flrt2* previously identified in our bulk RNA-seq analysis (Fig. 5A,C) and could show that they were preferentially expressed in the capillarized subcluster (Figs 7E and S9A). Using our scRNA-seq data, we again confirmed the endothelial expression of *Ly6c1* and *Mmp15,* which was previously identified by bulk-RNA-seq of LSECs in $Maf^{LSEC-KO}$ livers (Figs 7E and S9A).

To analyze LSEC zonation in control and $Maf^{LSEC-KO}$ mice, we developed a zonation score based on the expression ratio of known periportal and pericentral LSEC genes as described in detail in supplementary materials and methods. This analysis demonstrated disruption of physiological LSEC zonation in $Maf^{LSEC-KO}$ mice (Fig. 7F,G) confirming the shift in endothelial phenotype towards periportal LSECs seen in our bulk RNA-seq data (Fig. 5F).

In addition, scRNA-seq data from LSECs confirmed the previous results that *Col4a1* is produced by ECs, whereas *Col1a1* and *Col3a1* must originate from other hepatic cells, most likely HSCs (Fig. S10A and B).

We also used our scRNA-seq data to investigate the zonated expression of Wnt factors. Interestingly, the Wnt factors *Wnt2*, *Wnt9b,* and *Rspo3* were highly downregulated in $Maf^{LSEC-KO}$ LSECs, while a gradient of low expression levels was conserved, with higher expression in pericentral areas compared to periportal areas (Fig. S10C). This may be due to lower *Maf* expression in pericentral LSECs, and the likely reduced efficiency of *Maf* excision in these cells owing to lower *Clec4g* expression compared to midlobular LSECs (Fig. S10C). This may explain why we see conserved metabolic zonation in our $Maf^{LSEC-KO}$ model (Fig. 1F,G), despite the significant loss of Wnt factors in our sequencing data from isolated LSECs.

Profibrotic factors FLRT2 and CXCL12 activate HSCs *in vitro*

To investigate the functional role of the identified angiocrine factors *Pdgfb*, *Igfbp5*, *Flrt2* and *Cxcl12*, we stimulated the human HSC line LX-2 with the respective angiocrine factors *in vitro*. Notably, FLRT2 and CXCL12, but not IGFBP5 and PDGF-BB, significantly induced *COL1A1* expression in LX-2 cells, indicating HSC activation (Fig. 7H). These results highlight the profibrotic role of angiocrine factors FLRT2 and CXCL12 in liver fibrogenesis.

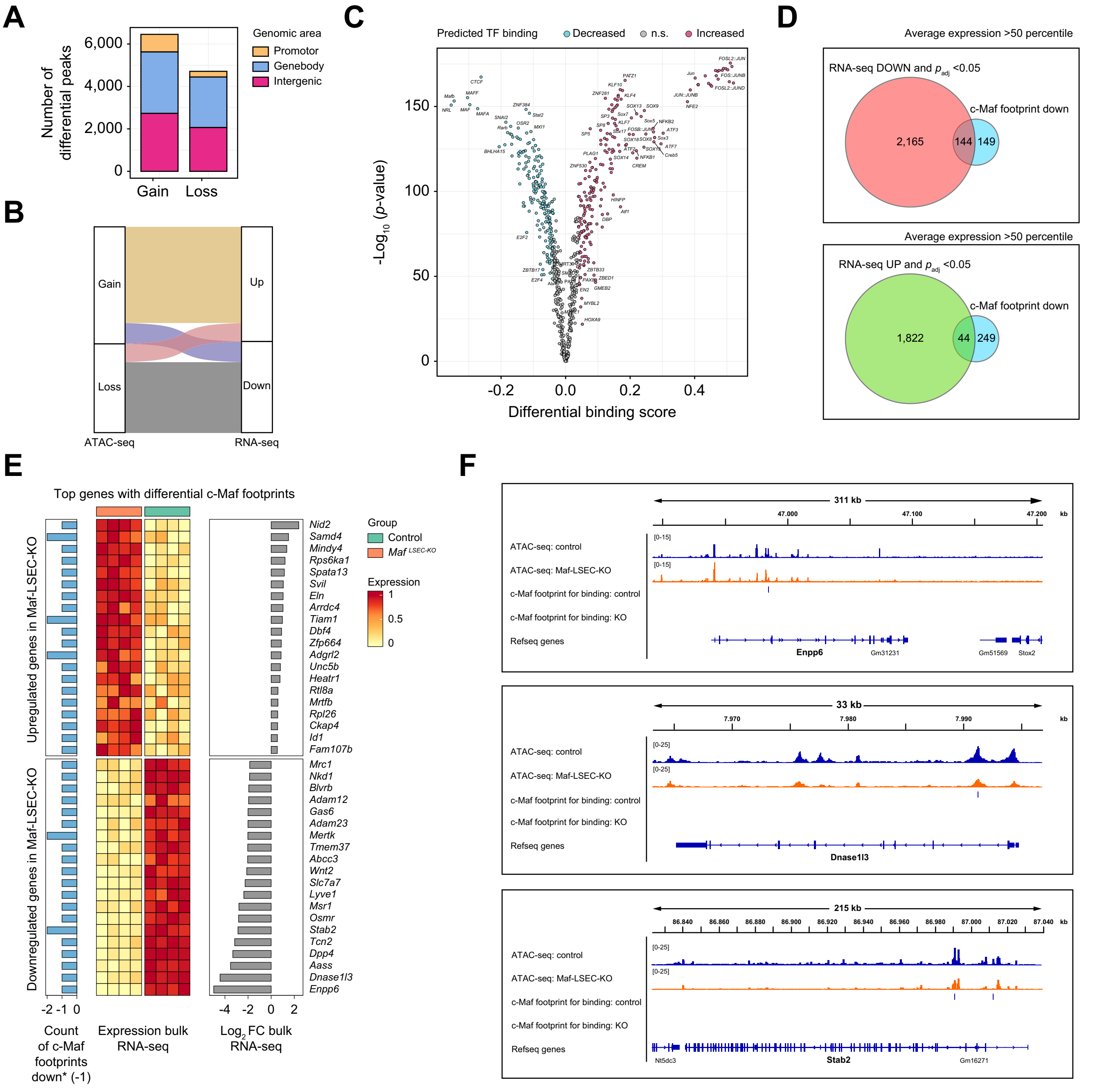

Fig. 6. Loss of endothelial *Maf* leads to major alterations in chromatin accessibility. (A) Bar plot of differentially accessible chromatin areas at promotor, genebody, and intergenic regions in LSECs upon *Maf* deficiency. (B) Alluvial plot illustrating concordance of ATAC-seq and RNA-seq data. (C) Volcano plot of TOBIAS footprinting analysis of ATAC-seq data from LSECs. (D) Venn diagrams of genes showing loss of c-Maf footprints and significant dysregulation at the RNA level. (E) Heatmap of the 20 top up- and downregulated genes at the RNA level with loss of c-Maf footprints. (F) Example gene tracks for selected genes *Enpp6*, *Dnase1l3*, and *Stab2* with annotation for ATAC-seq signal and c-Maf footprints per genotype. ATAC-seq, assay for transposase-accessible chromatin with sequencing; LSECs, liver sinusoidal endothelial cells; RNA-seq, RNA sequencing.

MAF expression is significantly reduced in ECs of human cirrhotic liver

Transcription factors other than c-Maf that determine LSEC differentiation, such as *GATA4* or *ERG*, are known to be downregulated in human patients with liver fibrosis and cirrhosis.[8,10] To elucidate whether the expression of transcription factor *MAF* is also altered in human cirrhosis, we analyzed published scRNA-seq data from healthy and cirrhotic human liver samples.[20] Raw count data was re-analyzed in house (see Methods) to prioritize the resolution of endothelial and zone-specific LSEC clusters. The data was filtered for non-parenchymal cells, enabling the identification of clusters

corresponding to distinct hepatic endothelial subpopulations, including pericentral, midzonal and periportal LSECs, as well as lymphatic, central vein and portal vein ECs (Fig. 8A). Consistent with the original publication,[20] we detected three clusters consisting of cirrhosis-specific ECs, while clusters corresponding to all LSEC zones and central vein ECs were depleted in cirrhotic livers (Fig. 8A). Portal vein ECs retained roughly equal contribution in cells from healthy and cirrhotic livers, suggesting that the loss of characteristic gene expression in cirrhotic ECs is specific to the sinusoidal and central zones (Fig. 8B).

MAF expression was almost exclusively seen in healthy LSECs and to a lesser extent in central vein ECs, lymphatic ECs and a small cluster of cirrhosis-specific mesothelial cells (Fig. 8C). Notably, at a global level, *MAF* expression was significantly reduced in ECs from donors with cirrhosis (Fig. 8D), while it was preserved in the few LSECs that retained a pericentral, midzonal, or periportal expression profile (Fig. 8E).

We further explored the expression of profibrotic and angiogenic factors that our analyses have implicated as mediators of the role of c-Maf in mouse LSECs (Fig. 5A, 5C). While *IGFBP5* was markedly elevated in cirrhotic ECs compared to LSECs (adjusted *p* value = 3.93e-56, $\log_2$ fold change = 1.62) (Fig. 8F), the expression of *PDGFB* and *FLRT2* was similar between healthy and cirrhotic ECs (Fig. 8G,H). These results show that the loss of endothelial *MAF* expression in human liver cirrhosis partially parallels the findings in *Maf*$^{LSEC\text{-}KO}$ mice.

Discussion

In this study, we show that the transcription factor c-Maf, expressed by normal LSECs, helps protect the liver against MASH-like perisinusoidal liver fibrosis by maintaining LSEC identity. c-Maf-dependent LSEC identity encompasses their zonation along the hepatic sinusoids, from pericentral

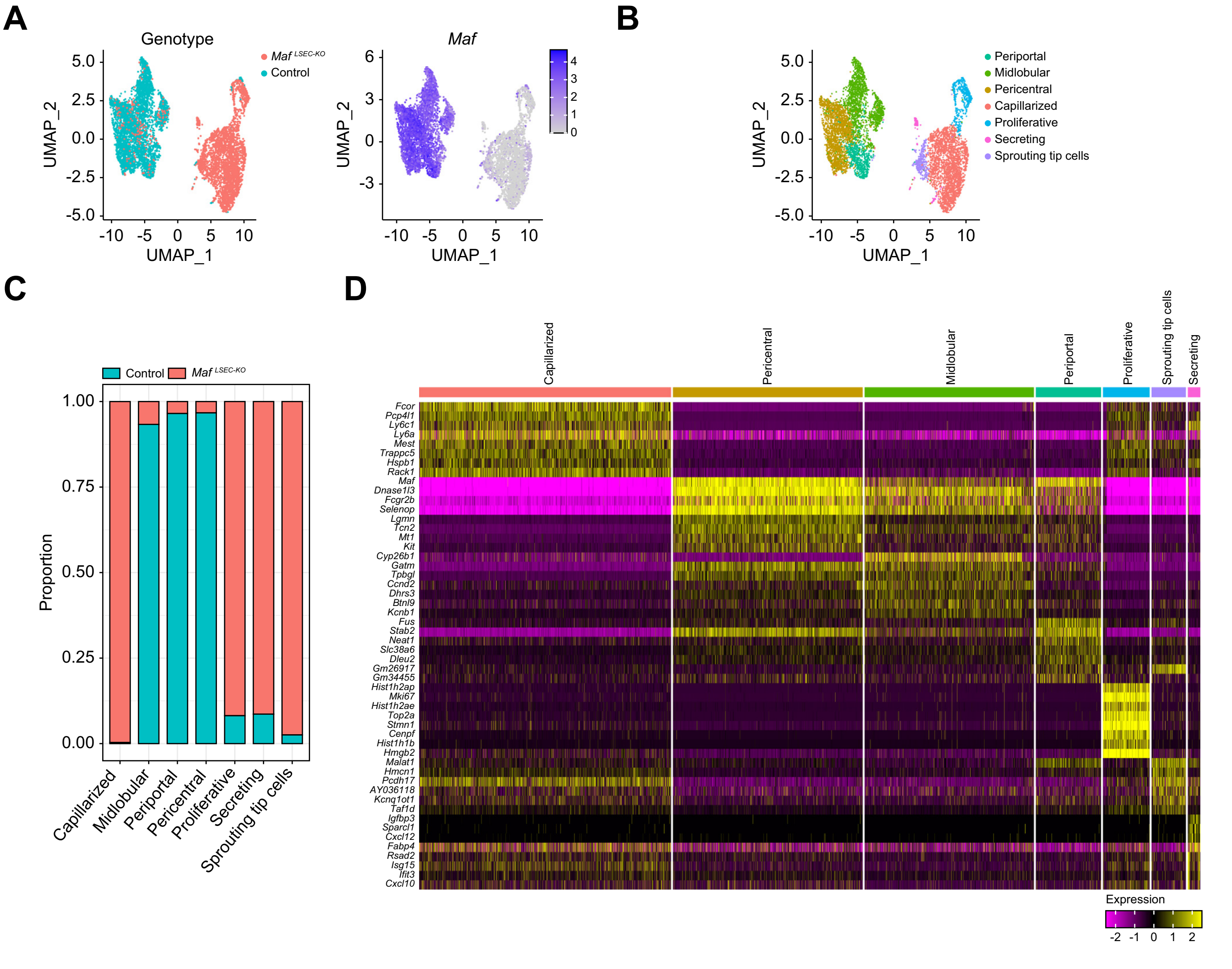

Fig. 7. Identification of novel cell clusters in LSECs upon endothelial *Maf* deficiency. (A) Genotype annotation in UMAP plot of scRNA-seq from control and *Maf*$^{LSEC\text{-}KO}$ LSECs. (B) Annotation for identified clusters in scRNA-seq data. (C) Plot of cell cluster proportions per genotype. (D) Heatmap of identified clusters and their marker genes. (E) Annotation for the genes of interest (*Cd34*, *Pdgfb*, *Mki67*, *Cxcl12, Igfbp5*, *Flrt2, Ly6c1,* and *Mmp15*) in UMAP plot. (F) Violin plot of LSEC zonation scores per genotype (0 corresponds pericentral, 1 corresponds periportal). (G) Annotation for the zonation score quartiles per genotype in UMAP plot. (H) qPCR for *COL1A1* using LX-2 cell RNA after stimulation with CXCL12, FLRT2, IGFBP5 and PDGF-BB (n = 5). (H [CXCL12, FLRT2, IGFBP5]) unpaired *t* test; (H [*PDGF-BB*]) Mann-Whitney *U* test; n.s. *p* >0.05; ***p* <0.01; ****p* <0.001. LSECs, liver sinusoidal endothelial cells; scRNA-seq, single-cell RNA sequencing; UMAP, uniform manifold approximation and projection.

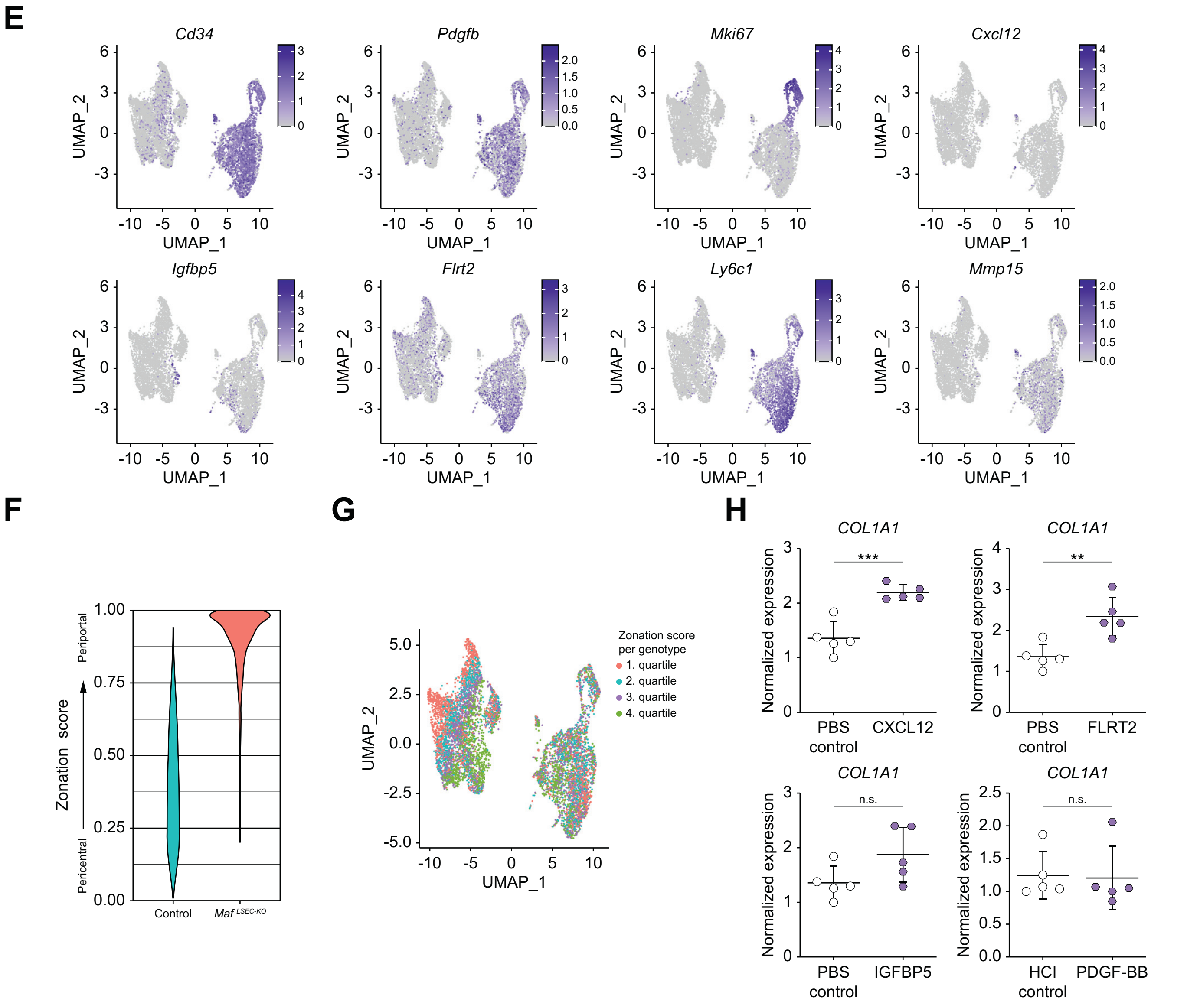

Fig. 7. ***(Continued).***

to periportal regions. The effects of c-Maf in LSECs are mediated by widespread changes in chromatin accessibility, as evidenced by the high number of differentially accessible sites (over 10K). The direct and indirect effects of c-Maf in LSECs, as deduced from TOBIAS footprinting predictions, provide mechanistic insight into how dedifferentiation of LSECs and subsequent functional changes in the hepatic vascular niche may cause MASH-like perisinusoidal liver fibrosis.

Notably, hepatic endothelial c-Maf deficiency allowed for expression of continuous EC genes in liver sinusoids at the expense of sinusoidal EC-specific differentiation and was accompanied by disruption of endothelial zonation along the liver sinusoids. The known profibrotic angiocrine stellate cell activators *Pdgfb* and *Igfbp5* were increased in *c-Maf*-deficient LSECs. *Pdgfb* overexpression alone has been reported to induce liver fibrosis,[21] while IGFBP5 is known to promote the survival of activated HSCs and myofibroblasts and to increase the expression of profibrotic genes, thereby contributing to liver fibrosis.[22]

Furthermore, we identified *Flrt2* and *Cxcl12* as potential drivers of sinusoidal capillarization and we could show *in vitro* that FLRT2 and CXCL12 contribute to HSC activation by inducing *COL1A1* expression. FLRT2 binds to latrophilin-2 thereby promoting tight junction assembly accompanied by reduced vascular permeability.[23] In colorectal cancer, FLRT2 formed noncanonical inter-endothelial adhesions that safeguarded against oxidative stress through homophilic binding.[24] Moreover, FLRT2 has been shown to prevent premature senescence and vascular ageing in endothelial cells upon exposure to risk factors for vascular diseases,[25] indicating that expression of *Flrt2* may be part of a reactive program to protect the hepatic vasculature from hepatic injury and/or promote healing – a process that requires activation of HSCs and

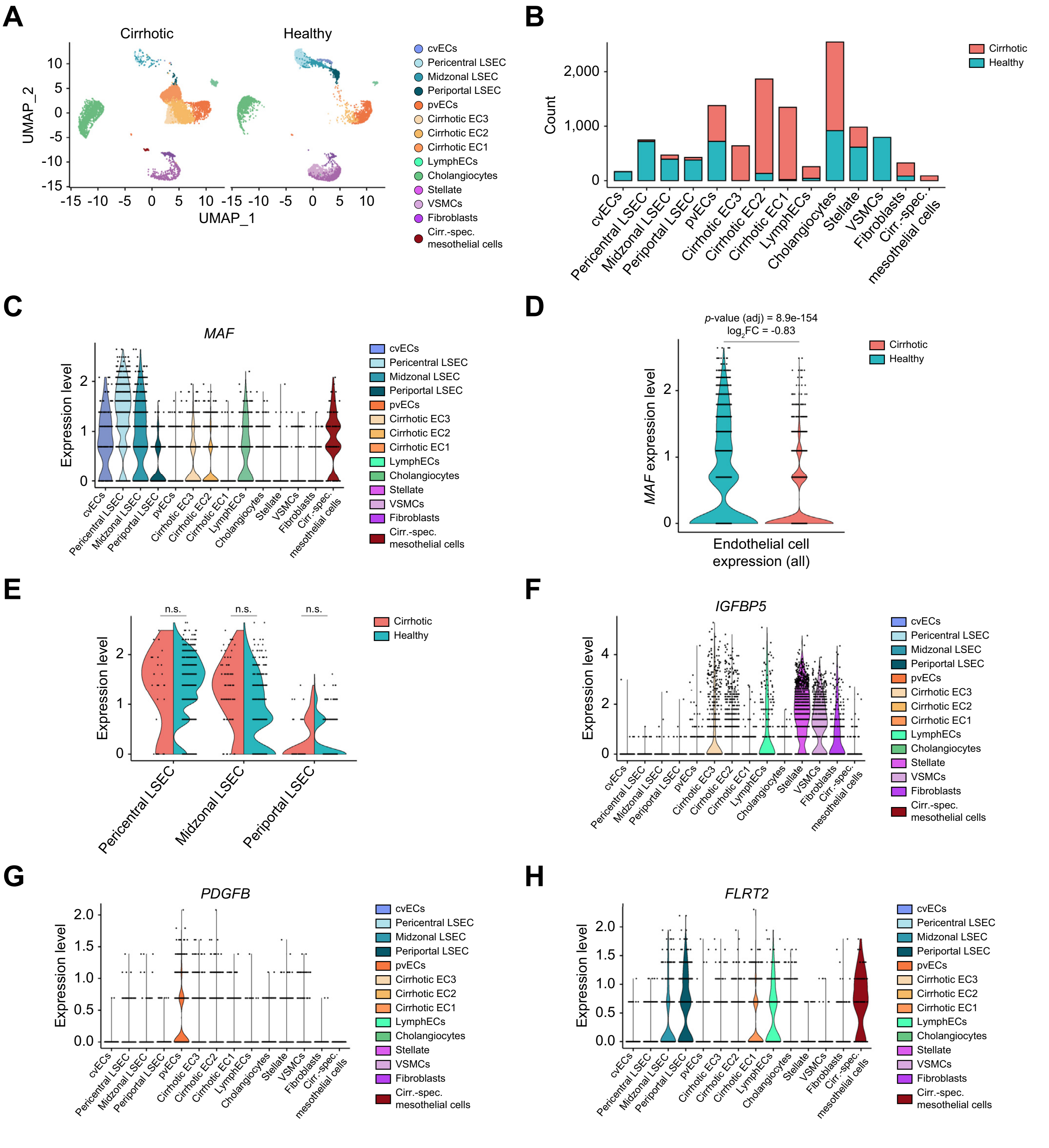

Fig. 8. Downregulation of endothelial *MAF* in cirrhotic human livers. (A) UMAP plot of endothelial cells from human cirrhotic and control livers. (B) Bar plot of cell counts mapped to the identified clusters. (C) Violin plot of *MAF* expression per cell cluster. (D) Violin plot of *MAF* expression per phenotype. (E) Violin plot for pericentral, midzonal, and periportal LSECs per phenotype. Violin plots of (F) *IGFBP5*, (G) *PDGFB*, and (H) *FLRT2* expression per cell cluster. cvECs, central vein endothelial cells; LSECs, liver sinusoidal endothelial cells; pvECs, portal vein endothelial cells; UMAP, uniform manifold approximation and projection; VSMCs, vascular smooth muscle cells.

induction of fibrosis. *Cxcl12* is a known proangiogenic chemokine[26] that is primarily expressed by hepatocytes and ECs.[27] Therefore, the role of endothelial *Cxcl12* in liver fibrogenesis requires further investigation.

Using scRNA-seq analysis, we discovered that the complete population of LSECs in $Maf^{LSEC\text{-}KO}$ mice underwent a transcriptomic switch in identity towards continuous EC differentiation characterized by homogeneously distributed *Cd34* and

Pdgfb expression. Among these dedifferentiated LSECs, four distinctive subpopulations were identified, *i.e.* capillary, proliferative, sprouting and secretory clusters. While the secretory cluster may drive the scarring reaction to liver injury via expression of profibrotic angiokines, the proliferative and sprouting clusters may promote the angiogenic part of the reactive response. In line with the emergence of the proliferative and sprouting LSEC subpopulations in $Maf^{LSEC\text{-}KO}$ mice, we identified *Myc* as the most strongly upregulated transcription factor in LSECs in $Maf^{LSEC\text{-}KO}$ mice. MYC is known to be essential for vasculogenesis and angiogenesis[28] and thus may mediate development of the proliferative and sprouting LSEC subpopulations in $Maf^{LSEC\text{-}KO}$ mice. Furthermore, our data indicate that c-Maf is an important endothelial transcription factor supporting LSEC differentiation[29] that represses *Myc,* and its vast dependent transcriptional machinery, to keep LSECs in a resting state that supports liver homeostasis.

Mechanistically, we show that the transcription factor c-Maf acts at the epigenetic level, as c-Maf deficiency resulted in remarkable changes to the chromatin accessibility landscape. Our transcription factor footprint analyses provide insight into the complex transcriptomic network of LSECs and reveal that other transcription factors, such as FOS and JUN, become more active upon *Maf* deficiency. As c-Maf forms heterodimers with FOS/JUN,[19] loss of c-Maf in LSECs might cause a higher rate of FOS-JUN heterodimerization as reflected in the TOBIAS footprinting results. Furthermore, combined genetic deletion of transcription factors *Erg* and *Fli1* in organotypic ECs also causes upregulation of FOS and JUN.[30]

Therefore, we hypothesize that upregulation of FOS/JUN may be part of a default program activated in response to loss of identity in ECs in different vascular beds. Functionally, FOS is a well-known protooncogene that can dimerize with JUN family proteins to form the AP-1 transcription factor complex.[31] FOS regulates fundamental cellular processes, including cell proliferation and differentiation, and may thus act collaboratively with MYC to support angiogenesis by FOS-dependent upregulation of proangiogenic chemokines.[32]

Our findings pave the way for the development of novel treatment strategies against liver fibrosis. Targeting the profibrotic signaling pathways of the angiocrine factors identified in this study may offer multiple therapeutic opportunities. For PDGF-BB inhibition, several small molecule tyrosine kinase inhibitors are available including imatinib, sorafenib, nilotinib, and sunitinib that suppress proliferative and fibrogenic properties of activated HSCs.[33] IGFBP5 inhibition could be achieved with the IGF-1R inhibitor linsitinib,[34] which is not currently approved for any clinical indication. In addition, erdafitinib[35] and plerixafor[36] are experimental candidates to inhibit FLRT2 and CXCL12 signaling, respectively. On the other hand, *Maf* could be targeted to increase its expression in LSECs either using lipid nanoparticles[37] or lentiviral vectors.[38]

Taken together, this study provides important insights into the role of the endothelial transcription factor c-Maf in maintaining normal liver function and protecting against hepatic fibrogenesis, and may open new avenues for developing angiotargeted strategies for liver repair.

Affiliations

[1]*Department of Dermatology, Venereology and Allergology, University Medical Center and Medical Faculty Mannheim, Heidelberg University, and Center of Excellence in Dermatology, Mannheim, Germany;* [2]*Department of Anatomy and Developmental Biology, European Center for Angioscience, Medical Faculty Mannheim, Heidelberg University, Mannheim, Germany;* [3]*Section of Genetics and Genomics, Department of Metabolism, Digestion and Reproduction, Imperial College London, London, United Kingdom;* [4]*National Heart and Lung Institute, Imperial College London, London, United Kingdom;* [5]*Laboratory of Hepato-Gastroenterology, Institut de Recherche Expérimentale et Clinique (IREC), UCLouvain; Brussels, Belgium;* [6]*Core Facility Next Generation Sequencing, Medical Faculty Mannheim, Heidelberg University, Mannheim, Germany;* [7]*Central Unit Electron Microscopy, German Cancer Research Center (DKFZ), Heidelberg, Germany;* [8]*European Center for Angioscience, Medical Faculty Mannheim, Heidelberg University, Mannheim, Germany;* [9]*Department of Dermatology and Allergy, Evangelisches Krankenhaus Düsseldorf, Düsseldorf, Germany*

Abbreviations

CDAA, choline-deficient, L-amino acid-defined; ECs, endothelial cells; HSCs, hepatic stellate cells; IF, immunofluorescence; ISH, *in situ* hybridization; KO, knockout; LSECs, liver sinusoidal endothelial cells; MASH, metabolic dysfunction-associated steatohepatitis; RNA-seq, RNA sequencing; sc, single-cell.

Financial support

This work was supported by the Deutsche Forschungsgemeinschaft, Germany (DFG, German Research Foundation): RTG/GRK 2099 (project number 259332240); CRC/SFB 1366 (project number 394046768); ICON/EB 187/8-1 (project number 413262200). IC is recipient of a Sir Henry Dale Fellowship jointly funded by the Wellcome Trust and the Royal Society (224662/Z/21/Z). DN is recipient of a PhD studentship by the British Heart Foundation (FS/4yPhD/F/20/34128). RM is the recipient of a Fonds de la Recherche Scientifique – (FNRS-F.S.R.) fellowship (1.B306.22). AR is recipient of British Heart Foundation funding (RG/17/4/32662).

Conflict of interest

The authors declare no competing interests.

Please refer to the accompanying ICMJE disclosure forms for further details.

Authors’ contributions

Conceptualization, C.D.S, P.-S.K., S.G.; Investigation, C.D.S., M.W., T.S., M.S., S.K.-Z., J.H., J.C., L.K., H.M., D.N., R.M., C.S., M.N., K.R.; Data Curation, C.D.S., M.W., J.C., L.K., H.M., R.M., C.S.; Writing – Original Draft, C.D.S., P.-S.K., S.G.; Writing – Review & Editing, C.D.S., M.W., T.S., M.S., S.K.-Z., J.H., J.C., L.K., M.N., K.R., G.D., H.M., D.N., I.C., A.R., R.M., C.S., P.-S.K., S.G.; Visualization, C.D.S., M.W., T.S., M.S., J.H., J.C., L.K., H.M., D.N., R.M, M.N., K.R.; Supervision, C.D.S., P.-S.K., S.G.; Funding Acquisition, C.D.S, M.W., P.-S.K., S.G.

Acknowledgments

We thank Stephanie Riester for excellent technical support. We thank Carmen Birchmeier (Max-Delbrück-Centrum für molekulare Medizin, Berlin-Buch) for providing the *Maf*-floxed mice. We also thank the Preclinical Models, FlowCore, and NGS Core Facility of the Medical Faculty Mannheim at Heidelberg University for their help with tissue preparation, FACS analysis, and bioinformatics. We acknowledge the SDS@hd data storage service, supported by the Ministry of Science, Research and the Arts Baden-Württemberg (MWK) and the DFG through grant INST 35/1503–1 FUGG. The authors also thank the Imperial College London High Performance Computing Service. For the publication fee we acknowledge financial support by Heidelberg University.

Supplementary data

Supplementary data to this article can be found online at https://doi.org/10.1016/j.jhepr.2025.101475.

References

Author names in bold designate shared co-first authorship

[1] **Koch P-S**, **Lee KH**, Goerdt S, et al. Angiodiversity and organotypic functions of sinusoidal endothelial cells. Angiogenesis 2021;24:289–310. https://doi.org/10.1007/s10456-021-09780-y.

[2] **Koch PS**, **Olsavszky V**, Ulbrich F, et al. Angiocrine Bmp2 signaling in murine liver controls normal iron homeostasis. Blood 2017;129:415–419. https://doi.org/10.1182/blood-2016-07-729822.

[3] Latour C, Besson-Fournier C, Gourbeyre O, et al. Deletion of BMP6 worsens the phenotype of HJV-deficient mice and attenuates hepcidin levels reached after LPS challenge. Blood 2017;130:2339–2343. https://doi.org/10.1182/blood-2017-07-795658.

[4] **Leibing T**, **Geraud C**, Augustin I, et al. Angiocrine Wnt signaling controls liver growth and metabolic maturation in mice. Hepatology 2018;68:707–722. https://doi.org/10.1002/hep.29613.

[5] **Schmid CD**, **Olsavszky V**, **Reinhart M**, et al. ALK1 controls hepatic vessel formation, angiodiversity, and angiocrine functions in hereditary hemorrhagic telangiectasia of the liver. Hepatology 2023;77:1211–1227. https://doi.org/10.1002/hep.32641.

[6] **Duan J**, **Ruan B**, **Yan X**, et al. Endothelial Notch activation reshapes the angiocrine of sinusoidal endothelia to aggravate liver fibrosis and blunt regeneration in mice. Hepatology 2018;68:677–690. https://doi.org/10.1002/hep.29834.

[7] **Wohlfeil SA**, **Häfele V**, Dietsch B, et al. Hepatic endothelial Notch activation protects against liver metastasis by regulating endothelial-tumor cell adhesion independent of angiocrine signaling. Cancer Res 2019;79:598–610. https://doi.org/10.1158/0008-5472.CAN-18-1752.

[8] Dufton NP, Peghaire CR, Osuna-Almagro L, et al. Dynamic regulation of canonical TGFbeta signalling by endothelial transcription factor ERG protects from liver fibrogenesis. Nat Commun 2017;8:895. https://doi.org/10.1038/s41467-017-01169-0.

[9] **Geraud C**, **Koch PS**, **Zierow J**, et al. GATA4-dependent organ-specific endothelial differentiation controls liver development and embryonic hematopoiesis. J Clin Invest 2017;127:1099–1114. https://doi.org/10.1172/jci90086.

[10] **Winkler M**, **Staniczek T**, **Kürschner SW**, et al. Endothelial GATA4 controls liver fibrosis and regeneration by preventing a pathogenic switch in angiocrine signaling. J Hepatol 2021;74:380–393. https://doi.org/10.1016/j.jhep.2020.08.033.

[11] **Gómez-Salinero JM**, **Izzo F**, Lin Y, et al. Specification of fetal liver endothelial progenitors to functional zonated adult sinusoids requires c-Maf induction. Cell Stem Cell 2022;29:593–609.e7. https://doi.org/10.1016/j.stem.2022.03.002.

[12] Wende H, Lechner SG, Cheret C, et al. The transcription factor c-Maf controls touch receptor development and function. Science 2012;335:1373–1376. https://doi.org/10.1126/science.1214314.

[13] **Wilkinson AL**, **Qurashi M**, Shetty S. The role of sinusoidal endothelial cells in the Axis of inflammation and cancer within the liver. Front Physiol 2020;11. https://doi.org/10.3389/fphys.2020.00990.

[14] Jutila MA, Kroese FG, Jutila KL, et al. Ly-6C is a monocyte/macrophage and endothelial cell differentiation antigen regulated by interferon-gamma. Eur J Immunol 1988;18:1819–1826. https://doi.org/10.1002/eji.1830181125.

[15] Ly6c as a new marker of mouse blood vessels: qualitative and quantitative analyses on intact and ischemic retinas n.d. https://www.mdpi.com/1422-0067/23/1/19. [Accessed 18 March 2025].

[16] **Poisson J**, **Lemoinne S**, Boulanger C, et al. Liver sinusoidal endothelial cells: physiology and role in liver diseases. J Hepatol 2017;66:212–227. https://doi.org/10.1016/j.jhep.2016.07.009.

[17] **Chen W**, **Xia P**, **Wang H**, et al. The endothelial tip-stalk cell selection and shuffling during angiogenesis. J Cell Commun Signal 2019;13:291–301. https://doi.org/10.1007/s12079-019-00511-z.

[18] Bentsen M, Goymann P, Schultheis H, et al. ATAC-seq footprinting unravels kinetics of transcription factor binding during zygotic genome activation. Nat Commun 2020;11:4267. https://doi.org/10.1038/s41467-020-18035-1.

[19] Kerppola TK, Curran T. Maf and Nrl can bind to AP-1 sites and form heterodimers with Fos and Jun. Oncogene 1994;9:675–684.

[20] Ramachandran P, Dobie R, Wilson-Kanamori JR, et al. Resolving the fibrotic niche of human liver cirrhosis at single-cell level. Nature 2019;575:512–518. https://doi.org/10.1038/s41586-019-1631-3.

[21] Czochra P, Klopcic B, Meyer E, et al. Liver fibrosis induced by hepatic overexpression of PDGF-B in transgenic mice. J Hepatol 2006;45:419–428. https://doi.org/10.1016/j.jhep.2006.04.010.

[22] Sokolović A, Sokolović M, Boers W, et al. Insulin-like growth factor binding protein 5 enhances survival of LX2 human hepatic stellate cells. Fibrogenesis Tissue Repair 2010;3:3. https://doi.org/10.1186/1755-1536-3-3.

[23] **Camillo C**, **Facchinello N**, **Villari G**, et al. LPHN2 inhibits vascular permeability by differential control of endothelial cell adhesion. J Cel Biol 2021;220:e202006033. https://doi.org/10.1083/jcb.202006033.

[24] Ando T, Tai-Nagara I, Sugiura Y, et al. Tumor-specific interendothelial adhesion mediated by FLRT2 facilitates cancer aggressiveness. J Clin Invest 2022;132:e153626. https://doi.org/10.1172/JCI153626.

[25] Hwang HJ, Kang D, Kim J-R, et al. FLRT2 prevents endothelial cell senescence and vascular aging by regulating the ITGB4/mTORC2/p53 signaling pathway, 2024. https://doi.org/10.1172/jci.insight.172678; 2024.

[26] **Chen C**, **Liu W**, **Yuan F**, et al. G protein-coupled receptor GPR182 negatively regulates sprouting angiogenesis via modulating CXCL12-CXCR4 axis signaling. Angiogenesis 2025;28:25. https://doi.org/10.1007/s10456-025-09977-5.

[27] Single-cell transcriptomics of 20 mouse organs creates a Tabula Muris. Nature 2018;562:367–372. https://doi.org/10.1038/s41586-018-0590-4.

[28] Baudino TA, McKay C, Pendeville-Samain H, et al. c-Myc is essential for vasculogenesis and angiogenesis during development and tumor progression. Genes Dev 2002;16:2530–2543. https://doi.org/10.1101/gad.1024602.

[29] Sabbagh MF, Heng JS, Luo C, et al. Transcriptional and epigenomic landscapes of CNS and non-CNS vascular endothelial cells. eLife 2018;7:e36187. https://doi.org/10.7554/eLife.36187.

[30] **Gomez-Salinero JM**, **Itkin T**, Houghton S, et al. Cooperative ETS transcription factors enforce adult endothelial cell fate and cardiovascular homeostasis. Nat Cardiovasc Res 2022;1:882–899. https://doi.org/10.1038/s44161-022-00128-3.

[31] Milde-Langosch K. The Fos family of transcription factors and their role in tumourigenesis. Eur J Cancer 2005;41:2449–2461. https://doi.org/10.1016/j.ejca.2005.08.008.

[32] **Zhao Y**, **Lei Y**, **Ning H**, et al. PGF2α facilitates pathological retinal angiogenesis by modulating endothelial FOS-driven ELR+ CXC chemokine expression. EMBO Mol Med 2023;15:e16373. https://doi.org/10.15252/emmm.202216373.

[33] Higashi T, Friedman SL, Hoshida Y. Hepatic stellate cells as key target in liver fibrosis. Adv Drug Deliv Rev 2017;121:27–42. https://doi.org/10.1016/j.addr.2017.05.007.

[34] Macaulay VM, Middleton MR, Eckhardt SG, et al. Phase I dose-escalation study of linsitinib (OSI-906) and erlotinib in patients with advanced solid tumors. Clin Cancer Res 2016;22:2897–2907. https://doi.org/10.1158/1078-0432.CCR-15-2218.

[35] Gadaleta RM, Moschetta A. Dark and bright side of targeting fibroblast growth factor receptor 4 in the liver. J Hepatol 2021;75:1440–1451. https://doi.org/10.1016/j.jhep.2021.07.029.

[36] **Qi X**, **Huang J**, **Zhang T**, et al. Unveiling CXCL12/CXCR4 Axis as a common pathway in diverse types of pulmonary fibrosis and the therapeutic potential of plerixafor. 2024. https://doi.org/10.2139/ssrn.4681859.

[37] **Liu GW**, **Guzman EB**, Menon N, et al. Lipid nanoparticles for nucleic acid delivery to endothelial cells. Pharm Res 2023;40:3–25. https://doi.org/10.1007/s11095-023-03471-7.

[38] Abel T, El Filali E, Waern J, et al. Specific gene delivery to liver sinusoidal and artery endothelial cells. Blood 2013;122:2030–2038. https://doi.org/10.1182/blood-2012-11-468579.

Keywords: Cirrhosis; Liver sinusoidal endothelial cells (LSEC); Capillarization; Single-cell RNA-Seq analysis; ATAC-Seq analysis.
Received 13 November 2024; received in revised form 28 May 2025; accepted 2 June 2025; Available online 6 June 2025

Journal of Hepatology, Volume *7*

Supplemental information

Endothelial c-Maf prevents MASLD-like liver fibrosis by regulating chromatin accessibility to suppress pathogenic microvascular cell subsets

Manuel Winkler, Theresa Staniczek, Maximilian Suhayda, Sina Wietje Kürschner-Zacharias, Johannes Hoffmann, Julio Cordero, Linda Kraske, Hannah Maude, Dorka Nagy, Rita Manco, Carsten Sticht, Michelle Neßling, Karsten Richter, Gergana Dobreva, Anna Maria Randi, Inês Cebola, Kai Schledzewski, Philipp-Sebastian Reiners-Koch, Sergij Goerdt, and Christian David Schmid

Endothelial c-Maf prevents MASLD-like liver fibrosis by regulating chromatin accessibility to suppress pathogenic microvascular cell subsets

Manuel Winkler, Theresa Staniczek, Maximilian Suhayda, Sina Wietje Kürschner-Zacharias, Johannes Hoffmann, Julio Cordero, Linda Kraske, Hannah Maude, Dorka Nagy, Rita Manco, Carsten Sticht, Michelle Neßling, Karsten Richter, Gergana Dobreva, Anna Maria Randi, Inês Cebola, Kai Schledzewski, Philipp-Sebastian Reiners-Koch, Sergij Goerdt, and Christian David Schmid

Table of contents

Supplementary Material and Methods 2
Fig. S1 15
Fig. S2 16
Fig. S3 17
Fig. S4 19
Fig. S5 20
Fig. S6 21
Fig. S7 22
Fig. S8 23
Fig. S9 24
Fig. S10 25
Table S1 26
Table S2 26
Table S3 26
Table S4 26
Supplementary References 27

Supplementary Material and Methods

Tissue preparation

Isofluran (Baxter) anesthetized mice were sacrificed by cervical dislocation. The liver was removed and weighed. Sectioned organs were fixed in phosphate-buffered 4 % formaldehyde solution (P087, Carl Roth) at room temperature for one to five days, followed by paraffin embedding according to standard protocols. Liver tissue pieces were also snap-frozen in liquid nitrogen.

Formalin-fixed paraffin-embedded (FFPE) tissue sections (3 µm) were deparaffinized and rehydrated according to standard protocols. Sections were stained with hematoxylin & eosin (H&E), Picrosirius red (PSR), and Prussian Blue according to standard protocols. For Oil Red O staining, fresh frozen cryosections were processed according to the manufacturer's standard protocols and counterstained with haematoxylin solution.

Blood collection

For blood collection, mice were deeply anesthetized and sacrificed subsequently. Blood samples were taken from the retrobulbar venous plexus during day cycle in lithium heparin tubes (Microvette 500 LH, 20.1345.100, Sarstedt). Plasma was separated using centrifugation at 7000 x g for 7 min and analyzed for standard plasma values in a Cobas c311 analyzer (Roche Diagnostics).

Immunofluorescence

3 µm paraffin sections were de-paraffinized, rehydrated, and stained according to standard protocols. Heat induced epitope retrieval (HIER) citrate buffer pH 6.0 (ZUC028-500, Zytomed Sytems) was used for antigen retrieval at 95 °C for 45 min.

After air-drying, cryosections (8 µm) were fixed for 10 min with phosphate-buffered 4 % paraformaldehyde (PFA) (0335, Carl Roth) and blocked with 5 % normal donkey serum (017-000-121, Dianova, Hamburg, Germany) for 30 min.

Antibodies were diluted in Dako antibody diluent (S202230-2, Agilent Technologies). Primary antibodies were incubated overnight at 4 °C. Subsequently, sections were washed three times with phosphate-buffered saline (PBS) (A0964.9050, VWR International) and incubated with fluorophore-conjugated secondary antibodies for one hour at room temperature followed by another washing step with PBS. Finally, sections were mounted with Dako fluorescence mounting medium (S302380-2, Agilent Technologies), dried for one day at room temperature, and stored at 4 °C. Antibodies used for immunofluorescence are listed in CTAT file.

Immunohistochemistry

Sections of 3 µm thickness were prepared from paraffin-embedded tissue, following standard protocols for deparaffinisation and rehydration. Antigen retrieval was performed at pH6 (citrate buffer) for 45 minutes. The sections were then blocked with Dako Real Peroxidase Blocking Solution (Agilent Technologies, S2023) for 10 minutes. The primary antibody was diluted in Dako Antibody Diluent (Agilent Technologies, S202230-2) and incubated overnight at 4 °C in a humid chamber. Following this, the sections were washed three times with PBS and incubated with HRP-conjugated antibodies for 1 hour at room temperature. Following a further three washes with PBS, the sections were then incubated with Dako Liquid DAB + Substrate (Agilent Technologies, K3468) for a period of 8 minutes. They were then counterstained with haematoxylin solution, Gill No. 1 (Sigma-Aldrich, GHS132-1L) for a further 4 minutes, after which they were mounted with Dako aqueous mounting medium (Agilent Technologies, S3025).

Microscopy

Images were acquired using an Eclipse Ni-E motorized upright microscope equipped with CFI Plan Apochromat Lambda series objective lenses (4x, 10x, 20x, 40x, 60x), an Intensilight Epifluorescence Illuminator, a DS-Ri2 high-definition color camera, and a DS-Qi2 high-definition monochrome camera controlled by NIS-Elements AR 5.6.30 software (Nikon Instruments). To capture the total thickness of the specimen, fluorescence microscopy images were acquired as series of z-axis images. Acquired

images were background corrected, deconvoluted, and focused using NIS-Elements AR 5.6.30 and Fiji ImageJ 2.0.0-rc69/1.53c [1,2].

Representative areas per sample were selected for quantification of immunofluorescence or in situ hybridization images. The images were quantified using ImageJ software with a pre-programmed threshold appropriate for the analysis. The area was then measured using the 'Measure' tool implemented in ImageJ.

qPCR

RNA Isolation

Liver tissue was homogenized using the Precellys® Tissue Homogenizer (Bertin Technologies, France) and the Precellys® Lysing Kit CKMix (2 mL tubes with ceramic beads) at 5000 rpm for 2 × 20 seconds. Total RNA from liver tissue and hepatic endothelial cells was isolated using innuPREP RNA Mini Kit 2.0 (845-KS-2040250, Analytik Jena) followed by DNA digestion using the TURBO DNA-free Kit (AM1907, Thermo Fisher Scientific) according to the manufacturer's protocols. RNA concentration and quality were measured using a NanoPhotometer NP80 (Implen) and a 2100 Bioanalyzer (Agilent Technologies).

Reverse Transcription

Reverse transcription was performed usingMaxima Reverse Transcriptase (EP0752, Thermo Fisher Scientific) and Oligo(dT)18 primers (SO131, Thermo Fisher Scientific) according to the manufacturer’s instructions.

qPCR settings and analysis

innuMIX qPCR SyGreen Sensitive (845-AS-1310200, Analytik Jena, Jena, Germany) was used on a qTOWER 3 G touch thermal cycler (Analytik Jena) for quantitative PCR (qPCR). qPCR primers were designed with NCBIs PrimerBLAST (https://www.ncbi.nlm.nih.gov/tools/primer-blast/). For mRNA specificity, qPCR primers were designed to span an exon-exon junction where possible. Primers were tested with no template controls, original RNA, and melt curve analysis. Primer sequences are listed in

the CTAT file. qPCR output files were analyzed in qPCRsoft 4.0.8.0 (Analytik Jena). Considering the amplification efficiencies determined from the standard curves, normalized expression values were calculated using the Pfaffl method. For normalization, reference genes *Gak*, *Mrpl46*, and *Srp72* [3] were used.

Tissue Collagen Assay

Liver tissue was analyzed for collagen content using the Sensitive Tissue Collagen Assay (QZBtiscol1, QuickZyme) according to the manufacturer's instructions. Optical densities were detected with an Infinite 200 plate reader (Tecan Group) set to 570 nm.

Hepatic triglyceride concentration assay

To analyze the hepatic triglyceride concentration, 100 mg snap frozen liver tissue was homogenized in 5 % NP-40 solution (74385, Merck) by two cycles of heating to 80–100°C for 5 minutes using a shaking dry incubator (ThermoMixer C, Eppendorf, Hamburg, Germany) and cooling to room temperature. The supernatant was used to determine the triglyceride concentration after centrifugation at top speed for two minutes. Triglyceride concentration was analyzed using Triglyceride Assay Kit – Quantification (ab65336, Abcam) based on the manufacturer's protocol.

Transmission electron microscopy (TEM)

Dissected liver lobes of six months old mice were immersed in freshly prepared aldehyde fixative (4% formaldehyde, 2 % glutaraldehyde, 1 mM MgCl2 and 1 mM CaCl2 buffered to pH 7.2 in 100 mM Na-cacodylate). Vibratome sections (200 µm) of liver lobes were postfixed with1 % osmium tetroxide, dehydrated with ethanol and embedded in epoxy resin. The ultra-thin sections (60 nm) contrast-stained with uranyl and lead were viewed in a Zeiss EM 910 at 80 kV (Carl Zeiss, Oberkochen, Germany). Micrographs were taken with a digital CCD-Camera (TRS, Moorenweiss, Germany).

In-situ-hybridization

For RNA in situ hybridization (ISH), RNAscope 2.5 HD Red (322350, Advanced Cell Diagnostics) on FFPE tissue sections (3 µm) according to the manufacturer's protocols was used. ISH probes are listed in CTAT file.

RNA fluorescence in situ hybridization (FISH)

FFPE tissue sections (3 µm) were used for RNA fluorescence in situ hybridization (FISH) and processed according to the manufacturer's protocols. The RNAscope 2.5 HD Duplex kit (Advanced Cell Diagnostics, 322,430) was used. FISH probes are listed in CTAT file.

MASH-induced liver fibrosis mouse model

To induce MASH-induced liver fibrosis 11-week-old female *Maf*$^{LSEC\text{-}KO}$ and littermate controls were fed choline-deficient, L-amino acid-defined (CDAA) diet (E15666-94, Ssniff) for ten weeks. The body weight was monitored during the experiment and blood samples were taken at the end of the experiment prior scarification of the mice.

Isolation of hepatic endothelial cells

Isolation of hepatic endothelial cells was performed as described before [4]. Mice were deeply anesthetized with Isolfluran (Baxter) and sacrificed by cervical dislocation. Livers were perfused *in situ* via the portal vein with a 0.05 % collagenase/amino acid/saccharide solution (C2674, Sigma-Aldrich). Livers were dissected and mechanically disrupted. Liver tissue from three mice was pooled and digested at 37 °C in collagenase/Gey's balanced salt solution (G9779, Sigma-Aldrich). After removing hepatocytes by low speed centrifugation, the cells were filtered through a 250 µm mesh followed by a 100 µm cell strainer. Non-parenchymal cells were separated by a 19.3 % Nycodenz (1002424, Axis-Shield, Alere Technologies) gradient. Subsequently, magnetic-activated cell sorting (MACS) was used with anti-CD146 MicroBeads (ME-9F1, 130-092-007, Miltenyi Biotech) to purify endothelial cells. The purity of hepatic endothelial cells was analyzed by fluorescence activated cell

sorting (FACS) using a BD FACSCanto II (BD Biosciences, Franklin Lakes, NJ, USA). After gating on live singlet cells, the purity of hepatic endothelial cells was indicated by LYVE1+ and/or CD31+ cells. Positivity for LYVE-1 and/or CD31 of hepatic endothelial cells from controls was 95 %, for *Maf*$^{\text{LSEC-KO}}$ was 86 % (Fig. S6A). Macrophage contamination was assessed by CD11b positivity. Positivity of hepatic endothelial cells for CD11b was 2 % for controls and 4 % for *Maf*$^{LSEC\text{-}KO}$ (Fig. S5A).

Bulk RNA-seq of mouse hepatic endothelial cells

Library preparation and sequencing

RNA of isolated hepatic endothelial cells was conducted as described above. Library preparation and sequencing was conducted by BGI Tech Solutions (Hong Kong) using the BGISEQ-500 platform and paired-end 100-bp read length. Raw reads were filtered using the SOAPnuke software to obtain clean reads (filter parameters: -n 0.03 -l 20 -q 0.4 -A 0.28). At least 20×10^6 clean reads per sample were obtained.

Data analysis

RNA-seq data processing was performed with R (version 3.6.3) and bioconductor (version 3.9) using the NGS analysis package systempipeR [5] in Rstudio (version 1.1.463). Quality control of clean sequencing reads was performed using FastQC (Babraham Bioinformatics). Low-quality reads were removed using trim_galore (version 0.6.4). The resulting reads were aligned to the mouse genome version GRCm38.p6 from GeneCode and counted using kallisto version 0.46.1 [6]. The count data was transformed to log2-counts per million (logCPM) using the voom-function from the limma package [7]. Differential expression analysis was performed using the limma package in R. A false positive rate of $\alpha= 0.05$ with FDR correction was taken as the level of significance.

Volcano plots and heatmaps were created using ggplot2 package (version 2.2.1) and the complexHeatmap package (version 2.0.0) [8]. For Gene Ontology enrichment analysis and gene set enrichment analysis (GSEA), clusterProfiler was used [9]. Only genes with RNA expression >1cpm

were used in the analysis. For Gene Ontology enrichment analysis, genes were further filtered for log2 fold-change >1 or <-1, respectively. GSEA was performed using key genes of LSEC zonation, based on data from Su et al. [10].

ATAC-seq of mouse hepatic endothelial cells

Library preparation

Hepatic endothelial cells were isolated from three 12 weeks old control and *Maf*$^{LSEC\text{-}KO}$ mice using a modified protocol from [11]. Briefly, 50000 freshly-isolated hepatic endothelial cells were incubated in 50 µL cold lysis buffer (10 mM Tris-HCl pH 7.4, 10 mM NaCl, 3 mM MgCl2, 0.1 % Igepal CA-630) for three minutes. The lysis was stopped by adding 1 mL resuspension buffer (10 mM Tris-HCl pH 7.4, 10 mM NaCl, 3 mM MgCl2) and the nuclei were pelleted by 10 min centrifugation (500 x g, 4 °C). Isolated nuclei were incubated in transposition reaction mix containing 2.5 µL Nextera Tagment DNA Enzyme TDE (15027916, Illumina) in 47.5 µL 2x transposition buffer (20 mM Tris-HCl pH 7.6, 10 mM MgCl2, 20% Dimethylformamide) for 30 min at 37 °C. Immediately following the transposition reaction, purification was carried out using ChIP DNA Clean and Concentrator Kit (D5205, Zymo). Sequencing libraries were performed using NEB Next High-Fidelity 2x PCR Master Mix (M0541S, New England Biolabs) and Nextera Index Kit (15055290, Illumina). Magnetic bead purification with two-sided size selection using undiluted Agencourt AMPure XP Beads (A63881, Beckman Coulter) ensured library sizes between 150 and 1000 bp. The quality of the libraries was analyzed by Bioanalyzer High Sensitivity DNA analysis kit (5067-4626, Agilent).

Sequencing

Libraries were mixed in equimolar ratios and sequenced on NextSeq550 platform using v2.5 chemistry (15058251, 15057931, 15057941, 20022408, Illumina Nextseq 500/550).

Data analysis

All raw reads were trimmed using Trimmomatic-0.36 with the parameters (ILLUMINACLIP:2:30:10 LEADING:3 TRAILING:3 SLIDINGWINDOW:4:15 MINLEN:20 CROP:70 HEADCROP:10. The

trimmed reads were mapped to the mouse genome from UCSC version mm10 using Bowtie2 (v2.4.4) (default settings). Mapped reads were converted to from sam to bam by the help of samtools (v1.13). PCR duplicates were removed from the bam files with the help of MarkDuplicates.jar from Picard1.119. Bam files were merged by the help of bamtools (v2.5.1) and from the merged files the bigwig files were created. We used bamCoverage from deptools (v3.5.4) (-bs 20 –smoothLength 40 -p max –normalizeUsing RPKM -e 150). Peak calling was performed using MACS2 (v2.2.9.1). Detected peaks were annotated with annotatePeaks.pl from homer (v4.11) [12]. The peaks overlapping the blacklist from mm10 genome were removed. Peaks from controls and *Maf*$^{LSEC\text{-}KO}$ were merged using bedtools (v2.30.0) merge (-d 100). Quantification of bam files on merged peaks was performed using bedtools (v2.30.0) multicov (defaults settings). Differential binding was performed using DESEQ2 (v1.42.1).

Transcription factor footprint analysis

Transcription factor footprint analysis was performed using TOBIAS (v0.16.1) following the program instructions. The JASPAR 2024 vertebrate database was used as a motif reference. The detected differential footprints were annotated to the mouse genome (mm10) using the ChIPseeker package [13].

ATAC-Seq Data Visualization

The interactive genome browser (IGV) program was used to visualize the normalized bigwig files from the ATAC-seq data, as well as the peak files.

ATAC-seq Code Availability

All the codes used in these manuscripts are available upon request to the corresponding authors.

Single-cell RNA-seq analysis of mouse LSEC

Suspension Preparation

Hepatic endothelial cells from three *Maf*$^{LSEC\text{-}KO}$ and three control mice were isolated and pooled as described above.

Hepatic endothelial cells suspensions were adjusted to a concentration of approximately 1,000 cells/µL in PBS to ensure optimal capture efficiency for 10x Genomics Chromium Next GEM technology.

10x Chromium Platform

Single-cell suspensions were loaded into the 10x Genomics Chromium Controller (10x Genomics) to generate Gel Bead-in-Emulsions (GEMs), following the manufacturer's instructions. A targeted cell recovery of 10,000 cells was used to ensure sufficient capture while minimizing doublet formation.

Inside each GEM, reverse transcription was performed using reagents from the 10x Genomics Chromium Single Cell 3' Reagent Kit v3.1. Barcoded mRNA transcripts were converted into cDNA within the emulsion droplets. Following reverse transcription, cDNA was purified with Silane Dynabeads (Thermo Fisher Scientific), and the cDNA libraries were amplified by polymerase chain reaction (PCR) to generate sufficient material for sequencing.

Amplified cDNA was subjected to fragmentation, end repair, and A-tailing using the Chromium Single Cell 3' Library Construction Kit. Adapter ligation and final amplification were performed to generate sequencing-ready libraries. Sample quality was assessed using an Agilent Bioanalyzer (Agilent Technologies) to ensure library integrity and appropriate fragment sizes.

Sequencing

Final libraries were paired-end sequenced on an Illumina NovaSeq 6000 platform (Illumina) at a targeted depth of 200,000 reads per cell.

Data processing

The raw sequencing data were processed using the 10x Genomics Cell Ranger pipeline (v6.0), which performed demultiplexing, barcode assignment, and alignment to the mouse reference genome (mm10).

On the raw matrix output of Cell Ranger, the empty droplets were identified using low UMI counts (500 UMIs), and Otsu's Method, as described in Ben-Moshe et al. [14]. The mean UMI counts of the empty droplets were then subtracted from each cell. Further analyses were performed using the Seurat

package (version 5.0.1) [15]. Cells with total UMI counts lower than 1500 or total gene counts lower than 750 were removed. Cell clustering was based on PCA dimensionality reduction using 16 or 20 PCs (for the control and *Maf*$^{LSEC\text{-}KO}$ data, respectively) and a resolution value of 0.3. For further analysis, mitochondrial ("^mt-") and ribosomal ("Rp") genes were manually removed from the gene list since they are prone to batch-related expression variability. Additionally, doublets were identified and removed using the DoubleFinder Package (version 2.0.3) [16] . Cell type-specific markers were then utilized to interpret the single-cell clusters: *Adgre1* and *Clec4f* for macrophages, *Ptprc* and *Cd52* for immune cells, *Acta1* for hepatic stellate cells, *Arg1* for hepatocytes, and *Pecam1* for endothelial cells. The clustering was used to subset only the endothelial cells. The last subset was done excluding the cells that expressed *Vwf* to delete the vascular endothelial cells [17]. This processing was done independently for each sequencing run. Finally, the processed control and *Maf*$^{LSEC\text{-}KO}$ Seurat Objects were merged. Data were normalized and scaled using the SCTransform function. Cell clustering was based on PCA dimensionality reduction using 22 PCs and a resolution value of 0.3. The resulting clusters were then manually annotated after extracting cluster markers using the FindAllMarkers Seurat function.

Zonation reconstruction of LSEC

The LSEC zonation was computed by adapting the Method used in Ben-Moshe et al. [14]. Specifically, as starting landmarks well-established pericentral landmark genes were used [18]: *Cdh13*, *Wnt2*, *Rspo3*, and *Wnt9b* for the control cells, while for *Maf*$^{LSEC\text{-}KO}$ only *Cdh13* and *Wnt2* were used, as *Wnt9b* and *Rspo3* were too low expressed. After computing Spearman correlations for each gene with the combined expression level of these pericentral landmark genes, genes with mean expression higher than 1E-5 for the control and 1E-6 for the *Maf*$^{LSEC\text{-}KO}$, correlation pval lower than 0.01 and correlation higher than 0.3 and lower than -0.2 (control) and -0.1 (*Maf*$^{LSEC\text{-}KO}$) were considered as central or portal landmark genes, respectively. Following a two-step normalization process which involved normalizing based on the sum of the total UMI count for each cell and scaling by dividing them by their maximal expression, we calculated the zonation coordinate as the ratio of pericentral landmark gene expression

to the sum of central and pericentral landmark gene expression (pLM/(cLM+pLM)). Finally, cells were equally assigned to four discrete zones – periportal, mid-lobule 1 and 2, and pericentral – based on percentiles. To derive zonation profiles of genes, we computed the mean expression levels and corresponding standard errors for cells grouped by zone.

In vitro stimulation of LX-2 cells

For stimulation of human hepatic stellate cell line LX-2 (RRID:CVCL_5792, SCC064, Sigma-Aldrich) with recombinant proteins, LX-2 cells were seeded at a density of 50.000 cells per well in a 6-well plate. After starving with 0.5 % FCS for 24 h, the cells were stimulated with 100 ng/mL CXCL12 (Bio-Techne), 800 ng/mL FLRT2 (Bio-Techne), 100 ng/mL IGFBP-5 (Bio-Techne) or 20 ng/mL PDGF-BB (Bio-Techne), respectively. PBS was used as control for CXCL12, FLRT2 and IGFBP-5, 4 mM HCl was used as control for PDGF-BB. After 24 h stimulation, the cells were harvested and RNA isolated using innuPREP RNA Mini Kit 2.0 (845-KS-2040250, Analytik Jena) followed by DNA digestion using the TURBO DNA-free Kit (AM1907, Thermo Fisher Scientific) according to the manufacturer's protocols. RNA concentration was measured using a NanoPhotometer NP80 (Implen). The cell line was regularly tested for mycoplasma absence by PCR. The PCR Mycoplasma Detection Kit (Biozol, ABM-G238) was used according to the manufacturer's instructions.

Reverse transcription was performed using Maxima Reverse Transcriptase (EP0752, Thermo Fisher Scientific) and Oligo(dT)18 primers (SO131, Thermo Fisher Scientific) according to the manufacturer’s instructions.

qPCR was performed using innuMIX qPCR SyGreen Sensitive (845-AS-1310200, Analytik Jena, Jena, Germany) on a qTOWER 3 G touch thermal cycler (Analytik Jena). qPCR primers were designed with NCBIs PrimerBLAST (https://www.ncbi.nlm.nih.gov/tools/primer-blast/). For mRNA specificity, qPCR primers were designed to span an exon-exon junction where possible. Primers were tested with no template controls, original RNA, and melt curve analysis. Primer sequences are listed in CTAT file. qPCR output files were analyzed in qPCRsoft 4.0.8.0 (Analytik Jena). Considering the amplification

efficiencies determined from the standard curves, normalized expression values were calculated using the Pfaffl method. For normalization the reference genes *CACTIN* and *HLCS* were used.

Analysis of MAF expression in human liver single-cell RNA-seq data

MAF expression was explored in published single-cell RNA-seq (scRNA-seq) data from human livers with a healthy or cirrhotic phenotype [Ramachandran et al. 2019, https://www.nature.com/articles/s41586-019-1631-3] [19]. Cell count matrices, barcodes and gene labels were downloaded from GEO (GEO accession: GSE136103) for all samples and read into R using the Seurat package (v4.3.0) [https://satijalab.org/seurat/authors - use Hao and Hao et al. Integrated analysis of multimodal single-cell data. Cell (2021) for Seurat v4]. Sample-level processing was carried out using the Seurat SCTransform v2 [20] pipeline along with scDblFinder [21] to remove doublets. Clusters were identified using Seurat FindNeighbors and FindClusters, and low-quality cells were filtered out by removing clusters with a mitochondrial gene in the top five marker genes. Processed samples were integrated using SelectIntegrationFeatures > PrepSCTIntegration > FindIntegrationAnchors > IntegrateData > RunPCA > RunUMAP > FindNeighbors > FindClusters. Cell type labels were predicted via integration with the Liver Cell Atlas [22]. Briefly, cell annotation and count matrices were downloaded from the Liver Cell Atlas (www.livercellatlas.org) and processed with SCTransform v2, RunPCA and RunUMAP. The Seurat commands FindTransferAnchors > TransferData were used to predict cell type labels for the Ramachandran et al. data.

Further analysis was concentrated on NPCs by isolating clusters labelled as stromal cells, fibroblasts, cholangiocytes and endothelial cells. Cells were subset for those with nFeature_RNA > 200 and percent.mt < 15 and the above steps repeated to remove small clusters of contaminating non-NPCs. Using the high-confidence set of NPCs, cell types were first assigned independent for healthy and cirrhotic samples (stage 1) and subsequently for all healthy and cirrhotic NPCs processed together (stage 2), with cell types assigned in stage 1 used to inform the final identity of combined clusters. Cell types were predicted using the label transfer method as above but with the CD45- Liver Cell Atlas,

combined with marker gene expression, namely: *PROX1*, *FLT4*, *PDPN,* and *LYVE1* for lymphatic endothelial cells, LSEC marker genes as published by Nagy et al. [23], stellate cell marker genes from PangloaDB 2020, *VWF*, *CD34*, *ENG*, *ACKR1,* and *PECAM1* for macrovascular ECs, *FCGR2B*, *STAB2*, *LYVE1*, *CD14*, *ICAM1*, *FCN3,* and *FCN2* for pericentral LSECs and *PECAM1*, *F8*, *SPARCL1,* and *CLEC14A* for periportal LSECs. Healthy NPC clusters were labelled as pericentral, midzonal and periportal LSEC, lymphatic EC, central vein EC (cvEC) and portal vein EC (pvEC). Notably, we did not observe clear expression of pericentral LSEC marker genes in the cirrhotic NPCs. Three EC clusters identified in the combined data analysis (stage 2) contained cells almost entirely from cirrhotic samples and were labelled as cirrhosis-specific ECs. Combined cell clusters from stage 2 were used to interrogate differential gene expression using the Seurat command, FindMarkers.

Fig. S1

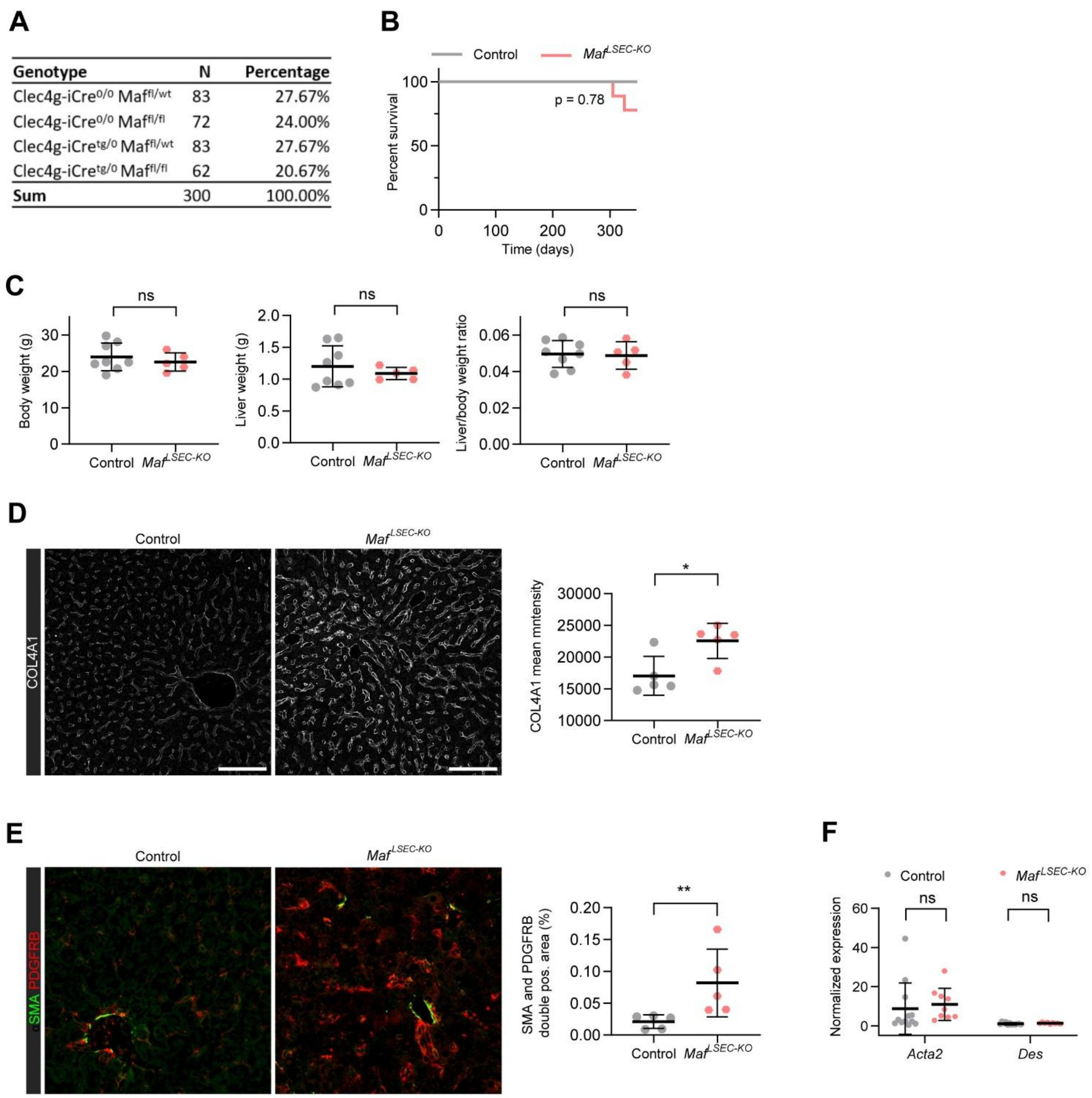

Genotype	N	Percentage
Clec4g-iCre$^{0/0}$ Maf$^{fl/wt}$	83	27.67%
Clec4g-iCre$^{0/0}$ Maf$^{fl/fl}$	72	24.00%
Clec4g-iCre$^{tg/0}$ Maf$^{fl/wt}$	83	27.67%
Clec4g-iCre$^{tg/0}$ Maf$^{fl/fl}$	62	20.67%
**Sum**	300	100.00%

Fig. S1. Basic animal data for *Maf$^{LSEC-KO}$* mice aged three months.

(A) Genotype distribution of *Maf$^{LSEC-KO}$* mice (*Clec4g-icre$^{tg/0}$ x Maf$^{fl/fl}$*) (n = 300). (B) Kaplan-Meier-curve over a period of 345 days (n = 8, 9). (C) Body and liver weight and liver/body weight ratio (n = 5, 8). (D) Immunofluorescence staining and intensity quantification of COL4A1 in the liver (n = 5). (E) Immunofluorescence staining and quantification of SMA and PDGFRB double positive area in the liver (n = 5). (F) Expression levels of *Acta2* and *Des* in livers (n = 9, 12). Scale bars: 100 µm. Mean ± SD. (B) Log-rank test; (C-F) Welch's t test; n.s. $p \geq .05$; * $p < .05$; ** $p < .01$.

Fig. S2

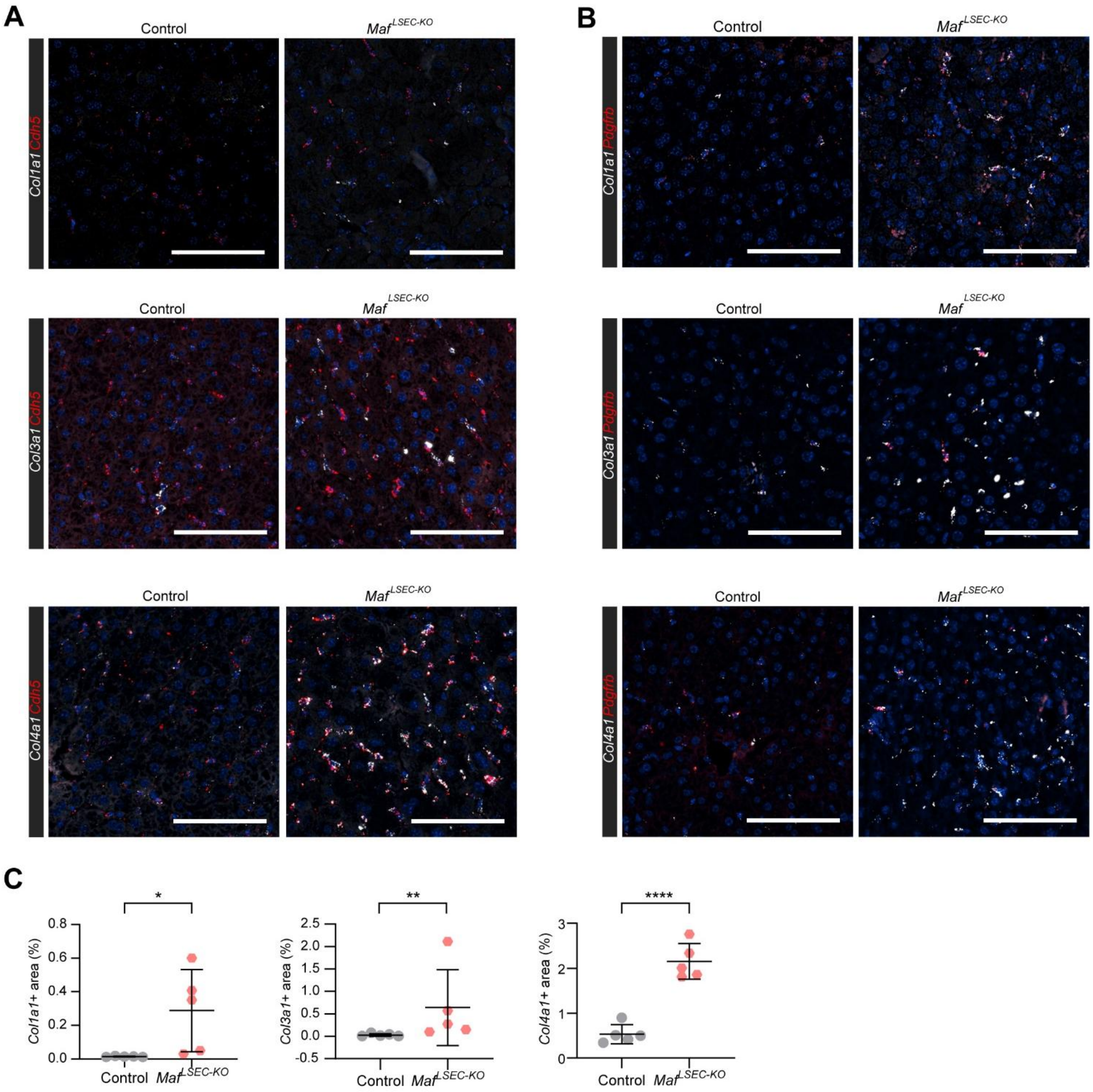

Fig. S2. FISH of collagen genes in *Maf*$^{LSEC\text{-}KO}$ mice.

(A) FISH of liver tissue for *Col1a1*, *Col3a1,* and *Col4a1* using *Cdh5* as an endothelial marker ($n = 5$). (B) FISH of liver tissue for *Col1a1*, *Col3a1,* and *Col4a1* using *Pdgfrb* as a marker for activated HSC ($n = 5$). (C) Quantification of *Col1a1*, *Col3a1,* and *Col4a1* areas in (A) ($n = 5$). (C) Welch's *t* test; * $p < .05$; ** $p < .01$, **** $p < .0001$.

Fig. S3

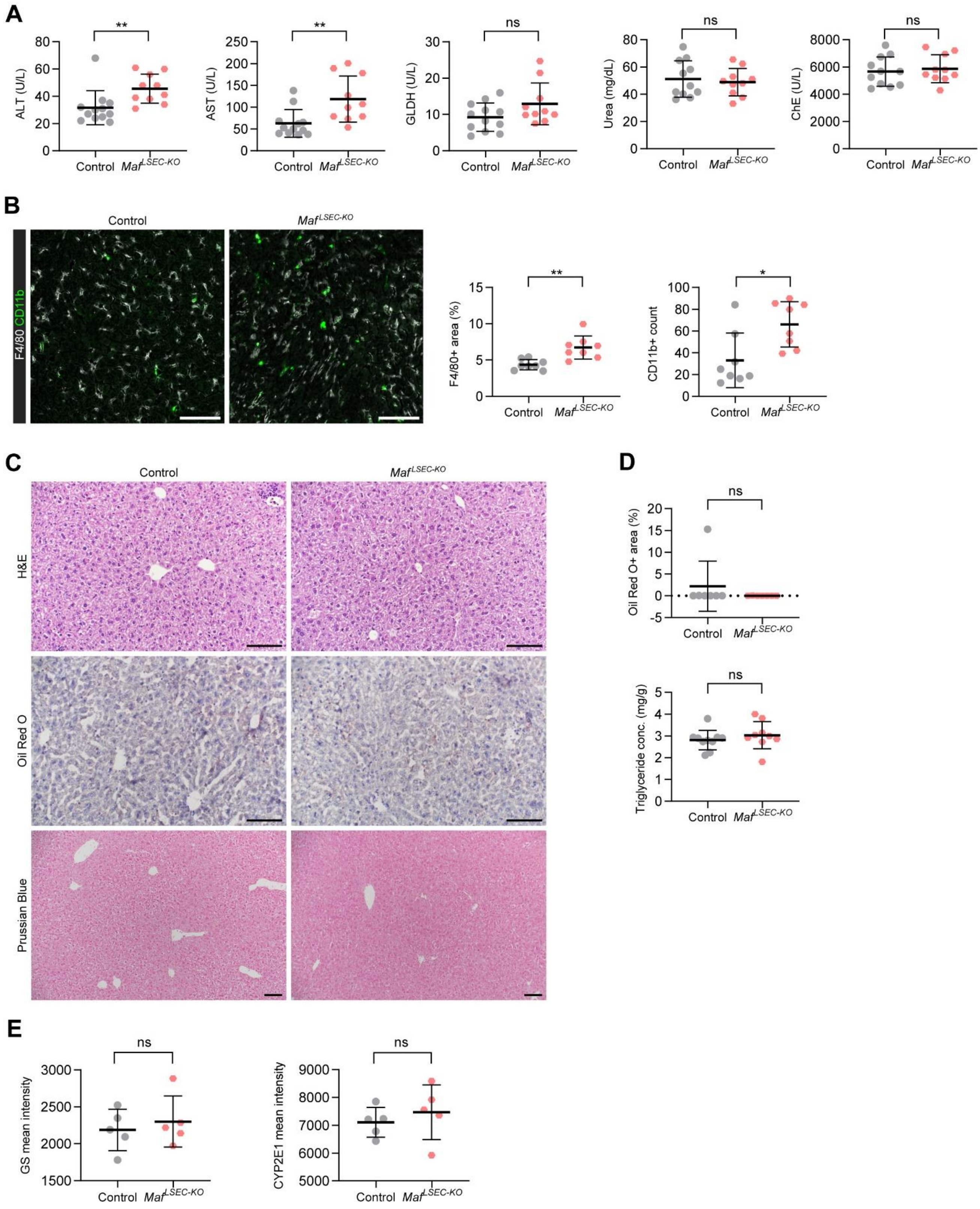

Fig. S3. Plasma values and histology for *Maf*$^{LSEC-KO}$ mice.

(A) Blood plasma levels of ALT, AST, GLDH, Urea and Cholinesterase (ChE) (n = 10, 11, 12). (B) Immunofluorescence staining and quantification of F4/80 and CD11b (n = 8). (C) H&E, Oil Red O, and Prussian blue

staining (*n* = 7, 10). (D) Quantification for Oil Red O staining (n = 7) and triglyceride concentration of livers (n = 9, 10). (E) Quantification of GS and CYP2E1 mean intensity (n = 5). Scale bars: 100 µm. Mean ± SD. (A) Mann-Whitney *U* test; (B, D, E) Welch's *t* test; n.s. $p \geq .05$; * $p < .05$; ** $p < .01$.

Fig. S4

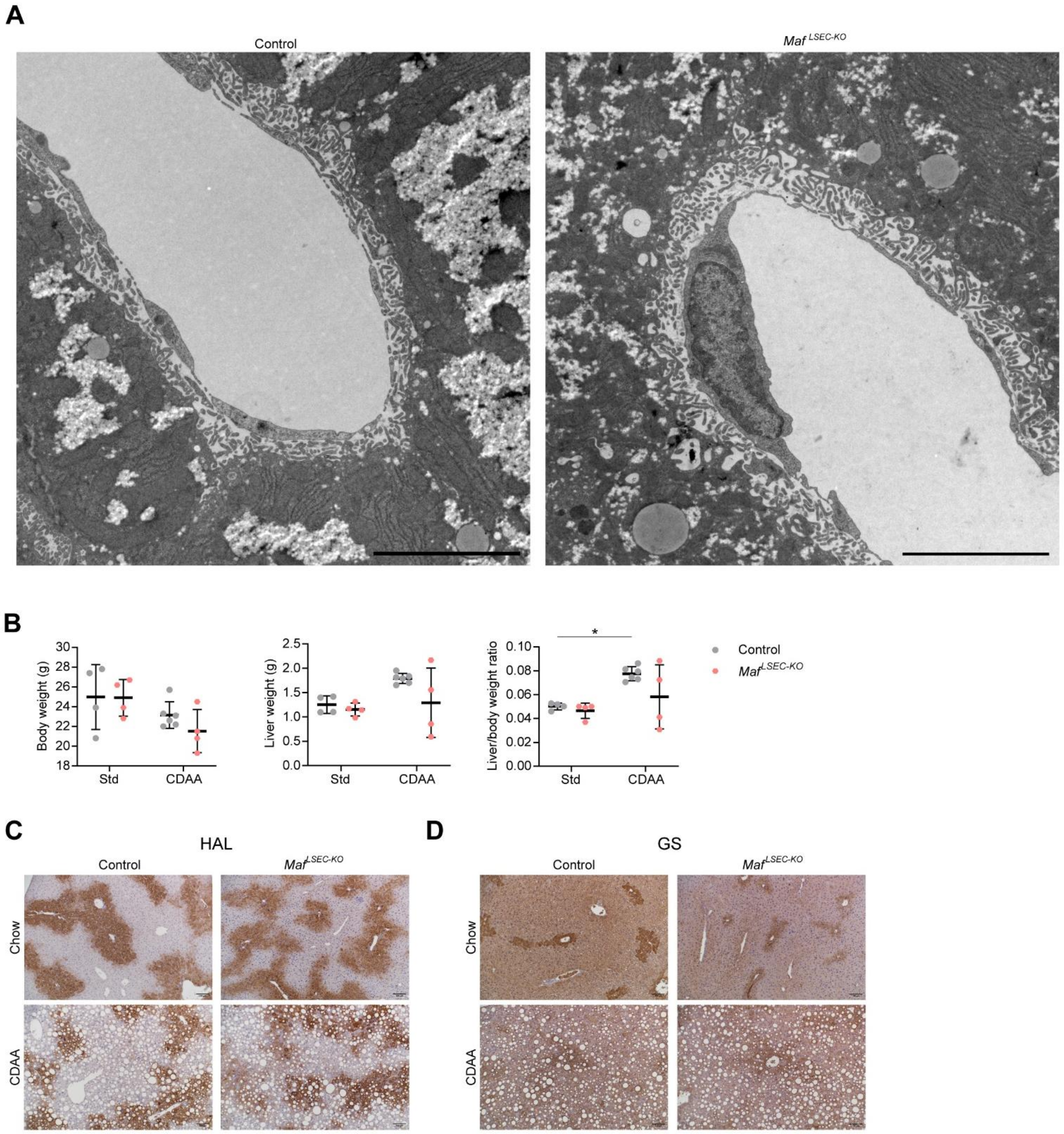

Fig. S4. Transmission Electron Microscopy of Chow fed mice; and basic animal data and metabolic zonation of CDAA diet fed mice.

(A) Transmission Electron Microscopy overview of liver sinus. (B) Body weight, liver weight, and liver/body weight ratio after Chow and CDAA diet (n = 4, 6). IHC staining of (C) HAL and (D) GS in livers after Chow and CDAA diet (n = 4, 6). (A) Scale bars: 5 µm. (C-D) Scale bars: 100 µm. (B) Two-way ANOVA and Tukey’s post-hoc test; * $p < .05$.

Fig. S5

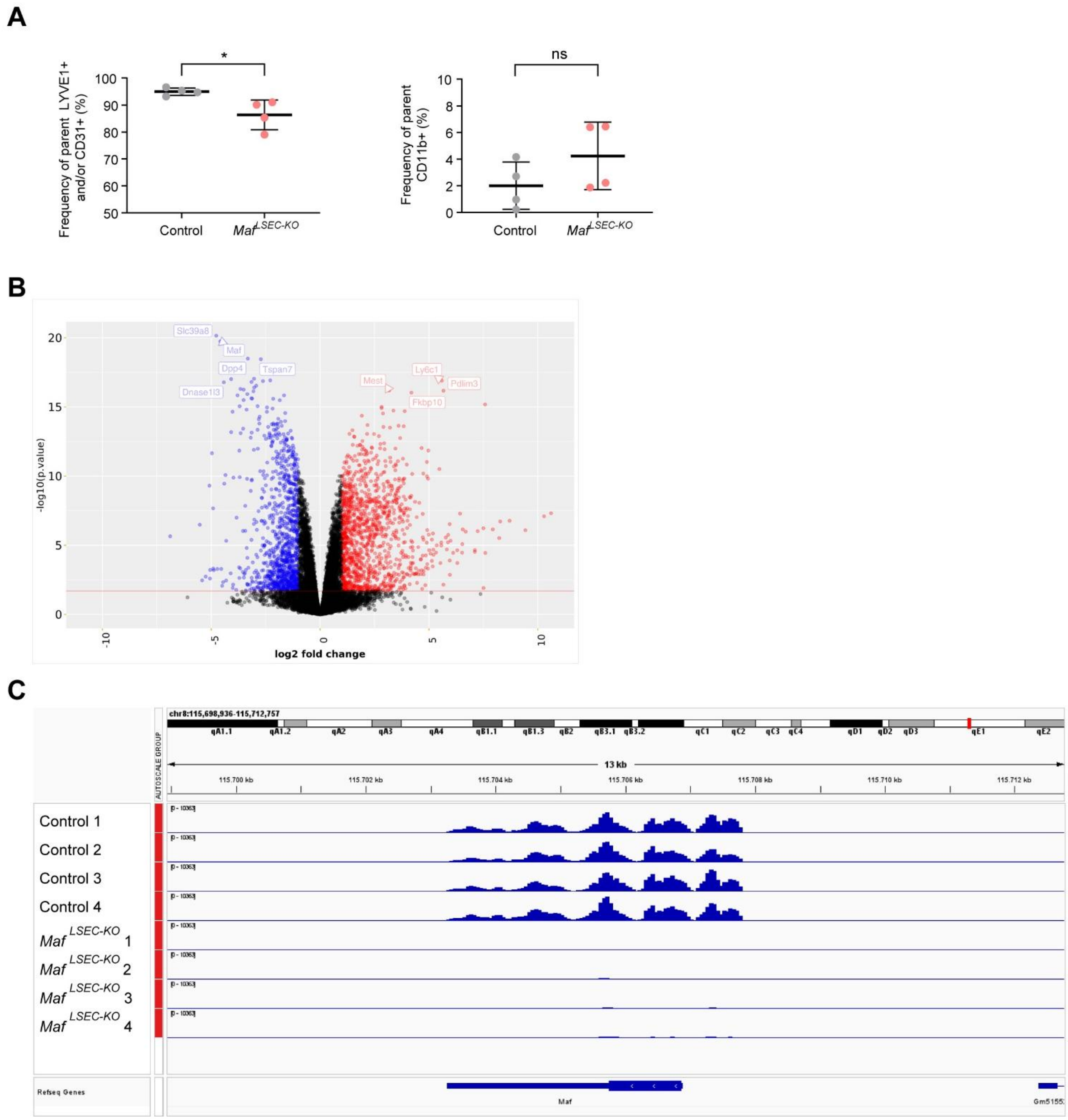

Fig. S5. Bulk RNA-seq analysis of LSEC.

(A) FACS analysis of isolated LSEC using sinusoidal endothelial marker LYVE1, endothelial marker CD31 and myeloid marker CD11b. (B) Volcano plot of fold changes and p values of bulk RNA-seq data of LSEC. (C) Gene track for bulk RNA-seq signal from isolated LSEC at the *Maf* locus for controls and $Maf^{LSEC\text{-}KO}$ mice (n = 4). Scale bars: 100 µm. Mean ± SD. (A) Welch's t test; n.s. $p \geq .05$; * $p < .05$.

Fig. S6

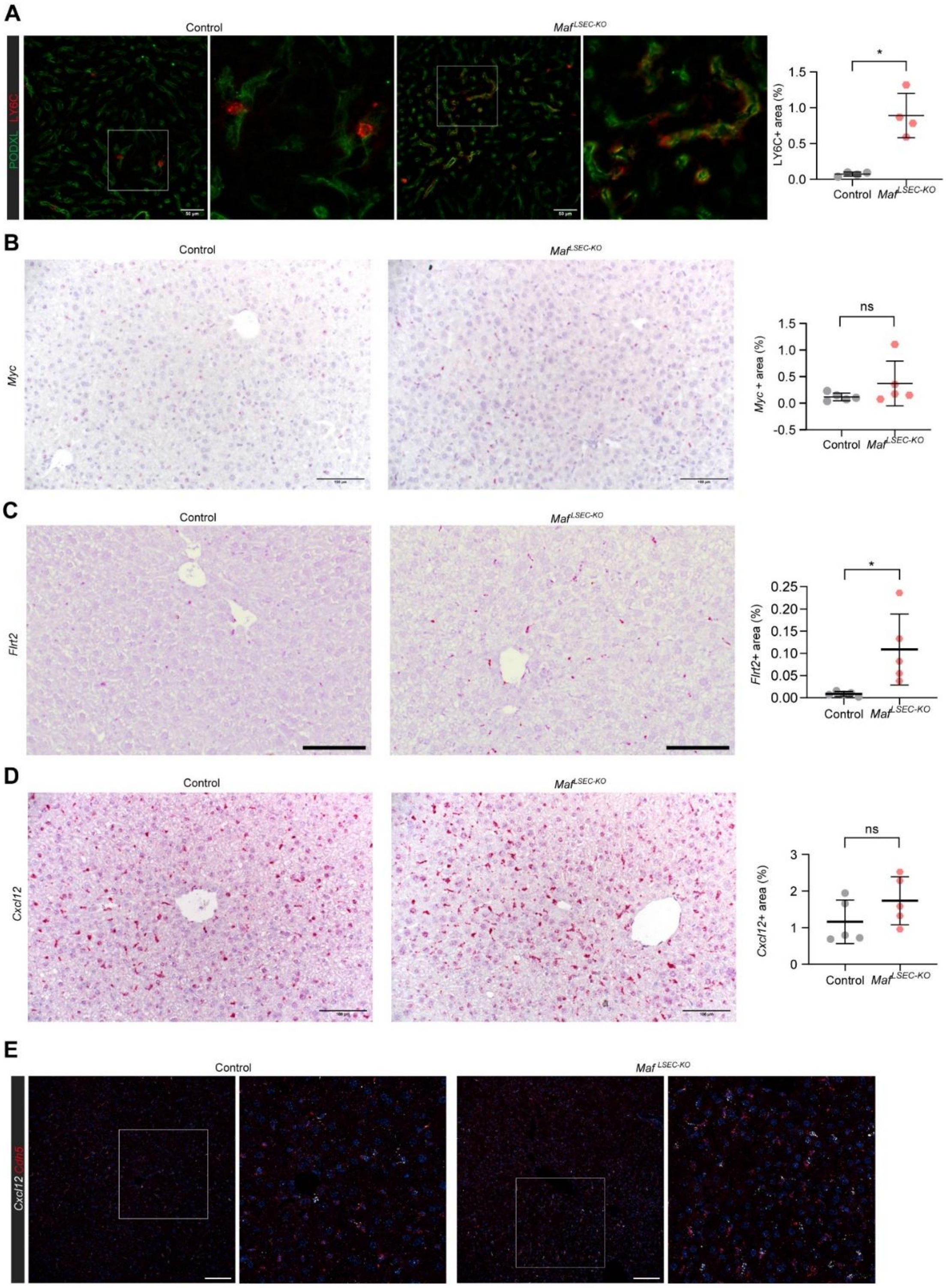

Fig. S6. Immunofluorescence staining and *in situ* hybridization of dysregulated genes identified by bulk RNA-seq.

(A) Immunofluorescence staining with zoom-in for PODXL and LY6C and quantification of LY6C area (n = 5). (B) *In situ* hybridization and quantification of (B) *Myc,* (C) *Flrt2,* and (D) *Cxcl12* (n = 5). (E) FISH of *Cxcl12* and *Cdh5* with zoom-in (n = 5). (B) Mann-Whitney *U* test; (A, C-E) Welch's *t* test. n.s. $p \geq .05$; * $p < .05$.

Fig. S7

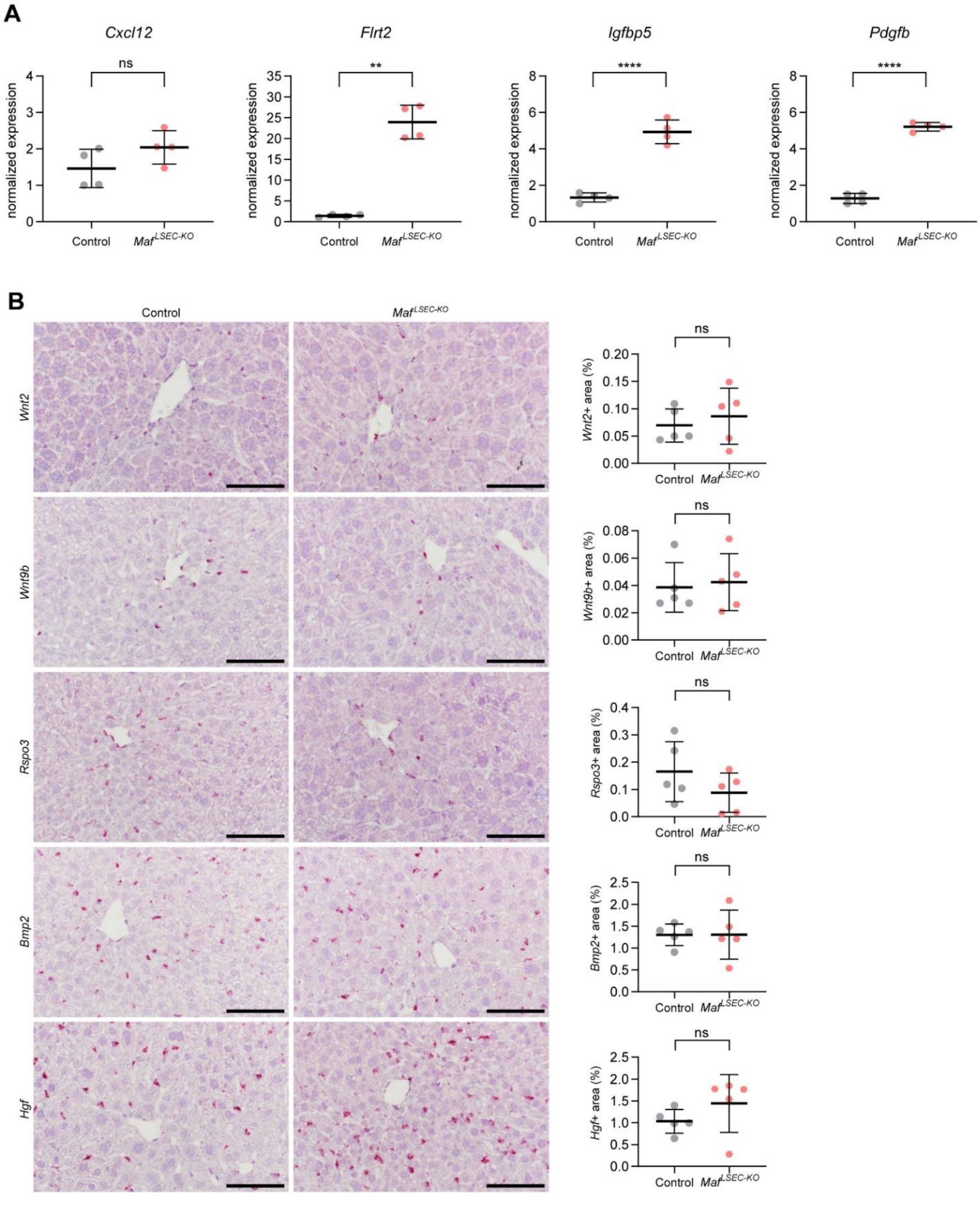

Fig. S7. *In situ* hybridization of hepatic angiocrine factors.

(A) qPCR of *Cxcl12*, *Flrt2*, *Igfbp5,* and *Pdgfb* using RNA from isolated LSEC (n = 4). (B) *In situ* hybridization and quantification for *Wnt2, Wnt9b, Rspo3, Bmp2* and *Hgf* (n = 5). Mean ± SD. (B [*Wnt2, Rspo3, Bmp2*]) Welch's *t* test. (B [Wnt9b, Hgf] Mann-Whitney *U* test. n.s. $p \geq .05$.; * $p < .05$.

Fig. S8

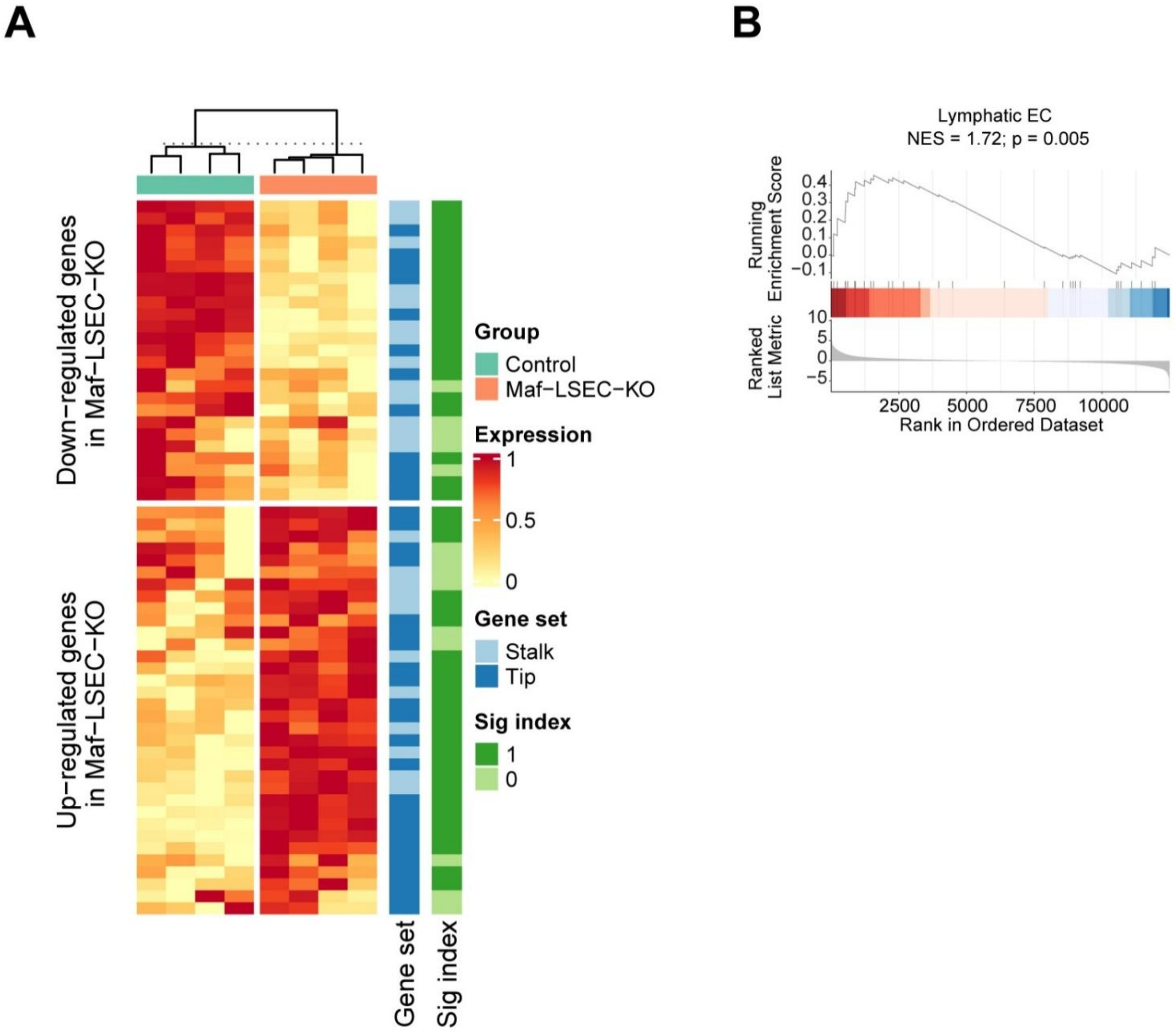

Fig. S8. Heatmap of angiogenesis related genes and enrichment analysis for lymphatic EC.

(A) Heatmap of tip and stalk cell genes in RNA-seq data from LSEC. (B) Enrichment plot for lymphatic EC associated genes. NES, normalized enrichment score.

Fig. S9

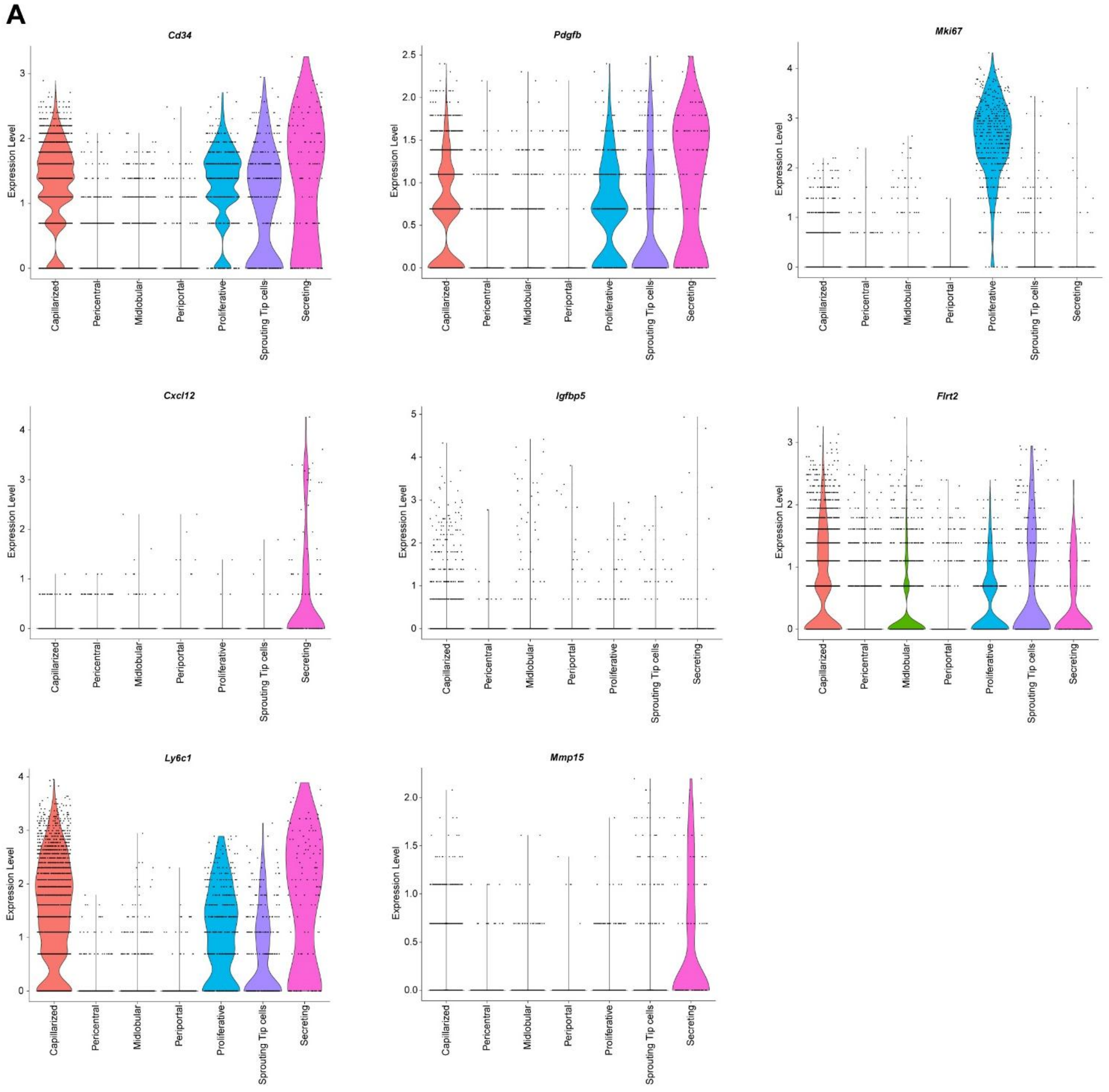

Fig. S9. Gene expressions for genes of interest in scRNA-seq of LSEC.

(A) Violin plots for the genes of interest (*Cd34*, *Pdgfb*, *Mki67*, *Cxcl12, Igfbp5*, *Flrt2, Ly6c1,* and *Mmp15*).

Fig. S10

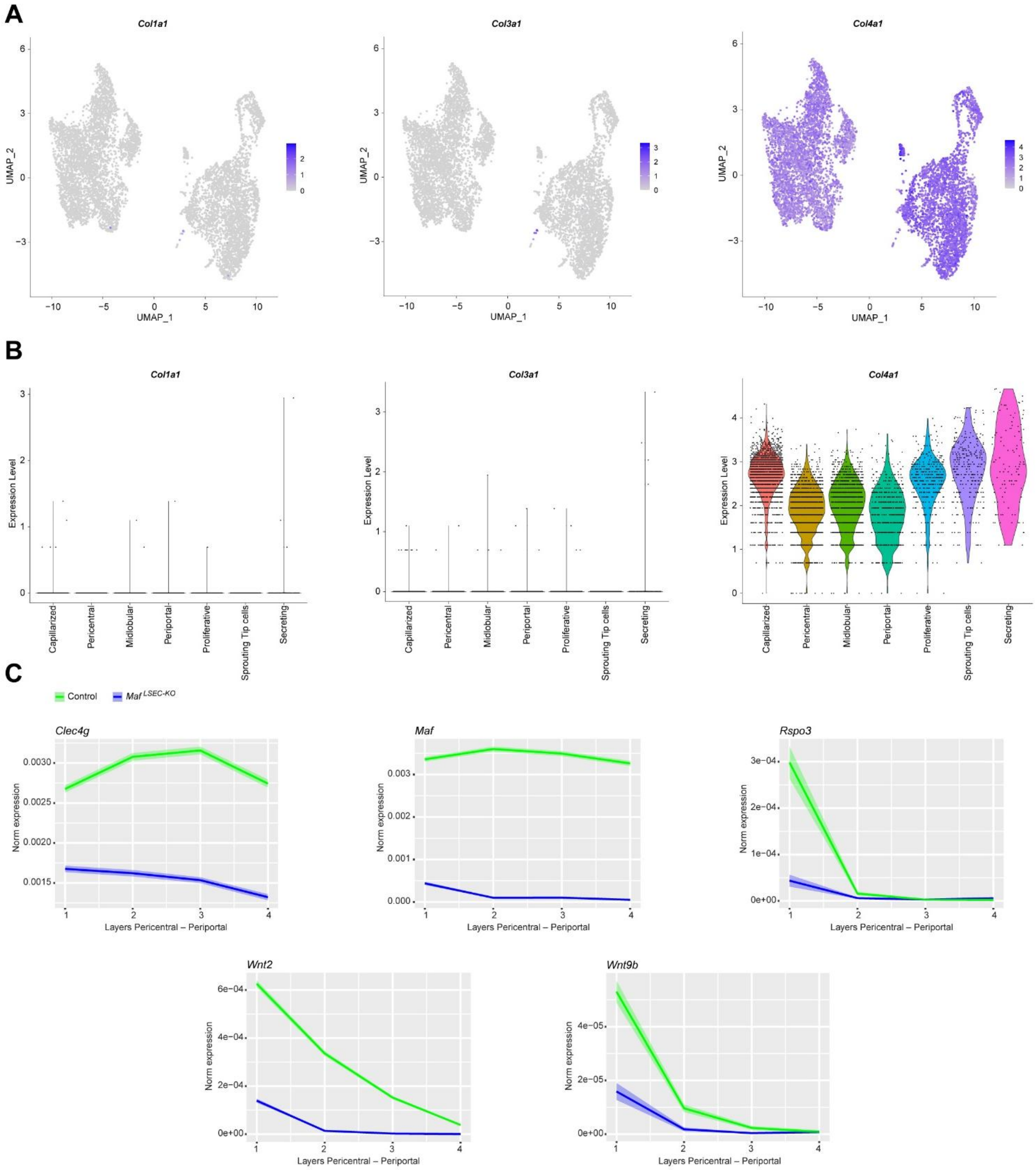

Fig. S10. Gene expressions for genes of interest and zonated expression of Wnt factors in scRNA-seq of LSEC. Annotation for the genes of interest (*Col1a1*, *Col3a1* and *Col4a1*) in (A) UMAP plot and (B) violin plot. (C) Gene expressions for *Clec4g*, *Maf*, and Wnt factors (*Rspo3*, *Wnt2*, and *Wnt9b*) in LSEC zones ranging from pericentral (1) to periportal (4).

Table S1

Results of bulk RNA-seq analysis of hepatic endothelial cells from *Maf*$^{LSEC-KO}$ vs. control mice

See separate Excel file: Table_S1_bulk-RNA-seq_LSEC_Maf-LSEC-KO_vs_Controls.xlsx

Table S2

Results of ATAC-seq analysis of hepatic endothelial cells from *Maf*$^{LSEC-KO}$ vs. control mice

See separate Excel file: Table_S2_ATAC-seq_LSEC_Maf-LSEC-KO_vs_Controls.xlsx

Table S3

TOBIAS footprint analysis

See separate Excel file:

Table_S3_ATAC-seq_LSEC_TOBIAS_footprints_Maf-LSEC-KO_vs_Controls.xlsx

Table S4

Directly c-Maf regulated genes identified by downregulated c-Maf footprints and significant dysregulation in bulk RNA-seq data of hepatic endothelial cells

See separate Excel file:

Table_S4_Genelist_c-Maf-footprint_down_and_adjp_0.05_in_bulk-RNA-seq.xlsx

Supplementary References

[1] Schneider CA, Rasband WS, Eliceiri KW. NIH Image to ImageJ: 25 years of image analysis. Nat Methods 2012;9:671–5. https://doi.org/10.1038/nmeth.2089.

[2] Schindelin J, Arganda-Carreras I, Frise E, et al. Fiji: an open-source platform for biological-image analysis. Nature Methods 2012;9:676–82. https://doi.org/10.1038/nmeth.2019.

[3] Hruz T, Wyss M, Docquier M, et al. RefGenes: identification of reliable and condition specific reference genes for RT-qPCR data normalization. BMC Genomics 2011;12:156. https://doi.org/10.1186/1471-2164-12-156.

[4] Winkler M, Staniczek T, Kürschner SW, et al. Endothelial GATA4 controls liver fibrosis and regeneration by preventing a pathogenic switch in angiocrine signaling. Journal of Hepatology 2021;74:380–93. https://doi.org/10.1016/j.jhep.2020.08.033.

[5] H Backman TW, Girke T. systemPipeR: NGS workflow and report generation environment. BMC Bioinformatics 2016;17:388. https://doi.org/10.1186/s12859-016-1241-0.

[6] Bray NL, Pimentel H, Melsted P, et al. Near-optimal probabilistic RNA-seq quantification. Nat Biotechnol 2016;34:525–7. https://doi.org/10.1038/nbt.3519.

[7] Ritchie ME, Phipson B, Wu D, et al. limma powers differential expression analyses for RNA-sequencing and microarray studies. Nucleic Acids Research 2015;43:e47. https://doi.org/10.1093/nar/gkv007.

[8] Gu Z, Eils R, Schlesner M. Complex heatmaps reveal patterns and correlations in multidimensional genomic data. Bioinformatics 2016;32:2847–9. https://doi.org/10.1093/bioinformatics/btw313.

[9] Xu S, Hu E, Cai Y, et al. Using clusterProfiler to characterize multiomics data. Nat Protoc 2024:1–29. https://doi.org/10.1038/s41596-024-01020-z.

[10] Su T, Yang Y, Lai S, et al. Single-Cell Transcriptomics Reveals Zone-Specific Alterations of Liver Sinusoidal Endothelial Cells in Cirrhosis. Cellular and Molecular Gastroenterology and Hepatology 2021;11:1139–61. https://doi.org/10.1016/j.jcmgh.2020.12.007.

[11] Corces MR, Trevino AE, Hamilton EG, et al. An improved ATAC-seq protocol reduces background and enables interrogation of frozen tissues. Nat Methods 2017;14:959–62. https://doi.org/10.1038/nmeth.4396.

[12] Heinz S, Benner C, Spann N, et al. Simple combinations of lineage-determining transcription factors prime cis-regulatory elements required for macrophage and B cell identities. Mol Cell 2010;38:576–89. https://doi.org/10.1016/j.molcel.2010.05.004.

[13] Wang Q, Li M, Wu T, et al. Exploring Epigenomic Datasets by ChIPseeker. Current Protocols 2022;2:e585. https://doi.org/10.1002/cpz1.585.

[14] Ben-Moshe S, Veg T, Manco R, et al. The spatiotemporal program of zonal liver regeneration following acute injury. Cell Stem Cell 2022;29:973-989.e10. https://doi.org/10.1016/j.stem.2022.04.008.

[15] Butler A, Hoffman P, Smibert P, et al. Integrating single-cell transcriptomic data across different conditions, technologies, and species. Nat Biotechnol 2018;36:411–20. https://doi.org/10.1038/nbt.4096.

[16] McGinnis CS, Murrow LM, Gartner ZJ. DoubletFinder: Doublet Detection in Single-Cell RNA Sequencing Data Using Artificial Nearest Neighbors. Cell Syst 2019;8:329-337.e4. https://doi.org/10.1016/j.cels.2019.03.003.

[17] Kalucka J, de Rooij LPMH, Goveia J, et al. Single-Cell Transcriptome Atlas of Murine Endothelial Cells. Cell 2020;180:764-779.e20. https://doi.org/10.1016/j.cell.2020.01.015.

[18] Halpern KB, Shenhav R, Massalha H, et al. Paired-cell sequencing enables spatial gene expression mapping of liver endothelial cells. Nature Biotechnology 2018;36:962. https://doi.org/10.1038/nbt.4231 https://www.nature.com/articles/nbt.4231#supplementary-information.

[19] Ramachandran P, Dobie R, Wilson-Kanamori JR, et al. Resolving the fibrotic niche of human liver cirrhosis at single-cell level. Nature 2019;575:512–8. https://doi.org/10.1038/s41586-019-1631-3.

[20] Hafemeister C, Satija R. Normalization and variance stabilization of single-cell RNA-seq data using regularized negative binomial regression. Genome Biology 2019;20:296. https://doi.org/10.1186/s13059-019-1874-1.

[21] Germain P-L, Lun A, Garcia Meixide C, et al. Doublet identification in single-cell sequencing data using scDblFinder. F1000Res 2021;10:979. https://doi.org/10.12688/f1000research.73600.2.

[22] Guilliams M, Bonnardel J, Haest B, et al. Spatial proteogenomics reveals distinct and evolutionarily conserved hepatic macrophage niches. Cell 2022;185:379-396.e38. https://doi.org/10.1016/j.cell.2021.12.018.

[23] Nagy D, Maude H, Birdsey GM, et al. RISING STARS: Liver sinusoidal endothelial transcription factors in metabolic homeostasis and disease. J Mol Endocrinol 2023;71:e230026. https://doi.org/10.1530/JME-23-0026.
